# Design strategies and application progress of covalent organic frameworks in photocatalytic oxidation reactions

**DOI:** 10.1039/d5sc08410e

**Published:** 2025-12-29

**Authors:** Tao Sun, Rui Wang, Renquan Guan, Li Wang, Tian Zhong, Chunbo Liu, Xueying Cheng, Qianrong Fang

**Affiliations:** a Science and Technology Innovation Center of Jilin Province for Targeted Identification and Photocatalytic Degradation Materials, College of Engineering, Jilin Normal University Siping 136000 China chunboliu@jlnu.edu.cn chengxy@jlnu.edu.cn; b Key Laboratory of Preparation and Application of Environmental Friendly Materials of the Ministry of Education, College of Chemistry, Jilin Normal University Changchun 130103 China; c State Key Laboratory of Inorganic Synthesis and Preparative Chemistry, College of Chemistry Jilin University Changchun 130012 China qrfang@jlu.edu.cn; d State Key Laboratory of Semiconductor Physics and Chip Technologies, Institute of Semiconductors, Chinese Academy of Sciences Beijing 100083 China; e School of Pharmacy, Faculty of Medicine, Macau University of Science and Technology Macau

## Abstract

Photocatalytic generation of reactive oxygen species (ROS) presents a sustainable alternative to traditional oxidation methods, offering precise control over reaction pathways for diverse applications. Covalent organic frameworks (COFs), with their crystalline porous structures and tunable electronic properties, are ideal platforms for maximizing photocatalytic ROS efficiency and selectivity. This review systematically explores the intrinsic connection between COF architecture and ROS activity, framed by a “structure–ROS–substrate” paradigm. We detail how rational design strategies—from foundational band energy engineering and π-conjugation control to functional group integration and pore microenvironment regulation—precisely modulate the material's electronic structure, charge carrier dynamics, and mass transport to govern ROS speciation and concentration. These structural innovations underpin the remarkable performance of COFs in photocatalytic oxidation, including selective organic transformations, sustainable H_2_O_2_ production, and efficient degradation of recalcitrant pollutants. The application scope is further extended to biomedical fields, leveraging ROS for antibacterial therapy and photodynamic effects. Finally, we discuss prevailing challenges in quantitative ROS detection, operational stability, and scalable synthesis, while outlining future opportunities in machine-learning-guided design and tandem catalytic systems. This review aims to establish fundamental design principles for the next generation of COF-based photocatalysts, bridging molecular-level engineering to macroscopic oxidative efficacy.

## Abstract 2

Photocatalytic generation of reactive oxygen species (ROS) presents a sustainable alternative to traditional oxidation methods, offering precise control over reaction pathways for diverse applications.

## Graphical abstract

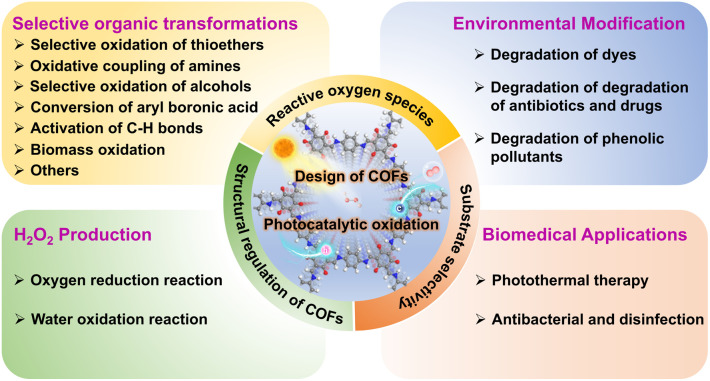

## 1 Introduction

The development of efficient and sustainable oxidation technologies is a central challenge in modern chemistry.^1^ Oxidation reactions are central to both chemical manufacturing and environmental management. They are indispensable in synthesizing fine chemicals—including key products such as pharmaceuticals and agrochemicals—as well as in producing polymers. Beyond synthesis, these reactions are fundamental to environmental remediation processes such as wastewater treatment and air purification.^2^ Conventional oxidation methods, although widely employed in industry, typically rely on stoichiometric oxidants or noble-metal catalysts.^3^ While these approaches are effective, they suffer from intrinsic limitations, including high energy consumption, low atom economy, poor selectivity, and the generation of toxic by-products.^4^ These issues not only increase operational costs but also raise severe environmental and sustainability concerns.^4^ In an era of increasing demand for green and scalable technologies, there is an urgent need to design oxidation platforms that are both efficient and environmentally benign.

Photocatalysis offers a compelling solution by harnessing sunlight—a clean, abundant, and renewable energy source—to drive redox processes.^5^ In particular, photocatalytic oxidation mediated by reactive oxygen species (ROS) such as hydroxyl radicals (·OH), superoxide radicals (·O_2_^−^), singlet oxygen (^1^O_2_), and hydrogen peroxide (H_2_O_2_) has emerged as a versatile and sustainable alternative to conventional oxidation chemistry.^6^ The selective generation and utilization of ROS under mild conditions not only lowers the energy threshold of transformations but also provides a handle to regulate product selectivity, which is critical for pharmaceutical synthesis and pollutant degradation.^7^ Beyond its practical advantages, ROS chemistry also represents a model system for exploring fundamental concepts in photochemistry, electron transfer, and interfacial catalysis.^8^

Covalent organic frameworks (COFs), a rapidly expanding class of crystalline porous polymers, have recently attracted tremendous attention as photocatalytic platforms.^9^ Distinguished by their ordered π-conjugated skeletons, modular design, and robust structural stability, COFs offer an unprecedented level of tunability in photocatalysis. Unlike traditional inorganic photocatalysts, COFs allow precise integration of donor–acceptor (D–A) architectures, extension of π-conjugation, and incorporation of heteroatoms or metal centres into their frameworks.^10^ These features enable rational bandgap tuning, efficient charge separation, and targeted engineering of catalytic sites. Importantly, the inherent porosity of COFs facilitates mass transport, while their crystallinity allows detailed mechanistic studies that link structure with function.^11^ This combination of modularity, crystallinity, and tunability distinguishes COFs as an ideal platform to systematically study and optimize photocatalytic oxidation.^12^

Over the past decade, COF-based photocatalysis has witnessed remarkable growth, particularly in oxidation chemistry.^13^ Representative advances include the selective oxidation of sulfides to sulfoxides, a key transformation in drug and fine chemical synthesis; oxidative coupling of amines to imines, important intermediates for pharmaceuticals and agrochemicals; aerobic oxidation of alcohols to aldehydes or ketones; and the activation of inert C–H bonds, which opens new avenues for late-stage functionalization of complex molecules.^14^ In parallel, COFs have been successfully applied to environmental remediation, where their ability to regulate ROS species enables the degradation of dyes, antibiotics, and emerging pollutants with high selectivity and minimal secondary contamination.^15^ Another rapidly expanding frontier is the photocatalytic production of H_2_O_2_, where COFs have demonstrated significant potential in addressing the kinetic limitations of the water oxidation reaction (WOR) and oxygen reduction reaction (ORR).^16^ By simultaneously activating ORR and WOR pathways, COFs can achieve efficient overall H_2_O_2_ production directly from water and oxygen, offering an attractive alternative to the energy-intensive anthraquinone process.^17^ Furthermore, COFs are increasingly explored in biomedical contexts, such as photodynamic therapy (PDT) and antibacterial disinfection, where controlled ROS generation under visible or near-infrared light enables targeted therapeutic applications.^18^

However, the true conceptual power and guiding significance of this “structure–ROS–substrate” paradigm extend beyond cataloguing these individual correlations. It lies in its capacity to serve as a unifying and predictive framework that advances beyond traditional analytical lenses. Conventional frameworks such as band theory or active-site analysis offer invaluable yet compartmentalized insights: the former excels at describing photophysical initiation, while the latter focuses on terminal chemical events at localized sites.^19,20^ In contrast, the “structure–ROS–substrate” paradigm integrates these aspects into a holistic, dynamic continuum. It uniquely posits that the photocatalytic outcome is an emergent property arising from the continuous interplay among the three vertices of the triad. Therefore, this paradigm does not merely describe structure–property relationships; it provides a causal lens to interpret complex reaction selectivity, reconcile seemingly contradictory literature findings, and, most importantly, guide the rational design of COFs by targeting the optimization of this interplay.

Beyond practical demonstrations, these advances have also deepened fundamental insights into the so-called “structure–ROS–substrate” paradigm. It is now clear that electronic configuration, porosity, interfacial interactions, and functional group distribution within COFs collectively determine ROS speciation and reactivity. For instance, the orientation of linkage bonds can alter dipole moments and influence charge separation efficiency,^21^ heteroatom doping can accelerate intersystem crossing (ISC) to boost ^1^O_2_ generation,^22^ and pore-size engineering can dictate substrate accessibility and product selectivity.^23^ Such molecular-level control is rarely achievable in conventional photocatalysts, underscoring the unique role of COFs in bridging fundamental photochemistry with practical oxidation catalysis. Despite significant progress, a clear, mechanistic, and ultimately predictive understanding of the intrinsic correlations between the COF structure and photocatalytic oxidation performance is still developing. This incomplete knowledge base is the root cause of several persistent challenges. First, the synthesis of many high-performance COFs often relies on harsh conditions or specialized monomers, complicating scalable and sustainable manufacturing. Second, the long-term structural and functional stability of these frameworks in the harsh oxidative environments inherent to photocatalysis—particularly in acidic, basic, or biologically relevant media—requires systematic improvement. Finally, the translation of laboratory performance to practical applications necessitates that COFs retain their functionality in complex, real-world matrices containing competing species and under variable operational conditions. Therefore, to advance the field, a dual focus is imperative. A deep, fundamental elucidation of “structure–mechanism–activity” relationships—achieved through advanced *in situ* characterization, computational modelling, and mechanistic studies—must guide the applied engineering efforts aimed at overcoming challenges in scalability, stability, and performance in complex environments. It is through these concerted and complementary efforts that COF-based photocatalytic oxidation can evolve from a promising laboratory discovery into viable technology for sustainable synthesis, environmental remediation, and biomedical applications.

In this review, we aim to provide a comprehensive overview of recent advances in COF-based photocatalytic oxidation, with a particular emphasis on the structural design principles that govern ROS generation and reactivity. Crucially, we will thread this discussion through the unifying perspective of the “structure–ROS–substrate” paradigm, demonstrating how it offers a coherent framework to organize existing knowledge and inspire future design. We begin by outlining the fundamental mechanisms of ROS generation in COFs, examining how intrinsic structural features such as the degree of π-conjugation set key photophysical parameters like band alignment and exciton binding energy. These parameters fundamentally govern the subsequent efficiency of dynamic processes—including light absorption, exciton dissociation, and charge separation—which collectively dictate the pathways, yields, and selectivity of ROS formation. We then discuss structural engineering strategies—including D–A integration, functional group modification, metal incorporation, and topology/porosity control—that have been developed to enhance photocatalytic efficiency. Building on this foundation, we provide a reaction-oriented analysis of COF applications across four major areas: selective organic transformations, environmental pollutant degradation, H_2_O_2_ production, and biomedical disinfection/therapy. Finally, we discuss the remaining challenges and future opportunities for rational COF design, including expanding light harvesting into the near-infrared region, achieving enantioselective oxidation, and developing scalable synthetic protocols. By offering both a mechanistic framework and a synthesis of the latest advances, this review seeks to inspire further innovation in the design of COFs for photocatalytic oxidation. We anticipate that continued progress in this area will not only expand the scope of green oxidation chemistry but also accelerate the translation of COF-based materials into practical applications ranging from chemical manufacturing to environmental protection and healthcare.

## 2 Mechanistic fundamentals of ROS generation in COFs

### 2.1 Photocatalytic processes in COFs

Photocatalysis is a process that utilizes light energy to drive chemical reactions. Its core mechanism involves the generation, separation, and migration of photogenerated electron–hole pairs, followed by redox reactions occurring on the catalyst surface.^24^ COFs exhibit significant advantages in the field of photocatalysis due to their highly ordered pore structures, tunable band structures, excellent charge transport properties, and good stability. Its photocatalytic process mainly encompasses the following key steps:

#### 2.1.1 Light absorption and charge excitation

**Fig. 1:**
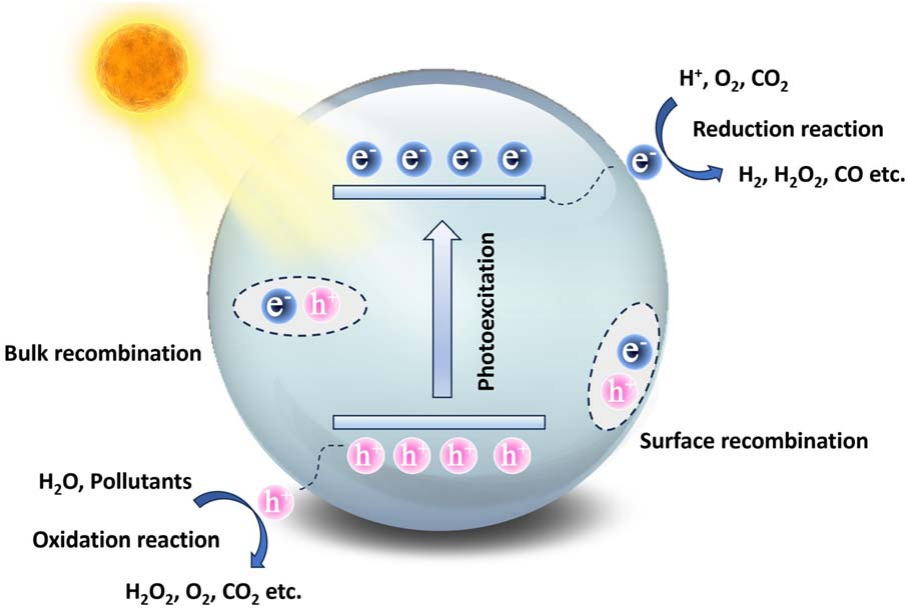
Photocatalytic mechanism of COFs.

As shown in Fig. 1, when COFs are irradiated by photons with energy equal to or greater than their bandgap, the electrons (e^−^) in the Valence Band (VB) are excited to the Conduction Band (CB), leaving holes (h^+^) in the VB, forming e^−^–h^+^ pairs. Subsequently, the photogenerated e^−^–h^+^ pairs separate and migrate from the bulk of the catalyst to the surface. During this process, some e^−^ and h^+^ will recombine in the bulk or on the surface of the catalyst, while the remaining carriers will transfer to the material's surface, forming ROS with redox capabilities, thus achieving spatial separation and transport. The e^−^ in the CB accumulated at the active sites on the catalyst surface participate in reduction reactions, while the h^+^ in the VB participate in oxidation reactions.^25,26^

#### 2.1.2 Electron–hole separation and transport

The separation efficiency of photogenerated e^−^ and h^+^ is a key factor determining photocatalytic performance. The highly ordered two-dimensional or three-dimensional π-stacked structures in COFs provide ideal channels for exciton dissociation and carrier migration. For instance, through rational structural design (such as introducing imine bonds, triazine rings, or β-ketoenamine structures), electron delocalization within the framework can be effectively promoted, reducing the recombination probability of electron–hole pairs.^27^

#### 2.1.3 Interfacial reactions and substrate oxidation

The generated ROS react with organic substrates adsorbed on the COF surface, achieving selective conversion or complete mineralization. The high specific surface area and adjustable pores of COFs facilitate the adsorption and diffusion of reactants, providing abundant active sites for substrates, thereby promoting interfacial reactions. Meanwhile, the structural tunability allows for precise control of specific reaction pathways by introducing specific functional groups (such as sulfonic acid groups, phenolic hydroxyl groups, metal coordination sites, *etc.*).^28^

#### 2.1.4 Regulation of photocatalytic processes by the structure

The structural characteristics of COFs (such as connectivity, dimensionality, crystallinity, specific surface area, *etc.*) directly affect their photocatalytic performance. For example, sp^2^ carbon-connected COFs have fully conjugated frameworks, exhibiting excellent charge transport properties;^29^ meanwhile, metal doping or constructing heterojunctions (such as Z-type or S-type heterojunctions) can further promote charge separation, enhancing ROS generation efficiency and reaction selectivity.^30^ Therefore, a deep understanding of the correlation between the mechanism of ROS generation and the band structure of COFs is crucial.

### 2.2 Pathways of ROS generation

In photocatalytic oxidation reactions, the generation of ROS is central to achieving efficient oxidation reactions. The generation pathways are complex, multi-step processes that depend on the interactions between photogenerated e^−^ and h^+^ with adsorbed O_2_ or H_2_O molecules, leading to the formation of various ROS. The main ROS include ·O_2_^−^, ·OH, ^1^O_2_, and H_2_O_2_. The generation pathways of these ROS are interconnected, forming a complex reaction network. Their types and concentrations are significantly influenced by the band structure of COFs, surface chemical properties, and reaction environment.^31,32^

#### 2.2.1 Generation of ·O_2_^−^

·O_2_^−^ is a common and key reactive species in photocatalytic processes, typically generated by the single-electron reduction of O_2_ by e^−^ in the CB (eqn (1)). The thermodynamic feasibility of this process requires the Conduction Band Minimum (CBM) of COFs to be more negative than the reduction potential of O_2_/·O_2_^−^ (approximately −0.33 V *vs.* NHE).^33^

1 O_2_ + e^−^ → ·O_2_^−^

The band structure of COFs can be precisely tuned through the linkage chemistry and the energy levels of the building units. For example, COFs constructed with strong electron-donating units (such as pyrene and porphyrin) typically have more negative CB positions, providing a stronger thermodynamic driving force for O_2_ reduction.^34^ Additionally, highly extended π-conjugated structures and ordered pores facilitate the rapid migration of photogenerated e^−^, effectively suppressing electron–hole recombination and enhancing the generation efficiency of ·O_2_^−^.^35^ Surface engineering also plays a crucial role in the generation of ·O_2_^−^. Introducing electron-rich functional groups or metal nodes (such as Co and Fe) can act as electron relay stations to facilitate electron transfer and enhance the adsorption and activation of O_2_, further improving the generation rate and stability of ·O_2_^−^.^36^

#### 2.2.2 Generation of H_2_O_2_

H_2_O_2_ is a relatively stable and environmentally friendly oxidant with significant practical applications. It is mainly produced through two pathways, namely the ORR and the WOR. Due to the lower reaction barrier of ·O_2_^−^, ORR predominates in the generation of H_2_O_2_. There are two reduction pathways for generating H_2_O_2_ from ·O_2_^−^. One is the dismutation of the electron-reduced species ·O_2_^−^ (eqn (2)), and the other is the reduction of ·O_2_^−^ by photogenerated e^−^ in the CB (eqn (3)). In the WOR pathway, H_2_O_2_ can be produced through the direct oxidation of water molecules (eqn (4)) or the coupling of ·OH (eqn (5) and (6)).^37^

2 ·O_2_^−^ + ·O_2_H + H_2_O → H_2_O_2_ + O_2_ + OH^−^

3 ·O_2_^−^ + e^−^ + 2H^+^ → ·H_2_O_2_

4 2H_2_O + 2h^+^ → H_2_O_2_ + 2H^+^

5 H_2_O + h^+^ → ·OH + H^+^

6 ·OH + ·OH → H_2_O_2_

The unique, programmable architecture of COFs offers exceptional molecular-level control over the selectivity and efficiency of H_2_O_2_ production. Photocatalytic H_2_O_2_ synthesis predominantly proceeds *via* two pathways: (1) the two-electron oxygen reduction reaction (2e^−^ ORR), where O_2_ is reduced by photogenerated electrons, and (2) the water oxidation reaction (WOR), where H_2_O is oxidized by photogenerated holes. The central challenge lies in selectively promoting the 2e^−^ ORR over the competing four-electron (4e^−^) pathway to H_2_O, and in efficiently activating the thermodynamically demanding WOR, which is critical for achieving overall photosynthetic H_2_O_2_ generation from H_2_O and O_2_ without sacrificial agents.

COFs address these challenges through precise structural and electronic engineering that targets the key reaction intermediates. For the 2e^−^ ORR pathway, the selectivity is governed by the binding strength of the *OOH intermediate. COFs can be designed to favor the 2e^−^ pathway by modulating their electronic structure, for example, through donor–acceptor (D–A) motif engineering, which optimizes the adsorption free energy of *OOH to a value close to the thermodynamic optimum. Furthermore, the strategic incorporation of heteroatoms (*e.g.*, boron and sulfur) or single-metal sites (*e.g.*, Co and Ni) can create defined Lewis acid or redox-active centers that selectively stabilize the *OOH intermediate, thereby suppressing its further reduction to H_2_O. For the WOR pathway, the oxidation of H_2_O to H_2_O_2_ involves complex multi-step proton-coupled electron transfers. COFs can facilitate this process through functional group engineering; for instance, hydrophilic –OH or –COOH groups can enhance water adsorption and local proton concentration, while specific organic moieties can act as co-catalysts to lower the kinetic barrier for water oxidation. Beyond active site design, the porous and ordered structure of COFs enhances mass transport and enriches reactants (O_2_ and H_2_O) near active sites. Concurrently, their extended π-conjugation systems promote efficient light absorption and long-range charge carrier separation, ensuring a sufficient flux of electrons and holes to drive the respective reduction and oxidation reactions. The synergistic interplay of these structural elements enables COFs to achieve superior efficiency and selectivity in photocatalytic H_2_O_2_ synthesis compared to many conventional materials.^37^

#### 2.2.3 Generation of ^1^O_2_

^1^O_2_ is a highly selective oxidant with widespread applications in organic synthesis and biomedicine. Its generation mechanisms mainly include energy transfer and post-electron transfer oxidation pathways. In the energy transfer pathway, excited COFs (either singlet or triplet states) transfer energy to ground state ^3^O_2_, promoting it to the excited state ^1^O_2_. In the electron transfer pathway, the generated ·O_2_^−^ can be further oxidized by h^+^ to produce ^1^O_2_. Highly conjugated π systems facilitate exciton delocalization and extend the triplet state lifetime, greatly enhancing the energy transfer process. Designing D–A type COFs is an effective strategy to enhance ^1^O_2_ generation.^38^ The strong intramolecular charge transfer (ICT) effect not only broadens the light absorption spectrum but also promotes ISC, increasing the triplet exciton yield and providing a rich basis for energy transfer. Additionally, the rigid porous framework of COFs can isolate the generated ^1^O_2_, thereby slowing down its quenching, extending its lifetime, and providing more opportunities for it to react with target substrates.^39^

#### 2.2.4 Generation of ·OH

·OH is a highly oxidative and non-selective ROS, typically responsible for the deep mineralization of organic pollutants. Its generation primarily relies on the strong oxidizing ability of VB h^+^. The generation of ·OH mainly depends on the oxidation of H_2_O molecules or surface hydroxyl groups (OH^−^) by h^+^ (eqn (7)). This process requires the Valence Band Maximum (VBM) energy level of COFs to be more positive than the oxidation potential of ·OH/OH^−^ (approximately +1.99 V *vs.* NHE).

7 H_2_O + h^+^ → ·OH + H^+^

However, due to its high potential, the direct hole oxidation pathway is usually limited. Under acidic conditions or in the presence of metal sites (such as in Fenton-like reactions), H_2_O_2_ can be homolytically reduced by e^−^ to generate ·OH (eqn (8)).

8 H_2_O_2_ + e^−^ → ·OH + OH^−^

The abundant functional sites on the surface of COFs (such as hydroxyl and carboxyl groups) and metal nodes introduced through post-modification (such as Fe and Cu) can effectively regulate interfacial charge separation and participate in the generation pathway of ·OH.^40^

## 3 Structure–activity relationships in ROS regulation

### 3.1 Linkage chemistry and framework stability

The photocatalytic performance of COFs is fundamentally governed by their molecular structure, with linkage chemistry serving as a key structural determinant.^41^ The choice of covalent linkages connecting the organic building units not only determines the material's crystallinity and porosity, but also directly impacts the extent of π-conjugation, thermodynamic and kinetic stability, and electronic band structure, ultimately affecting the separation efficiency of photogenerated charge carriers and the generation of ROS.^42^ Consequently, strategic linkage design forms the foundation of the “structure–ROS–substrate” paradigm for constructing stable, high-performance COF photocatalytic platforms.

Common linkage types in COFs include boronic ester, imine, triazine, hydrazone, and sp^2^ carbon linkages.^43^ The chemical stability and electronic properties of each linkage type differ significantly, which directly determines their suitability in complex photocatalytic oxidation environments, particularly when involving the generation and consumption of ROS.

Boronic ester-linked COFs are among the first successfully demonstrated crystalline COFs and possess excellent thermal stability. However, their susceptibility to hydrolysis in moist environments is even more pronounced, often leading to the loss of porosity and crystallinity, which restricts their application in photocatalytic reactions.^44^ Furthermore, imine-linked COFs formed *via* reversible Schiff base condensation are among the most widely studied types due to their relatively mild synthesis conditions, high crystallinity, and large specific surface area. The C=N bond imparts a polarized character that can facilitate intramolecular charge transfer, favouring light absorption. However, their dynamic covalent nature often compromises hydrolytic stability, especially during prolonged operation in aqueous or acidic oxidative environments, limiting their long-term recyclability.^45^ Other linkages such as triazine and hydrazone present distinct trade-offs in stability, synthesis conditions, and electronic properties.^43^

To address these stability challenges, research has focused on designing irreversible or highly stable linkage types. Among these, fully conjugated sp^2^ carbon-linked COFs constructed *via* irreversible reactions such as Knoevenagel condensation represent a significant advancement.^46^ These fully conjugated frameworks, such as vinylene-linked or sp^2^c-COFs, demonstrate exceptional chemical stability against hydrolysis, acids, and bases, attributable to the robust carbon–carbon bonds. More importantly, the extended and fully delocalized π-conjugation system significantly enhances charge carrier mobility, reduces exciton binding energy, and improves visible light absorption, thereby leading to superior photocatalytic activity and long-term operational stability.^47^

Therefore, linkage chemistry is not merely a structural detail but a core design parameter in the “structure–ROS–substrate” paradigm. The development of COFs with highly stable and fully conjugated linkages is crucial for advancing photocatalytic oxidation technologies. Simultaneously, post-synthetic modification strategies that transform hydrolytically labile imine bonds into more stable chemical bonds have become an effective means to enhance the stability of imine-based COFs. Through targeted selection and engineering of the linkage type, the electronic structure, exciton dynamics, and internal chemical environment of COFs can be precisely controlled, laying a solid foundation for subsequent bandgap engineering, D–A design, and pore optimization.

### 3.2 Band gap and energy level engineering

The photocatalytic performance of COFs is closely related to their electronic structure, particularly the band gap width and the positioning of energy levels (such as HOMO/LUMO or VB/CB), which have a decisive impact on the generation pathways and efficiency of ROS. Through rational structural design, especially by matching the linker chemistry with the electronic properties of the building units, precise control over the band structure of COFs can be achieved, thereby optimizing the separation efficiency of photogenerated carriers and the selective generation of ROS.^48^

**Fig. 2:**
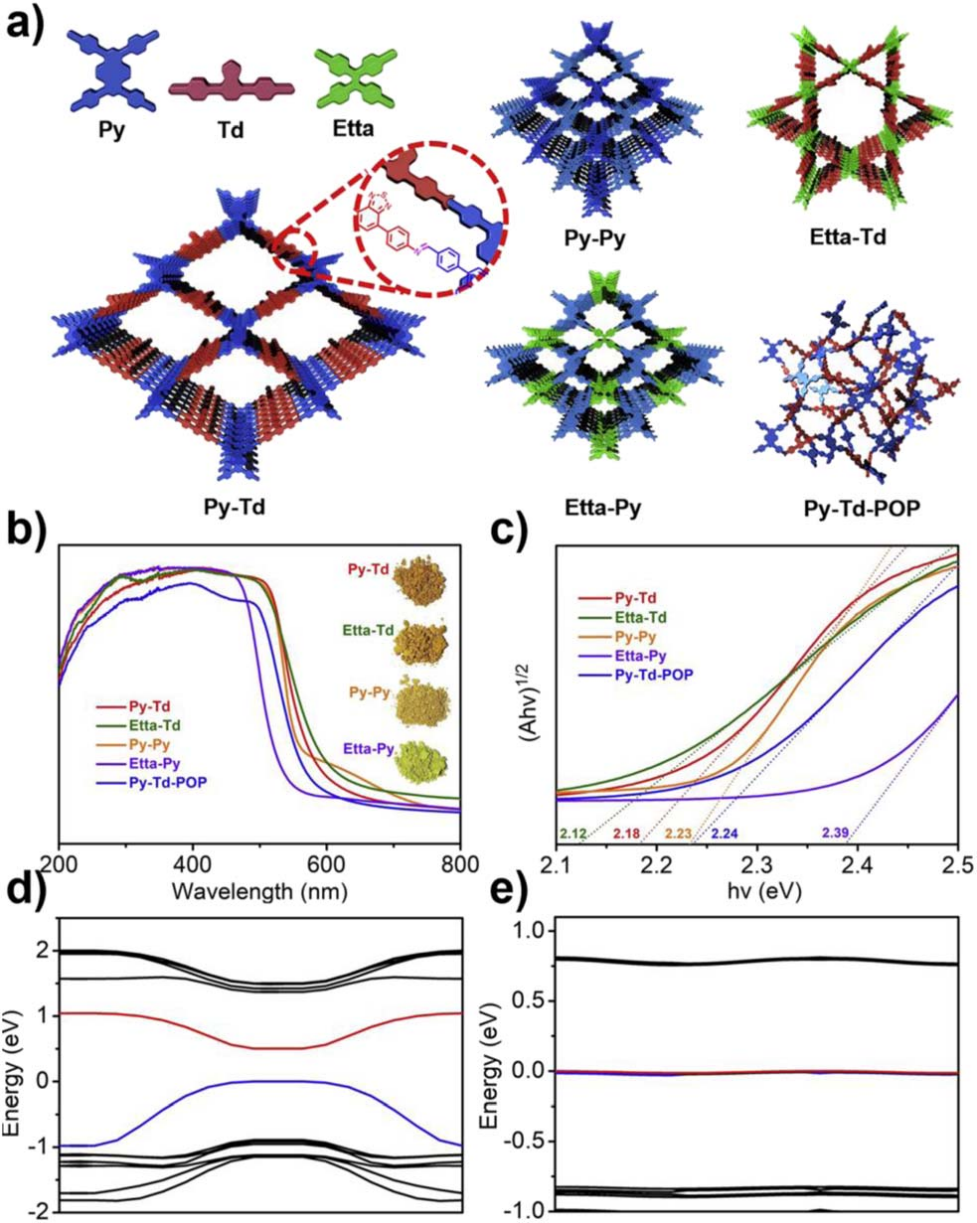
(a) Amine linkage-based connection structures and their corresponding synthesized materials; (b and c) optical properties of the materials; (d) band structure diagrams and (e) symmetry–performance relationship studies of Py–Td and Etta–Td COFs. Reproduced with permission from ref. 49, Copyright 2019, from Elsevier.

Wang *et al.* systematically studied a series of COFs connected by imine bonds (such as Py–Py, Etta–Py, Py–Td, and Etta–Td), revealing the significant regulatory effect of the electronic properties of the building units on the optical band gap of the materials.^49^ As shown in Fig. 2, the introduction of electron-withdrawing thiazole (Td) units significantly reduces the band gap of the COFs (Py–Td: 2.18 eV; Etta–Td: 2.12 eV), while purely donor-type building units (such as Py–Py) exhibit a wider band gap (2.39 eV). This narrowing of the band gap is mainly attributed to the enhanced D–A type charge transfer effect, thereby extending the material's absorption range in the visible light region. Furthermore, the regulation of energy level positions is equally crucial as it directly determines the reduction/oxidation capabilities of photogenerated e^−^ and h^+^, thus governing the pathways of ROS generation. Through Mott–Schottky tests and cyclic voltammetry (CV) measurements, researchers found that all tested COFs exhibited p-type semiconductor characteristics. Among them, the CB positions of Py–Td and Etta–Td are located at −0.34 V and −0.68 V (*vs.* NHE), respectively, while the VB positions are at 1.84 V and 1.44 V, respectively. These energy level positions directly influence their reduction/oxidation capabilities, thereby determining the pathways of ROS generation (such as ·O_2_^−^*vs.*^1^O_2_).

Crystallinity also has a significant impact on the band structure. Although the amorphous analogue Py–Td–POP has the same chemical composition as Py–Td, its band gap is wider (2.24 eV) and its visible light absorption capacity is significantly reduced, further confirming the critical role of long-range ordered structures in promoting π-conjugation extension and charge delocalization. Materials with poor crystallinity typically have more defect states, which can act as charge recombination centres, reducing the efficiency of carrier separation and ultimately weakening the ability to generate ROS.^49^

In summary, band engineering is a core strategy for regulating the photocatalytic ROS generation behaviour of COFs. By reasonably selecting donor and acceptor building units with specific electronic properties, the band gap of the material can be effectively narrowed, the light response range can be broadened, and the energy level positions can be precisely controlled, thereby thermodynamically creating conditions for the generation of specific ROS. Meanwhile, constructing a highly crystalline framework structure is the kinetic foundation for achieving efficient charge separation and migration, which is crucial for ensuring photocatalytic activity.

### 3.3 Tuning charge delocalization *via* π-conjugation engineering

The highly ordered and extended π-conjugated framework in COFs is not only the structural basis for efficient light harvesting but also the key to promoting the separation and migration of photogenerated charges. By carefully designing building units to construct strong D–A systems or introducing specific functional groups to enhance intramolecular electronic coupling, the exciton dynamics (such as promoting ISC) and charge delocalization behaviour can be effectively regulated, thereby significantly influencing the pathways and efficiency of ROS generation.

π-Conjugated systems have multiple impacts on charge separation efficiency. Firstly, an extended π-conjugated system can broaden the light absorption range of COFs, allowing them to capture more photon energy. For example, pyrene-based COFs achieve a blue shift in the absorption edge through π-conjugation expansion, enhancing their response to visible light and providing more excitation sources for charge separation.^50^ Secondly, a π-conjugated network helps in the delocalization of e^−^ within and between molecules, forming delocalized π-bonds. This reduces the charge transport barrier and promotes the separation and transport of e^−^ and h^+^.^51^ In D–A type COFs, π-conjugated linkers can guide e^−^ to transfer directionally from donor units to acceptor units, further improving charge separation efficiency. Additionally, the ordered stacked structure formed by strong π–π interactions can facilitate charge delocalization, reducing the likelihood of electron–hole recombination.^52^

**Fig. 3:**
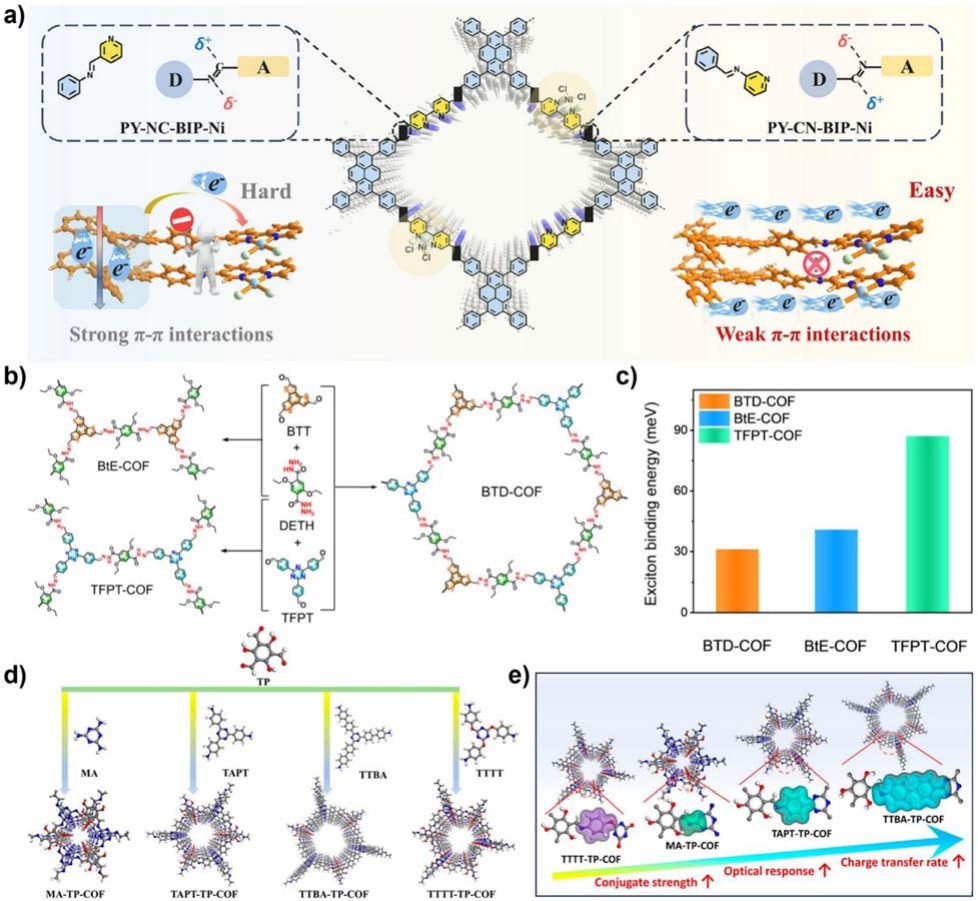
(a) Schematic diagram of interlayer and intralayer charge transfer in imine-based COFs; reproduced with permission from ref. 53, Copyright 2025, from Wiley-VCH. (b) Synthesis diagrams of BTD-COF, BtE-COF, and TFPT-COF, and (c) their corresponding exciton binding energies; reproduced with permission from ref. 54, Copyright 2025, from American Chemical Society. (d) Synthesis diagrams of triazine-based COFs with different degrees of conjugation; (e) relationship between the degree of conjugation and photocatalytic performance of COFs. Reproduced with permission from ref. 55, Copyright 2025, from American Chemical Society.

Qin and colleagues systematically studied the effects of interlayer π–π interactions on photogenerated charge separation and transport by designing two pyrene-based COF isomers with opposing imine bridge orientations (PY-CN-BIP-Ni and PY-NC-BIP-Ni). Theoretical calculations and experimental results indicate that the asymmetric charge distribution in PY-CN-BIP-Ni, where the donor is attached to imine carbon (C^*δ*+^) and the acceptor to nitrogen (N^*δ*−^), effectively weakens interlayer electronic coupling. This suppression of interlayer exciton formation and energy loss localizes the electron cloud within the layers, promoting efficient charge transfer along the D–A axis to the Ni active centre. In contrast, strong interlayer π-stacking in PY-NC-BIP-Ni leads to electron delocalization across layers, forming exciplexes and hindering intralayer charge separation (Fig. 3a).^53^ As shown in Fig. 3b and c, Professor Li's team proposed a “linker unit engineering” strategy, inserting a polarized acylhydrazone unit as a π-spacer between the electron donor (benzotrithiophene) and acceptor (triazine). This induces a unique “sequential electron transfer” mechanism under illumination, effectively avoiding the rapid charge recombination common in traditional D–A structures. This significantly enhances charge delocalization, extending the excited state lifetime to 1.35 ns and greatly reducing the exciton binding energy (31.1 meV), thus achieving efficient spatial separation of e^−^ and h^+^.^54^

In regulating ROS generation, π-conjugated systems also play a crucial role. Firstly, their charge delocalization properties can adjust the local charge distribution around active sites. The extended π-conjugation can increase the charge density around active sites, enhancing the adsorption and activation of reactants, thereby indirectly influencing ROS generation. Secondly, the electronic structure of π-conjugated networks can regulate the stability of reaction intermediates, prolong the lifetime of charge-separated states, stabilize key intermediates during reactions, and thus promote the sustained generation of ROS. Professor Wang's team systematically constructed triazine-based COFs with different types (π–π *vs.* p–π) and degrees of conjugation to deeply reveal the regulatory mechanisms of π-conjugated networks on photocatalytic performance (Fig. 3d and e). The study shows that TTBA–TP-COF, with a strong π–π conjugated network, significantly enhances light response and provides efficient charge transport channels and electron-rich sites, thus exhibiting optimal charge separation efficiency (with the highest carrier mobility and the longest fluorescence lifetime up to 0.95 µs) and interfacial electron transfer capability. These features collectively contribute to its excellent interfacial electron transfer capability and ROS generation efficiency in the photocatalytic ORR.^55^

### 3.4 Functional groups and metal integration

Introducing specific functional groups or metal nodes into COFs is a key strategy for molecular-level regulation of their photocatalytic performance, particularly for the precise control of ROS generation pathways and efficiency. Such modifications can effectively tune the local electronic structure and chemical microenvironment of active sites, thereby influencing substrate adsorption behavior, charge transfer pathways, and the stability of key intermediates, ultimately achieving directed promotion of specific ROS generation pathways.

**Fig. 4:**
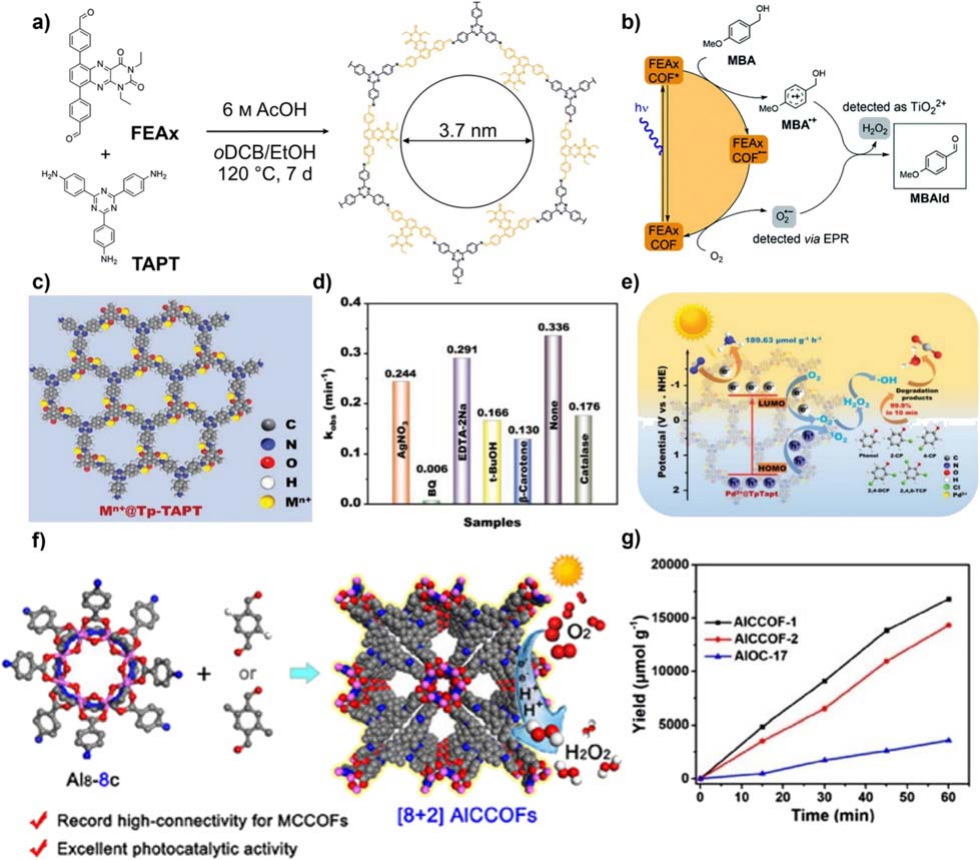
(a) Synthesis of FEAx-COF; (b) photocatalytic oxidation mechanism of MBA by FEAx-COF; reproduced with permission from ref. 56, Copyright 2021, from Author(s). (c) Molecular structure of M^*n*+^@Tp–TAPT; (d) degradation kinetics rate constants of M^*n*+^@Tp–TAPT; (e) photocatalytic degradation mechanism of M^*n*+^@Tp–TAPT; reproduced with permission from ref. 57, Copyright 2023, from Wiley-VCH. (f) Synthesis and photocatalytic mechanism diagram of AlCCOF-1/2; (g) comparison of the photocatalytic H_2_O_2_ production performance of AlCCOF-1, AlCCOF-2, and AlOC-17. Reproduced with permission from ref. 58, Copyright 2025, from Wiley-VCH.

Integrating bio-inspired groups with inherent redox activity into the COF is an effective approach to constructing efficient and highly selective photocatalytic systems. Inspired by natural flavin cofactors, Trenker *et al.* designed and synthesized a COF (FEAx-COF) containing alloxazine structural units (Fig. 4a and b). Alloxazine itself is a known organic photocatalyst, and when integrated into an ordered two-dimensional framework, its photocatalytic functionality is retained and significantly enhanced. This material not only retains the single/double electron transfer capability of alloxazine, but also broadens the visible light absorption range to 650 nm due to the extended conjugation within the framework, outperforming its molecular form. Research indicates that FEAx-COF can simultaneously generate ·O_2_^−^ and ^1^O_2_ during the photocatalytic process, with both species working synergistically in the alcohol oxidation reaction to produce aldehydes and H_2_O_2_. This indicates that the presence of the alloxazine functional group directly determines the generation of ROS through a mixed pathway that combines electron transfer and energy transfer, thereby driving efficient and highly selective alcohol oxidation reactions. Moreover, anchoring alloxazine within the COF effectively suppresses the aggregation of its molecular form in solution, allowing FEAx-COF to exhibit excellent and stable catalytic performance across various solvents, as well as good recyclability. Compared to reference COFs that do not contain alloxazine, its activity is significantly higher, confirming that this functional group is a crucial source of catalytic activity.^56^

Metal integration is another effective strategy to enhance the photocatalytic ROS generation activity of COFs. Introducing metal ions or metal clusters into the COF or pores can create Lewis acid sites, optimizing the adsorption and activation of O_2_, and facilitating the transfer and separation of photogenerated e^−^, thereby synergistically enhancing the reactivity and selectivity of ROS. As shown in Fig. 4c–e, Yang and colleagues developed a type of metal-COF single-atom catalyst by accurately anchoring metal single atoms (such as Pd^2+^, Cu^2+^, and Fe^3+^) within imine-linked COFs (Tp–TAPT). This strategy not only successfully introduced abundant Lewis acid sites, significantly enhancing the material's adsorption capacity for Lewis basic gases such as O_2_, but also positioned the metal sites as efficient centres for electron capture and transfer, greatly improving the separation and migration efficiency of photogenerated e^−^, while also collaborating with the inherent π-conjugated structure of the COFs to create a fast electron transport channel. Notably, the optimized Pd^2+^@Tp–TAPT catalyst can direct molecular oxygen along a highly selective route (O_2_ → ·O_2_^−^ → H_2_O_2_ → ·OH) for conversion, thereby achieving the efficient and directed generation of ·OH.^57^ Zhang *et al.* utilized high-connectivity octanuclear aluminum clusters (Al_8_-8c) as secondary building units and covalently assembled them with linear organic linkers, successfully constructing a three-dimensional metal cluster-based COF (AICCOF-1). This strategy firmly integrates the metal clusters into the COF through covalent bonds, significantly enhancing the structural stability and functional diversity of the material. The core of the aluminum clusters consists of interconnected AlO_4_N_2_ octahedra, which exhibit properties similar to those of oxide semiconductors, introducing abundant Lewis acid sites that effectively optimize the adsorption of O_2_ molecules on the catalyst surface (with an adsorption energy of −23.1 kcal mol^−1^). This also provides key active sites for photocatalytic reactions, significantly enhancing the generation efficiency and pathway selectivity of ·O_2_^−^. In photocatalytic H_2_O_2_ synthesis, AICCOF-1 achieved a remarkable yield of up to 16 794.69 µmol g^−1^ h^−1^. Theoretical calculations further revealed that after light excitation, e^−^ migrate directionally from the aluminum clusters to the organic linkers, forming a spatially separated charge distribution that significantly enhances the electron–hole separation efficiency (Fig. 4f and g).^58^

### 3.5 Pore structure and mass transfer effects

The inherent high specific surface area and pore structure of COFs are crucial foundations for their effectiveness as catalysts.^59,60^ Nonetheless, conventional COFs are predominantly microporous (pore diameter <2 nm), which can restrict the diffusion of reactants and products during catalytic reactions that involve gas-phase product formation or require rapid mass transfer, hindering the effective utilization of internal active sites.^61,62^ Constructing COFs with hierarchical pore structures (*i.e.*, coexistence of micropores, mesopores, and macropores) is an effective strategy to address this challenge and achieve breakthroughs in photocatalytic performance. In a hierarchical pore system, micropores primarily provide a high specific surface area and abundant active sites, while mesopores and macropores act as “mass transfer channels,” significantly reducing diffusion resistance and ensuring rapid transport of reactants and products, while also facilitating the penetration and distribution of light within the material.^63,64^

**Fig. 5:**
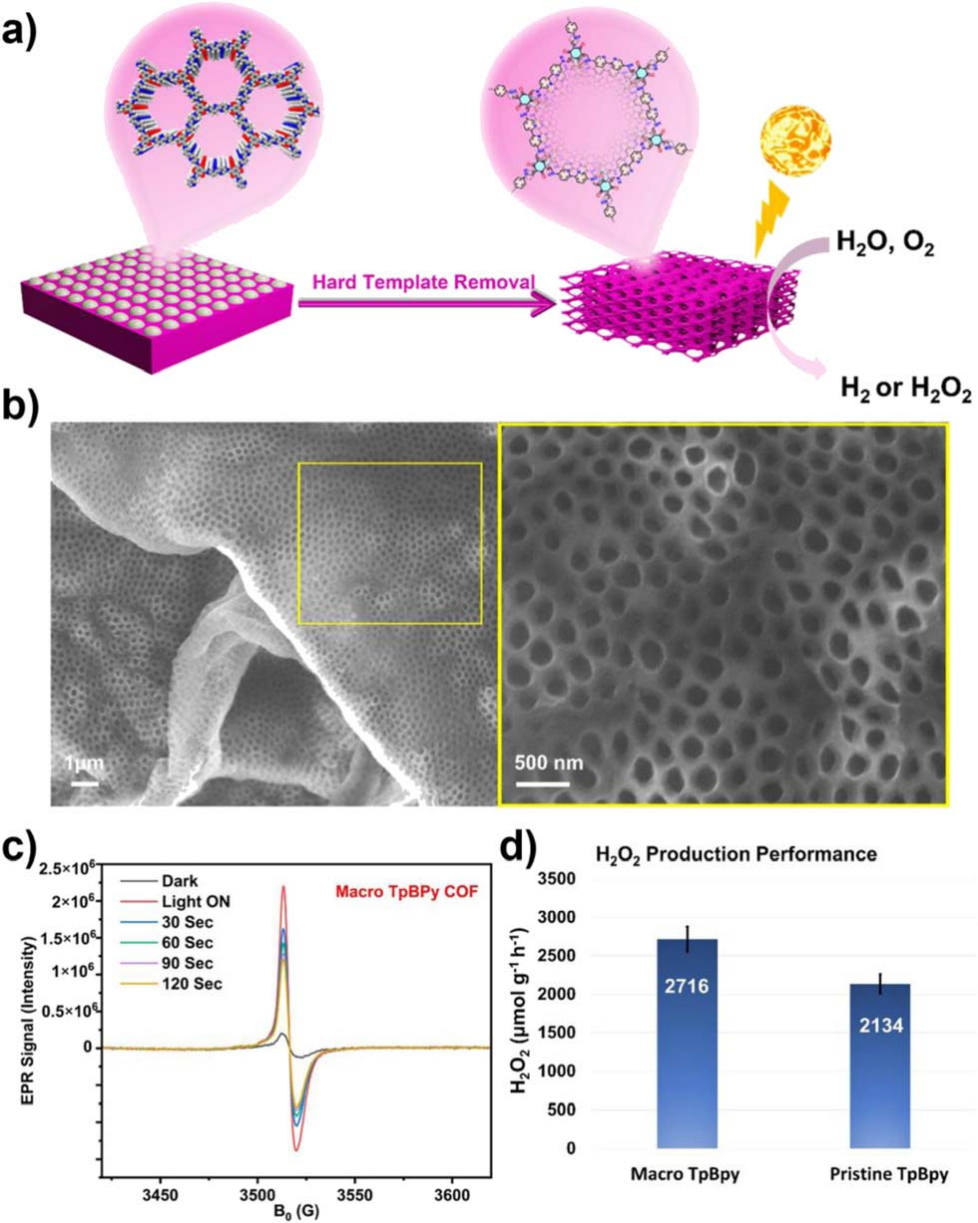
(a) Synthesis of macro-TpBpy using the PS hard template method and its photocatalytic mechanism; (b) FESEM image of macro-TpBpy; (c) EPR CB electron spectra of macro-TpBpy and original TpBpy under dark and visible light irradiation; (d) photocatalytic H_2_O_2_ performance of macro- and original TpBpy COFs. Reproduced with permission from ref. 65, Copyright 2024, from Author(s).

Khalil and colleagues' study offers compelling evidence for this. They successfully synthesized a hierarchical porous COF with a macroporous structure (macro-TpBpy) using the polystyrene (PS) hard template method and systematically compared it with the original COF (TpBpy), which has only a microporous structure (Fig. 5). Structural characterization indicates that macro-TpBpy successfully introduces interconnected macropores of approximately 270 nm while perfectly retaining its crystalline structure and microporous characteristics. This structural advantage directly translates to superior physicochemical properties: its BET surface area (1384 m^2^ g^−1^) and total pore volume (0.799 cm^3^ g^−1^) are significantly higher than those of the original TpBpy (859 m^2^ g^−1^, 0.468 cm^3^ g^−1^), demonstrating that the presence of macropores greatly enhances the accessibility of the micropore channels. In the photocatalytic production of H_2_O_2_, its yield (2716 µmol g^−1^ h^−1^) is significantly higher than that of the original COF with only micropores (2134 µmol g^−1^ h^−1^). Mechanistic analysis indicates that the hierarchical pore structure facilitates more efficient charge separation and the generation of ROS (·O_2_^−^ and ·OH). This performance enhancement arises from the synergistic effects of the hierarchical pores: micropores provide a high surface area and abundant active sites, while macropores serve as efficient transport channels, significantly optimizing the mass transfer process, ensuring the rapid diffusion of reactants and products, enhancing the penetration and utilization of light within the material, promoting the separation of photogenerated charge carriers, and inhibiting the recombination of e^−^ and h^+^, thus overall enhancing the efficiency of the photocatalytic oxidation reaction.^65^

In summary, constructing a hierarchical pore structure is a key dimension for optimizing the photocatalytic performance of COFs. This strategy significantly enhances the generation capacity of ROS and the efficiency of photocatalytic oxidation by synergistically improving mass transfer, light absorption, and charge separation behaviors. This indicates that, in addition to regulating the electronic structure of COFs, systematic design of their mesoscopic pore structures is equally crucial, thus laying a solid structural foundation for achieving efficient mass transfer and energy conversion within the “structure–ROS–substrate” paradigm.

Collectively, these design strategies indicate that optimizing photocatalysis in COFs requires integrating initial structural design with the ultimate reaction outcomes. While traditional theoretical frameworks have laid the foundation for subsequent studies, they typically attend only to specific segments of the photocatalytic cascade. Band-gap theory imposes thermodynamic constraints on the initial stage of the reaction, predicting the feasibility, in terms of redox potentials, of charge excitation in the photocatalyst and ROS generation. In contrast, active-site theory concentrates on predictive analyses of substrate adsorption, bond formation and cleavage, and the local chemical environment of the catalyst during the reaction, typically *via* sophisticated theoretical calculations.^19,20^ However, photocatalytic oxidation is a dynamic cascade that involves photon absorption, exciton dissociation, charge migration, interfacial ROS generation, and ultimately substrate conversion or product formation.^25^ A singular reliance on band-gap theory or active-site analysis may overlook critical interactions and kinetic requirements among these steps, leading to an incomplete understanding of the overall photocatalytic process; consequently, even materials with ideal band positions or abundant active sites can exhibit poor activity due to inefficient charge dynamics or ineffective ROS pathways.

The “structure–ROS–substrate” paradigm overcomes this limitation. It integrates the above dimensions into a coherent causal chain: by tuning COF linkage chemistry, energy-level engineering, charge delocalization, functional-group modifications, and the incorporation of metal nodes, it determines the types, yields, and lifetimes of ROS, which in turn dictate the efficiency and selectivity of substrate conversion. This paradigm goes beyond merely predicting thermodynamic feasibility and identifying reaction active sites; it is committed to elucidating the dynamic causal relationships operating throughout the photocatalytic process. It explicitly links molecular-level structural design to the dynamic evolution of ROS and the eventual evolution of substrates, thereby providing a unified analytical perspective for complex photocatalytic oxidation and guiding the rational design of COF materials to achieve specific oxidative transformations.

## 4 Applications in photocatalytic oxidation

The application of COFs in photocatalytic oxidation is mainly reflected in their wide range of fields from synthetic chemistry to environmental and biomedical engineering.^66,67^ Guided by the “structure–ROS–substrate” paradigm, precise control over the generation of ROS, including ^1^O_2_, ·O_2_^−^, and H_2_O_2_, can be achieved through strategic designs such as bandgap engineering, D–A unit design, metal integration, and pore environment optimization in COFs. This regulation further determines the efficiency and selectivity of the oxidation process. To provide a coherent narrative and underscore the versatility of COFs, this section is organized by major application domains, each serving as a case study for the governing principles of the “structure–ROS–substrate” paradigm. This section systematically reviews these advances, first discussing the selective oxidation of organic molecules (such as sulfides, amines, and alcohols) for the synthesis of fine chemicals, and then highlighting the sustainable production of H_2_O_2_*via* water oxidation pathways. Subsequently, the field of environmental remediation is discussed, elucidating how COF-driven ROS mineralize persistent pollutants such as dyes, antibiotics, and phenols. Finally, the unique potential of COFs in biomedical applications is explored, with a focus on their roles in ROS-mediated antibacterial activity and PDT. Their programmable structures offer the possibility of achieving synergistic and targeted therapeutic outcomes. In summary, these applications highlight the tremendous potential of COFs as multifunctional platforms in advancing green oxidation reactions.

### 4.1 Selective oxidation of organic molecules

COFs are promising photocatalysts for selective organic oxidation due to their tunable structures, strong visible-light absorption, and efficient charge carrier dynamics.^68^ This potential is rooted in their designable architectures, which enable precise modulation of the types, yields, and lifetimes of ROS. By band gap engineering, D–A design, π-conjugation control, heavy atom effects, polarization fields, active site construction, and pore optimization, COFs can precisely regulate ROS such as ^1^O_2_ and ·O_2_^−^.^69^ This structure–ROS–substrate control enables efficient, mild, and selective oxidation reactions, advancing green synthesis. This section reviews recent progress in COF-catalyzed sulfide oxidation, amine coupling, alcohol-to-carbonyl conversion, C–H activation, and biomass valorization, highlighting links between structural design and catalytic performance.

#### 4.1.1 Selective oxidation of thioethers

The selective oxidation of sulphides to sulfoxides is a crucial step in drug synthesis, with the core challenge being the precise control of oxidation degree to prevent overoxidation that leads to sulfone byproducts. COFs, through their unique structural design, have successfully achieved efficient and highly selective catalysis of this reaction, guided by band gap engineering and ROS regulation strategies. In 2021, the group led by Lang synthesized a fully conjugated triazine COF (TP-COF) with C=C connections *via* Knoevenagel condensation. Its narrow band gap (2.39 eV) significantly enhanced blue light absorption. Additionally, its larger surface area about 680 m^2^ g^−1^ provided abundant active sites. Through a unique dual-path mechanism, the TP-COF achieved an 85% conversion rate of phenyl methyl sulfide in 15 minutes under blue light, exhibiting excellent sulfoxide selectivity (99%), with its activity being three times that of the traditional catalyst g-C_3_N_4_.^70^ In 2024, the group further realized precise regulation of COFs' band gap structure and polarization properties through the topological conversion from imines to thiazoles. They designed thiazole-connected isomeric COFs (COF-TZ-1/COF-TZ-2), where COF-TZ-2, due to its larger dipole moment (3.02 debye), significantly improved charge separation efficiency, enabling the high-efficiency selective oxidation of organic sulphides under blue light/O_2_ conditions, with a 99% oxidation conversion rate of phenyl methyl sulfide within 15 min. EPR confirmed that the ·O_2_^−^ radical dominated the reaction. By optimizing the conjugated structure, the material's light absorption and reaction kinetics were enhanced.^71^

To address the low crystallinity issue of imine-based COFs, the group in 2025 prepared a fully conjugated benzodithiophene COF (BDTT-sp^2^c-COF). Its fully conjugated structure significantly improved 974 m^2^ g^−1^ surface area, charge carrier separation efficiency, and electron transfer ability. The high dipole moment (2.87 D) optimized O_2_ activation, efficiently catalyzing the sulfoxidation reaction under blue light. Specifically, in 25 min, it achieved a 95% conversion rate and >96% selectivity in oxidizing organic sulfides to sulfoxides, with excellent cycling stability, providing a new paradigm for the application of conjugated COFs in photocatalytic organic synthesis.^72^

**Fig. 6:**
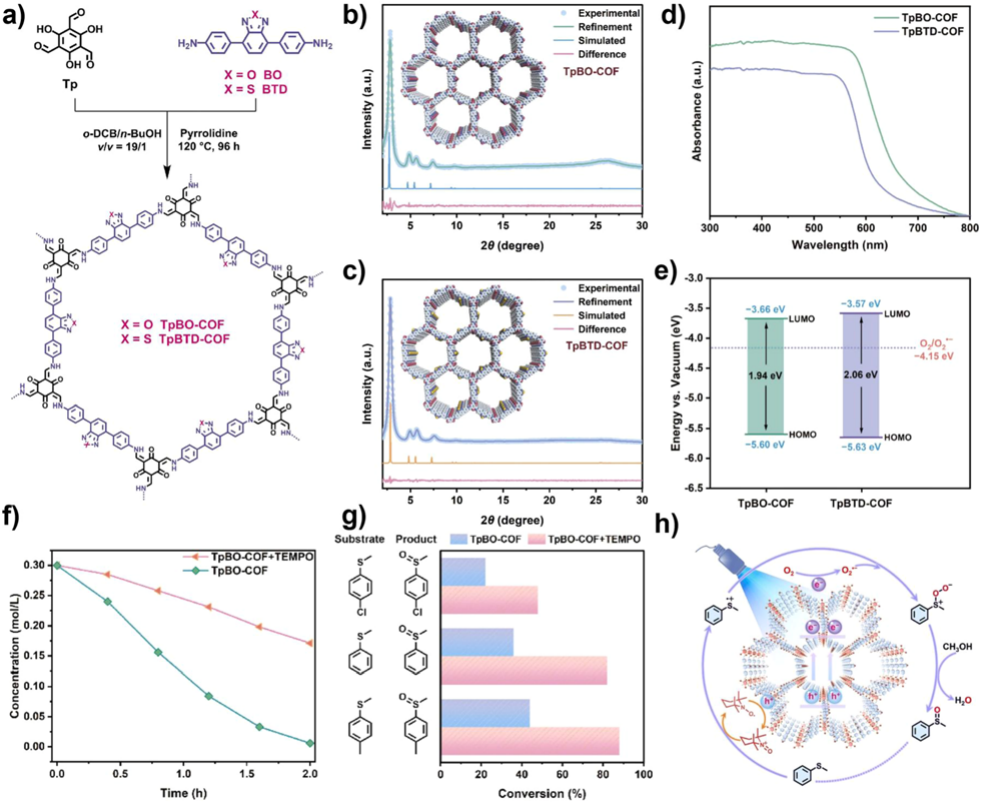
(a) Synthesis routes of TpBO-COF and TpBTD-COF; (b) experimental and simulated XRD patterns of TpBO-COF and (c) TpBTD-COF; (d) UV-Vis DRS spectra of TpBO-COF and TpBTD-COF; (e) band structures of TpBO-COF and TpBTD-COF; (f) kinetic curves of the photocatalytic oxidation of methyl phenyl sulfide on TpBO-COF with or without 3% TEMPO; (g) photocatalytic oxidation performance of methyl phenyl sulfides with three different substituents; (h) mechanism of TpBO-COF in conjunction with TEMPO in the oxidation of methyl phenyl sulfide. Reproduced with permission from ref. 76, Copyright 2024, from Elsevier.

Further enhancement of ^1^O_2_ generation efficiency is another effective pathway for improving selective oxidation of sulfides. Introducing heavy atoms into the conjugated framework helps accelerate the ISC process. Benzoselenadiazole, a unit capable of rapid photoinduced charge transfer, is widely used in photocatalysis. In 2024, Dong's group developed a selenium-containing benzoselenadiazole COF (BSe-COF), which utilized the heavy atom effect of selenium to enhance the material's spin–orbit coupling constant (SOC = 2.15 cm^−1^), promoting the ISC process and thus increasing the ^1^O_2_ yield. Under blue light irradiation, a 95% conversion rate for sulfides was achieved. More importantly, its narrow band gap (1.65 eV) extended the light absorption range to the near-infrared region, allowing it to achieve up to 96% sulfoxide yield under natural light, providing a practical solution for green, low-energy photocatalytic oxidation.^73^ 2,2,6,6-Tetramethylpiperidine-1-oxyl (TEMPO) radicals, as highly efficient reactive oxidation mediators, are widely used in organic transformations due to their high selectivity and mild reaction conditions. In 2021, Lang's group overcame the limitations of traditional homogeneous catalysis by constructing a heterogeneous TEMPO and porous porphyrin-based COF (Por-COF) system. Under white light and using O_2_ as the oxidant, this system efficiently catalyzed the selective oxidation of sulfides, with the electron transfer mechanism mediated by TEMPO improving the reaction efficiency to a >90% conversion rate.^74^ Additionally, in 2023, the team further explored the synergy between TEMPO and a benzene-selenadiazole COF (TpBSe-COF) achieving a 79% oxidation conversion rate for methyl phenyl sulfide under blue light irradiation, and realizing high selectivity (>94%) for 15 types of sulfides. The study revealed the synergy mechanism between Se atoms enhancing π–π stacking to promote charge separation and TEMPO-mediated hole transfer to facilitate ·O_2_^−^ generation.^75^ By incorporating appropriate heteroatoms into COFs to achieve heteroatom substitution, the internal electronic environment of COFs can be altered. In 2024, the team led by Lang designed a benzoxadiazole-based COF (TpBO-COF) using an oxygen atom substitution strategy (Fig. 6). The substitution with oxygen atoms broadened the visible light absorption and enhanced its charge transport properties. This material, in synergy with the TEMPO hole transfer medium, achieved efficient and selective oxidation of sulfides to sulfoxides under blue light irradiation, with a conversion rate of up to 82% (for methyl phenyl sulfide), more than double that of the thiazole-based COF (TpBTD-COF). Further studies demonstrated that the system exhibited good applicability for three methyl phenyl sulfides with different substituents. Mechanistic investigations revealed that in the synergistic catalytic system constructed by TpBO-COF and TEMPO, photogenerated e^−^ reduce O_2_ to generate ·O_2_^−^, while h^+^ oxidize TEMPO to drive the conversion of sulfides into sulfur radical intermediates, ultimately achieving efficient and highly selective oxidation of sulfides to sulfoxides.^76^

Understanding the pore size control of COFs for substrate matching is crucial for improving photocatalytic efficiency. In 2025, Shan's team designed two different pore sizes of two-dimensional porphyrin-based COFs (ETBA–por COF and ETBC–por COF) to study the influence of pore size on photocatalytic organic transformations. The study showed that the small pore-sized ETBA–por COF, with a 2367.9 m^2^ g^−1^ high surface area and fast electron transfer capability, achieved a 94% conversion rate and >99% selectivity in sulfide oxidation. The larger pore-sized ETBC–por COF, due to avoiding product blockage in the pores, achieved a 99% conversion rate and 96% yield in amine oxidation coupling. The work also used molecular simulations to confirm that pore size matching with the substrate/product size is key to enhancing photocatalytic efficiency, offering a new paradigm for designing efficient porous photocatalysts.^77^ The examples above demonstrate that precise control over the pore environment, electronic structure, and heavy-atom effects in COFs is pivotal for optimizing sulfide oxidation. This principle of structure-driven selectivity is equally critical, yet often requires different strategic emphases, for another class of vital organic transformations: the oxidative coupling of amines to imines.

#### 4.1.2 Synthesis of imines through the oxidative coupling of amines

**Fig. 7:**
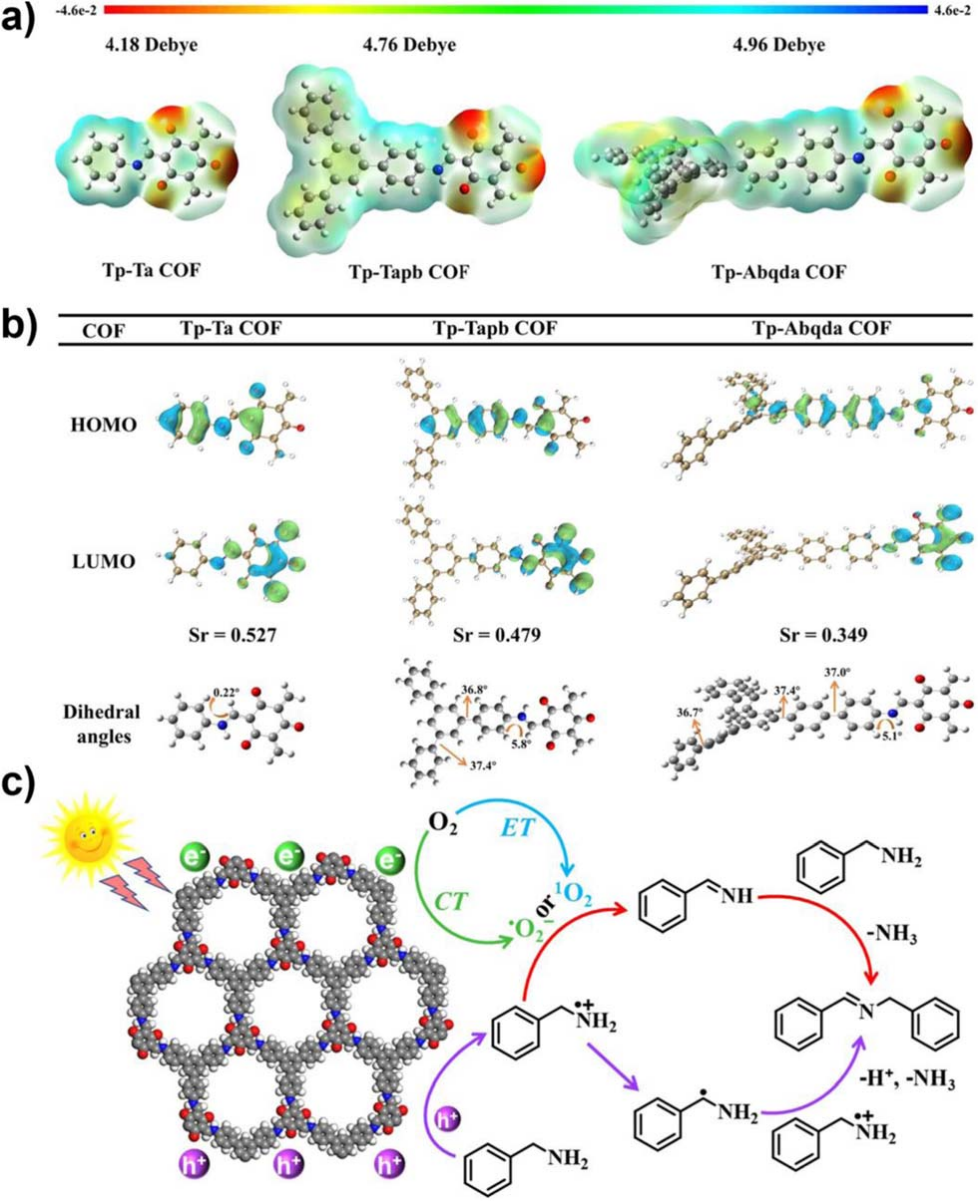
(a) Electrostatic potential (ESP) of Tp–Ta COF, Tp–Tapb COF, and Tp–Abqda COF; (b) HOMO, LUMO, and dihedral angles (isosurface value 0.03) of the three COFs; (c) mechanism diagram of the photocatalytic oxidation of benzylamine by Tp–Tapb COF. Reproduced with permission from ref. 82, Copyright 2025, from American Chemical Society.

Selective photooxidative coupling of amines represents a promising route for the synthesis of pharmaceutical intermediates and fine chemicals. COF-based photocatalysts, with tunable D–A structures, dimensional control, and metal incorporation, efficiently promote oxidative amine coupling to imines with high selectivity, while extending light absorption into the visible-NIR range enhances solar utilization and sustainability. In 2020, Lang and co-workers constructed a porphyrin-based sp^2^-carbon-conjugated COF (Por–sp^2^c-COF). Owing to the extended π-conjugation of the porphyrin macrocycle, this framework efficiently captured red light with 623 nm. In cooperation with the co-catalyst TEMPO, the key reactive species ·O_2_^−^ enabled 84% conversion of benzylamine within 12 min, with broad substrate tolerance. Its crystalline, ordered structure facilitated charge separation, while the stable –C=C– linkages effectively overcame the degradation issues common in imine-linked COFs under amine-rich conditions.^78^ Building on this, in 2022, the same group designed an oxime-linked two-dimensional porphyrin COF (Por–DETH-COF). The introduction of oxime linkages significantly enhanced the framework's resistance to amine exchange, while the ordered channels further promoted charge separation. Under red-light irradiation, Por–DETH-COF realized highly selective aerobic oxidation of amines to imines, achieving >90% conversion and >99% selectivity, together with excellent recyclability.^79^ To further enhance efficiency, integrating donor and acceptor units into COFs has become a widely explored approach to mitigate fast charge recombination. In 2022, the same group developed an innovative “monomer pre-embedding” strategy, successfully incorporating the strongly electron-deficient thiazolo[5,4-*d*]thiazole (TzTz) unit into a highly crystalline and stable β-ketoenamine-linked framework, affording a robust D–A type COF (TpDTz-COF). This material exhibited markedly enhanced visible-light absorption and efficient charge separation and migration. Under blue-light irradiation, TpDTz-COF catalyzed the selective oxidation of benzylamines and heterocyclic amines to imines with 92% conversion and 99% selectivity, outperforming analogous COFs without TzTz units. The catalyst also showed good recyclability and broad substrate scope. Mechanistic studies confirmed that the reaction followed an electron-transfer pathway, with ·O_2_^−^ as the key ROS.^80^ In 2024, Wang *et al.* developed a pyrene–porphyrin D–A COF (Py–Por-COF), constructing nanoscale heterojunctions through the donor pyrene and acceptor porphyrin units. Band engineering precisely tuned the CB potential (−0.47 V *vs.* NHE), matching the O_2_/·O_2_^−^ redox potential (−0.33 V *vs.* NHE)—a crucial factor for selectively generating ·O_2_^−^. This strategy also optimized the band gap to 2.30 eV. Owing to its broad-spectrum absorption (300–700 nm) and efficient charge separation, Py–Por-COF delivered an outstanding 99% yield in amine oxidative coupling under blue light, with an apparent quantum yield (AQY) of 11.3%. The activity remained above 90% even after four catalytic cycles.^81^ Wen's group, by varying the number of donor benzene rings in aniline, designed three D–A COFs with Tp as the acceptor and, for the first time, elucidated a polarization–planarity synergistic mechanism (Fig. 7). Tp–Tapb COF, featuring the optimal combination of dipole moment (4.76 D) and planarity (dihedral angles: 5.8°, 36.8°), achieved highly efficient photocatalytic oxidation of amines. DFT calculations confirm that the larger dipole moment of Tp–Tapb COF leads to a stronger internal electric field, thereby facilitating charge separation and transport, while moderate planarity maintains the π-conjugated structure, thus reducing energy loss. This synergistic effect enhances the production efficiency of ROS (^1^O_2_/·O_2_^−^). Therefore, the Tp–Tapb type COF material achieved a 98% aniline conversion rate within 4.5 h, outperforming similar materials and demonstrating excellent cycle stability. This study established a new paradigm for tuning linear D–A COFs through polarization–planarity synergy.^82^

Tuning the dimensionality of COFs is also vital for enhancing photocatalytic activity. One-dimensional (1D) COFs, extending along a single direction through covalent bonds combined with π–π stacking and hydrogen bonding, can achieve highly dispersed active sites and significantly improve their utilization. However, due to the anisotropy of organic chains and entropy-driven disordered packing, their synthesis remains challenging. In 2023, Zhao and co-workers developed a 1D COF (JNM-12) based on spirobifluorene-BODIPY units. With a narrow band gap and suitable energy levels, JNM-12 efficiently harvested visible light and activated O_2_ to generate both ·O_2_^−^ and ^1^O_2_, driving diverse oxidation reactions. Without metal co-catalysts, it achieved 97% conversion in benzylamine oxidative coupling and up to 68% yield in enamine aerobic oxidation under visible light, breaking the bottleneck of 1D COF synthesis and providing new insights for developing efficient metal-free photocatalysts.^83^

Furthermore, introducing appropriate metal centres can not only accelerate charge separation but also provide abundant active sites, thereby enhancing the selectivity of amine oxidative coupling. In 2024, Duan's team synthesized a bimetallic porphyrin-based COF (COF-Sr_2_Fe_1_) by coordinating Sr^2+^ and Fe^2+^ into the porphyrin centres of COF-366 *via* a two-step process. Under visible light, COF-Sr_2_Fe_1_ efficiently catalyzed benzylamine oxidative coupling with a yield as high as 97%, far surpassing single-metal and metal-free controls. The synergistic effect of the two metals not only enhanced visible-light absorption but also promoted charge separation. Importantly, DFT calculations revealed for the first time a precise division of labor: Fe sites dominated the dehydrogenation step, while Sr sites facilitated the C–N coupling step. This work provides a rational strategy for designing bimetallic active centres in COFs, and deepens mechanistic understanding of amine oxidative coupling reactions at the molecular level.^84^ Moving from nitrogen-containing to oxygen-containing compounds, the selective oxidation of alcohols poses distinct challenges, primarily centred on suppressing over-oxidation to carboxylic acids. Here, COF design strategies shift focusses towards precise exciton and polarization engineering to govern the oxidation pathway.

#### 4.1.3 Selective oxidation of alcohols to aldehydes/ketones

**Fig. 8:**
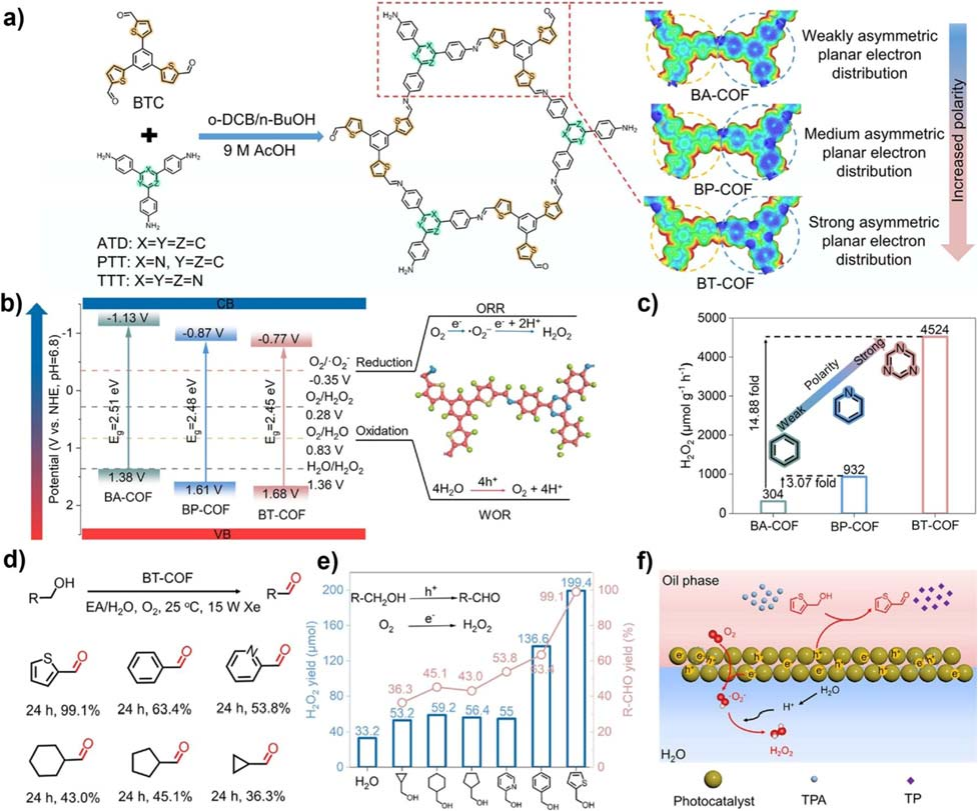
(a) Synthesis scheme and corresponding electrostatic potential of asymmetrically polarized COFs; (b) band structure diagrams of BA-COF, BP-COF, and BT-COF; (c) H_2_O_2_ production performance of different N-doped COFs; (d) aerobic oxidation of various alcohols catalyzed by BT-COF; (e and f) alcohol oxidation conversion and H_2_O_2_ production performance and mechanism. Reproduced with permission from ref. 85, Copyright 2025, from Wiley-VCH.

The photocatalytic selective oxidation of alcohols to aldehydes or ketones is of great significance in organic synthesis, as aldehyde and ketone derivatives are widely used in the pharmaceutical and chemical industries. COFs enable efficient and selective alcohol-to-carbonyl oxidation *via* strategies such as asymmetric polarization and exciton engineering, which promote exciton dissociation and mitigate overoxidation to acids. In 2025, Cheng and co-workers precisely designed a triazine-based COF (BT-COF) with asymmetric polarization, enabling asymmetric charge distribution both intralayer and intramolecularly, thereby constructing a strong internal electric field (Fig. 8). Nitrogen doping enhanced the electron-accepting strength, significantly reduced the exciton binding energy, and effectively promoted exciton dissociation. This strategy also induced a positive shift of the CB by 0.36 V, rendering it better aligned with the potential required for ·O_2_^−^ generation, thus enhancing the photocatalytic production yield of H_2_O_2_ to 4524 µmol g^−1^ h^−1^. Notably, in a biphasic reactor, BT-COF achieves not only highly selective oxidation of various alcohols but also simultaneous photocatalytic production of H_2_O_2_, with *in situ* separation of the products, highlighting its outstanding synergistic catalytic effect.^85^

Recently, olefin-linked COFs (OL-COFs) have attracted significant attention due to their outstanding stability and extended π-electron delocalization. Studies have shown that the photocatalytic performance of OL-COFs is closely related to their intrinsic excitonic effects. Reducing excitonic effects facilitates the generation of free e^−^, thereby enhancing photocatalytic activity. Therefore, developing strategies to regulate excitonic effects in OL-COFs is essential for improving their performance. In 2025, Peng and co-workers proposed an intramolecular exciton regulation strategy by designing an olefin-linked COF (TMO–BDA COF) to achieve precise optimization of the exciton binding energy. Specifically, the introduction of a strong electron-accepting unit, benzoxadiazole (BDA), significantly reinforced intramolecular D–A interactions. Femtosecond transient absorption spectroscopy revealed a strong correlation between charge separation dynamics and photocatalytic activity. This design substantially reduced the exciton binding energy, accelerated charge separation, and enhanced the generation of ·O_2_^−^ and ^1^O_2_. As a result, TMO–BDA COF achieved an 88% yield (aromatic alcohol oxidation–condensation–cyclization synthesis of benzimidazole) in a one-pot cascade reaction under visible light, and the reaction was scalable to the gram level. This study establishes an important new paradigm for tuning photocatalytic performance through exciton engineering.^86^

#### 4.1.4 Photocatalytic activation of C–H bonds

**Fig. 9:**
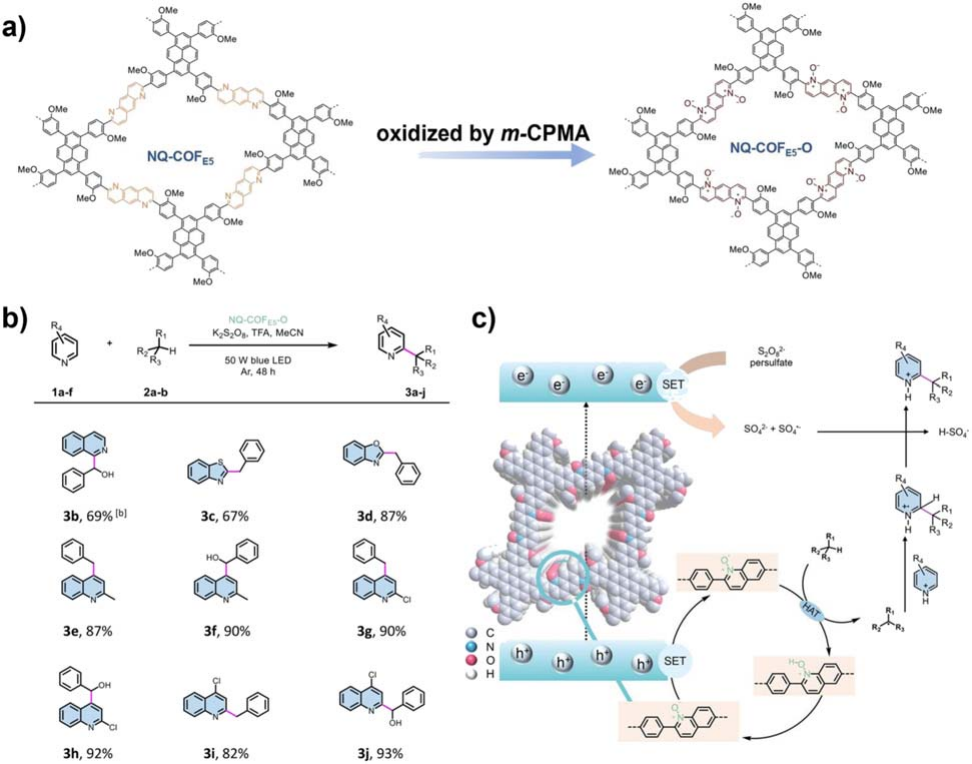
(a) Synthesis of NQ-COF_E5_-O from NQ-COFE5; (b) substrate scope of the NQ-COF_E5_-O photocatalytic C–H activation reaction; (c) mechanism of the C–H activation reaction. Reproduced with permission from ref. 87, Copyright 2023, from Wiley-VCH.

While the oxidation of functionalized substrates such as sulfides, amines, and alcohols is well-established, activating inert C–H bonds represent a frontier in synthetic chemistry. COFs offer a unique heterogeneous platform to integrate photocatalysis with hydrogen atom transfer processes, overcoming limitations of homogeneous systems. Traditional homogeneous photoredox/HAT catalyst systems, while capable of achieving site-selective C–H functionalization, are limited by inherent drawbacks such as poor cycle stability and low charge transfer efficiency. COFs, with their designable crystalline structures and modular functional integration capabilities, offer a novel approach to addressing this challenge. In 2023, Xiang and co-workers modified a non-substituted quinoline-based COF (NQ-COF_E5_) *via m*-CPBA oxidation, introducing quinoline *N*-oxide units as built-in HAT sites, thus creating the first COF (NQ-COF_E5_-O) that integrates both photosensitization and HAT catalysis (Fig. 9). This bifunctional heterogeneous photocatalyst enabled efficient functionalization of otherwise inert aliphatic C–H bonds. Across 10 different substrates such as quinoline and benzothiazole, the system achieved yields up to 93%, while retaining 90% of its activity after five cycles. The improved performance was attributed to its narrow band gap for enhanced light harvesting, improved charge separation, and the high efficiency of the embedded HAT sites in activating C–H bonds. Mechanistic studies revealed that photogenerated h^+^ oxidized quinoline *N*-oxide to generate *N*-oxyl radicals, which directly abstracted hydrogen atoms from C–H substrates, forming alkyl radicals that subsequently underwent Minisci reactions. In parallel, persulfate anions reduced by photogenerated e^−^ served as HAT agents, completing the catalytic cycle.^87^

#### 4.1.5 Conversion of aryl boronic acid to phenol

**Fig. 10:**
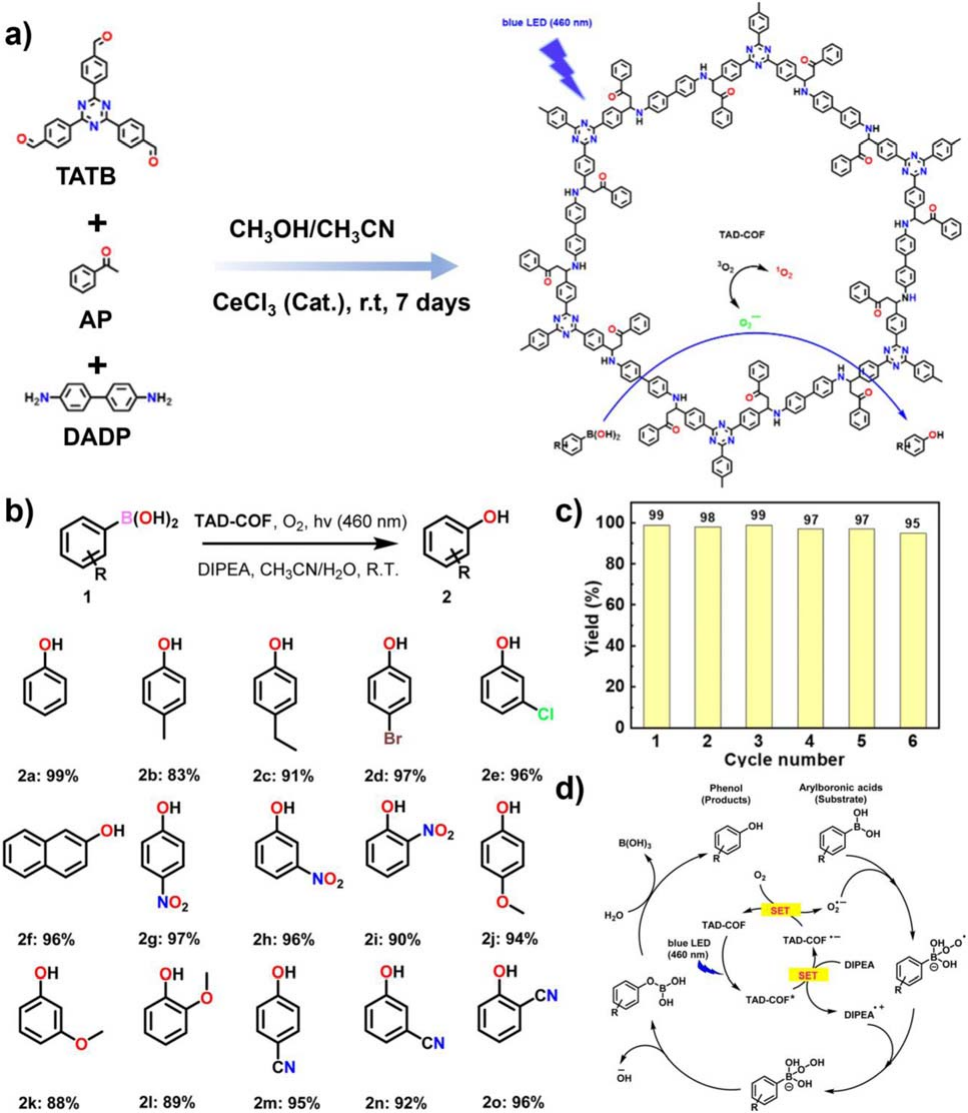
(a) Synthesis of TAD-COF through the CeCl_3_-catalyzed Mannich reaction and the mechanism of photocatalytic oxidative hydroxylation of arylboronic acids; (b) substrate scope of the TAD-COF photocatalytic oxidative hydroxylation of arylboronic acids; (c) cycle stability of the TAD-COF oxidative hydroxylation reaction of phenylboronic acid; (d) photocatalytic conversion mechanism of arylboronic acids to corresponding phenols. Reproduced with permission from ref. 90, Copyright 2024, from Author(s).

Complementary to C–H activation, the transformation of arylboronic acids to phenols showcases the ability of COFs to mediate clean oxidative hydroxylation reactions, typically *via* superoxide radical pathways under mild conditions. Photocatalytic oxidation of arylboronic acids to phenols proceeds *via* ·O_2_^−^ generation from light-excited photocatalysts, followed by radical peroxo intermediates and subsequent rearrangement or hydrolysis to afford phenols. In 2024, Zadehnazari *et al.* synthesized a tetrazine-linked vinyl COF (TA-COF-1/2) through Knoevenagel condensation. The incorporation of strongly electron-accepting tetrazine units significantly promoted ·O_2_^−^ generation, while the highly crystalline AA-stacked structure and a large surface area of 1323 m^2^ g^−1^ effectively optimized charge separation. Protonation further narrowed the band gap, thereby enhancing catalytic activity. Under blue light irradiation in air, TA-COF achieved highly efficient photocatalytic oxidation of arylboronic acids, with phenol yields of 95–99% within 3 hours, and exhibited excellent cycling stability. Importantly, its unique HCl-specific acid-responsive stability overcame the long-standing limitation of COFs under acidic conditions.^88^ To extend the light absorption range, in 2025 Chen and co-workers innovatively adopted a TfOH-catalyzed cyano trimerization strategy (metal-free), overcoming the limitations of conventional ionothermal synthesis, and developed a D–A type covalent triazine framework named NP-CTF incorporating phenoxazine and triazine units. NP-CTF displayed ultra-broad spectral absorption and a negative CB potential, enabling the simultaneous high-efficiency generation of ^1^O_2_ and ·O_2_^−^. In air, the photocatalyst achieved oxidation–hydroxylation of arylboronic acids with efficiencies of 98.2% under white LED light and 99.2% under natural sunlight, markedly outperforming TiO_2_ (63.4%) and g-C_3_N_4_ (69.1%).^89^ In 2024, Dong and colleagues employed a CeCl_3_-catalyzed Mannich reaction at room temperature to synthesize a highly crystalline β-ketoenamine-linked COF (TAD-COF) (Fig. 10). This material featured an AA-stacked structure and a narrow band gap of 1.42 eV, endowing it with excellent light-harvesting and photocatalytic activity. Under blue light, TAD-COF catalyzed arylboronic acid oxidation to phenols *via* a ·O_2_^−^ pathway, achieving 99% yield with good recyclability retaining 95% activity after six cycles.^90^

In 2022, Zhou and co-workers applied pore-wall surface engineering to an olefin-linked COF (TBT-COF) by introducing strongly electron-donating methoxy groups (–OMe), yielding MeO–TBT-COF. The strong electron-donating conjugation effect of –OMe groups significantly broadened visible light absorption, narrowed the band gap, and enhanced charge separation efficiency. Its optimized CB potential was thermodynamically favorable for activating O_2_ to ·O_2_^−^. Under air and white LED irradiation, ·O_2_^−^ nucleophilically attacked the boron centre of arylboronic acids, followed by rearrangement and hydrolysis to generate phenols with 92% yield. Compared with F- or H-modified analogues, the methoxy-functionalized COF exhibited >20% higher catalytic performance, highlighting the fine-tuning ability of pore-wall functionalization for the electronic structure and catalytic activity.^91^ In 2021, Wen's group designed a fully electron-rich pyrene-based COF (COF-JLU25) that enabled visible-light-driven hydroxylation of arylboronic acids to phenols. Under ambient air and room temperature, with only a low catalyst loading, the system achieved >99% conversion of electron-deficient arylboronic acids within 24 hours, retaining activity over seven cycles without deactivation. The superior performance was attributed to the electron-rich skeleton that promoted ·O_2_^−^ generation and the stable nanochannels that ensured efficient mass transfer. This study provides a green and practical strategy for synthesizing phenolic intermediates important in pharmaceuticals.^92^

#### 4.1.6 Biomass oxidation

**Fig. 11:**
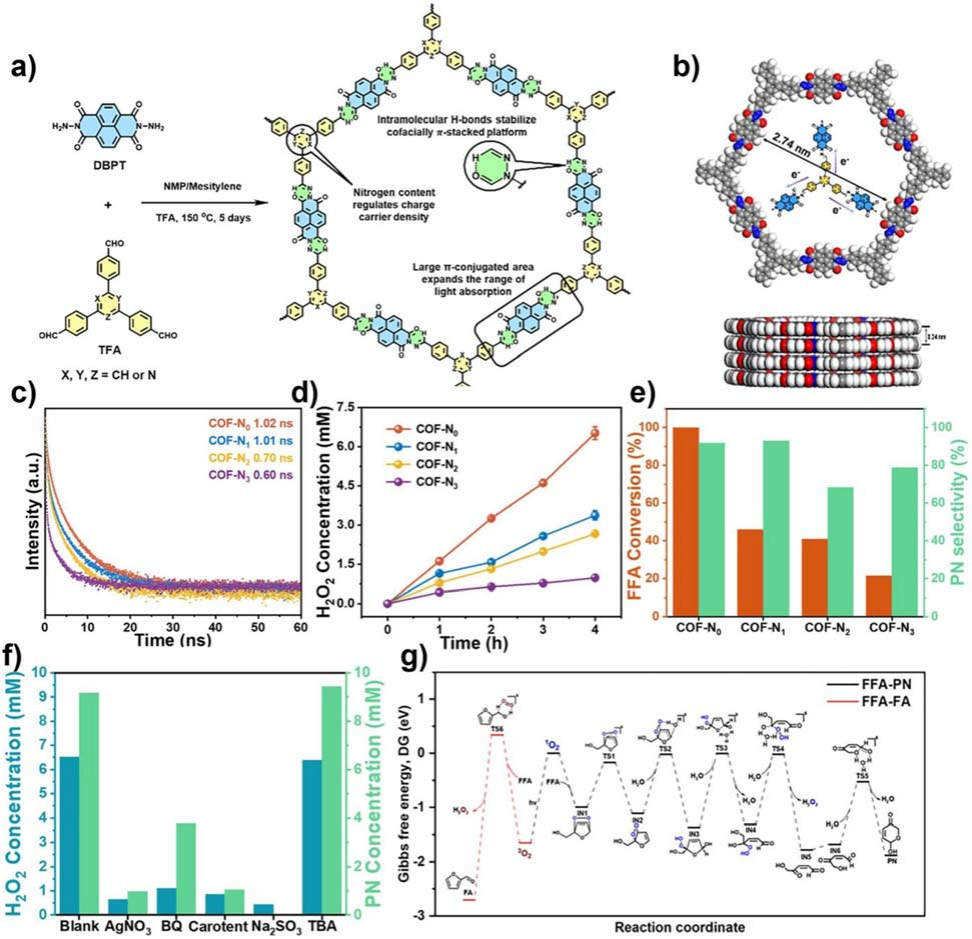
(a) Synthesis method of nitrogen-doped D–A structured COF-N_0–3_ and specific functions of different structural fragments; (b) enlarged view of the local unit including pore size and electron flow direction and the AA stacking model of COF-N_0_ observed along the *a*-axis; (c) TRPL spectrum of COF-N_0–3_; (d) photocatalytic H_2_O_2_ performance of COF-N_0–3_; (e) comparison of FFA conversion rate and PN selectivity within 4 h; (f) capture experiment of COF-N_0_; (g) DFT calculations of the reaction pathways from FFA to PN and FF. Reproduced with permission from ref. 94, Copyright 2025, from Wiley-VCH.

Beyond traditional petrochemical feedstocks, the valorisation of biomass-derived molecules into high-value chemicals is a cornerstone of sustainable chemistry. COFs enhance these transformations by integrating metal-assisted catalysis and tandem reaction designs, improving both selectivity and atom economy. Efficiently and selectively converting biomass-derived molecules into high-value-added chemicals is of great significance. COFs enhance biomass-to-chemical conversion by metal-assisted catalysis and tandem reaction design, improving selectivity and atom economy. Yu and co-workers developed a Mo-modified TPB–DMTP-COF (TPB–DMTP-COF-Mo), in which Mo clusters adsorb and activate O_2_ to generate ·O_2_^−^, while Mo^6+^ centres further oxidize to produce ^1^O_2_. This synergistic pathway efficiently drove the Achmatowicz rearrangement of furfuryl alcohol (FFA). Importantly, the selective generation of ^1^O_2_ suppressed undesired ·OH side reactions, ensuring the specificity of the Achmatowicz rearrangement and enabling the highly selective formation of pyranone—a key precursor to anti-HIV drugs. In parallel, the system co-produced H_2_O_2_ at a high rate of about 22 080 µmol g^−1^ h^−1^, significantly enhancing atom economy and overcoming the toxicity and noble-metal dependence of conventional methods. This work establishes a green paradigm for upgrading biomass-derived compounds into pharmaceutical precursors.^93^ In another example, Zhou and colleagues designed a series of polyimide-based COFs (COF-N_*x*_) with a gradient of nitrogen content. As shown in Fig. 11, by reducing the nitrogen content (COF-N_0_), they effectively optimized charge carrier dynamics (prolonged lifetime and enhanced separation), thereby promoting efficient ^1^O_2_ generation. More importantly, the constructed ^1^O_2_-driven photocatalytic system replaced conventional hole-mediated oxidation pathways, suppressing byproduct formation. Under visible-light irradiation, ^1^O_2_ selectively triggered the Achmatowicz rearrangement of FFA to yield pyranone (PN) with 99% conversion and 92% selectivity, while simultaneously generating H_2_O_2_ at about 4549 µmol g^−1^ h^−1^, achieving a solar-to-chemical conversion (SCC) efficiency of 1.73%. *In situ* DRIFTS, EPR, and DFT calculations confirmed that ^1^O_2_ initiated the rearrangement *via* a [4 + 2] cycloaddition intermediate, reducing the reaction barrier by 1.2 eV compared to the hole oxidation pathway. This avoided traditional oxidative side reactions, and the generated H_2_O_2_ was preserved without decomposition. Such a strategy transforms the conventional concept of “sacrificial agent consumption” into “high-value chemical synthesis,” providing a new paradigm for biorefining and sustainable chemical manufacturing.^94^

#### 4.1.7 Photocatalysis of other oxidation reactions

**Fig. 12:**
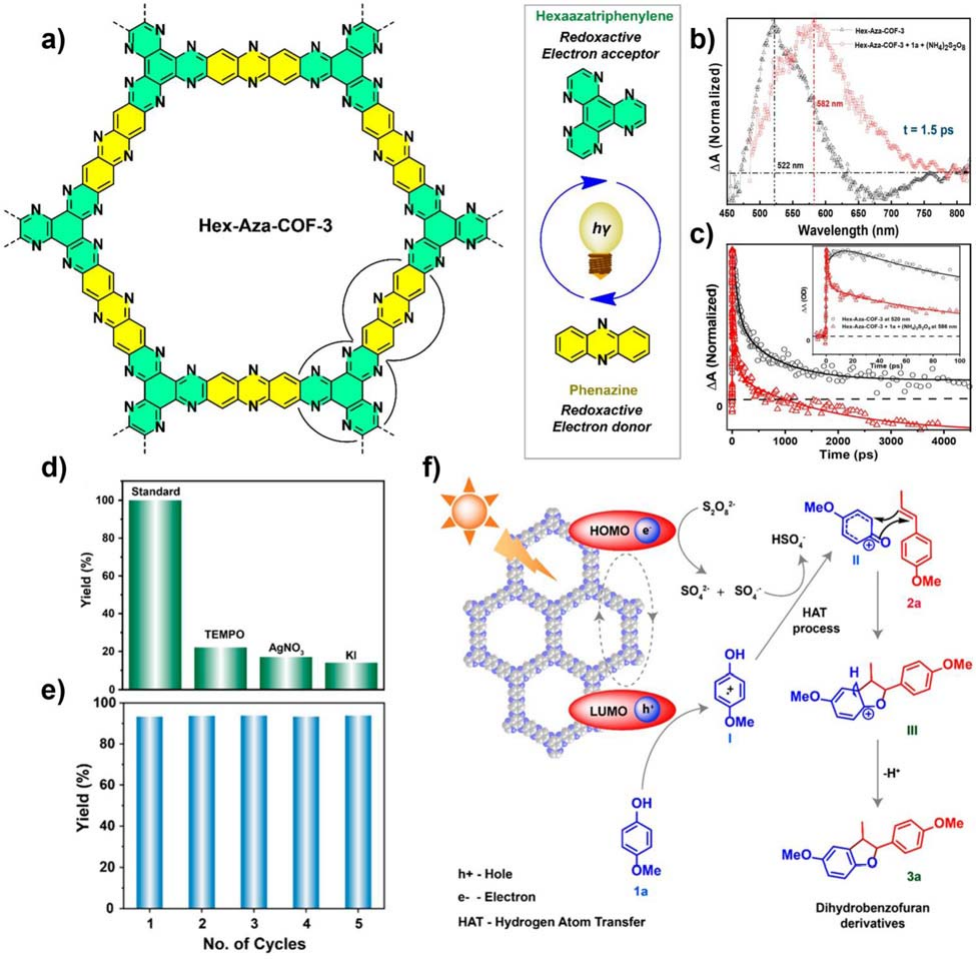
(a) Structure of Hex–Aza-COF-3; (b) transient absorption spectra of Hex–Aza-COF-3 with and without the addition of substrate 1a and oxidant (NH_4_)_2_S_2_O_8_ at a delay time of 1.5 ps; (c) kinetic traces of Hex-Aza-COF-3 at 520 nm (black line) and the mixture of Hex–Aza-COF-3 with 1a and (NH_4_)_2_S_2_O_8_ at 586 nm (red line) at late (main plot) and early (inset) time scales; (d) quenching experiments; (e) cycle stability of Hex–Aza-COF-3; (f) reaction mechanism of the Hex–Aza-COF-3 photocatalytic oxidative [3 + 2] cycloaddition reaction. Reproduced with permission from ref. 98, Copyright 2023, from Author(s).

The modularity of COFs enables their application in an even broader spectrum of oxidative transformations, continually pushing the boundaries of green synthesis and demonstrating their versatility as tunable photocatalytic platforms. Beyond the aforementioned transformations, COFs have also demonstrated remarkable potential in broader oxidation reactions, thereby continually pushing the boundaries of green synthesis. Liu and co-workers employed an isostructural COF screening strategy to design and synthesize three pyrene-based two-dimensional COFs (COF-JLU23/24/25). While maintaining identical pore structures and crystallinity, the team tuned the electronic properties of the linkage groups to optimize optoelectronic performance. Among them, COF-JLU24, featuring a D–A structure, exhibited the most outstanding visible-light photocatalytic activity. It was successfully applied to indole C-3 formylation and achieved a yield of 64% and thiocyanation with a yield of 95%, with activity far superior to traditional metal-free photocatalysts such as g-C_3_N_4_. Notably, COF-JLU24 also showed excellent substrate universality and cycling stability, retaining full activity after five runs.^95^ Niu and colleagues designed and synthesized an imidazole–porphyrin COF (PyPor-COF) with broad spectral absorption (200–700 nm). The material efficiently generated ^1^O_2_ under red light irradiation, with a quantum yield of 77.8%. PyPor-COF was applied to aqueous-phase oxidative cleavage of olefins, while exhibiting excellent tolerance toward functional groups such as halogens and cyano groups. Its superior performance was attributed to the strong red-light-harvesting capacity of the porphyrin units, the high surface area that enhanced substrate accessibility, and the selective energy transfer properties that facilitated ROS generation.^96^ Pascal Van Der Voort and co-workers adopted a multicomponent one-pot polymerization strategy to introduce sodium 4-vinylbenzenesulfonate (VBS) into a porphyrin-based COF-367 backbone, yielding a quinoline-linked Brønsted acid-functionalized COF (COF-367-SO_3_H). As the first metal-free bifunctional photocatalyst, COF-367-SO_3_H efficiently catalyzed the C–C bond oxidative cleavage of cycloketones under visible light irradiation and aerobic conditions, achieving yields as high as 83%. Mechanistic investigations revealed that the –SO_3_H groups protonated carbonyls to form enol ether intermediates, which were subsequently attacked by porphyrin-sensitized ^1^O_2_ to generate a dioxetane intermediate. The subsequent homolytic cleavage of both the O–O and C–C bonds yielded distal keto esters. This strategy effectively eliminated the reliance on noble metals or strong acids while affording excellent acid–base stability, broad substrate compatibility (*e.g.*, cyclohexanone, cyclopentanone, and cyclobutanone derivatives), and robust recyclability, thereby providing a new paradigm for the green synthesis of esters.^97^ As shown in Fig. 12, the Eddaoudi team reported Hex–Aza-COF-3, a COF constructed from rigid phenazine–hexaazatriphenylene building blocks. Under visible light irradiation, Hex–Aza-COF-3 exhibited outstanding activity for oxidative [3 + 2] cycloaddition reactions between phenols and olefins. The rigid heteroaromatic units (phenazine donors coupled with HAT acceptors) broadened the visible-light absorption window, while fully aromatized imine linkages conferred excellent stability. Transient absorption spectroscopy revealed superior charge carrier dynamics with an ultrafast charge separation time of 11.8 ps. As a result, Hex–Aza-COF-3 efficiently synthesized bioactive 2,3-dihydrobenzofuran scaffolds with yields up to 95% and was successfully applied in the late-stage synthesis of complex natural products such as (±)-conocarpan and (±)-pterocarpin. Moreover, the material retained its stability and catalytic activity over five consecutive cycles, underscoring its robustness in complex photocatalytic transformations.^98^ Having explored the diverse landscape of organic synthesis, we now shift focus to a reaction of immense industrial and environmental significance: the sustainable production of H_2_O_2_, where COFs uniquely address the challenge of coupling water oxidation with oxygen reduction.

### 4.2 H_2_O_2_ production

H_2_O_2_ is a vital green oxidant and energy carrier, yet its industrial anthraquinone synthesis is energy-intensive and unsustainable. Photocatalytic routes that generate H_2_O_2_ from O_2_ and H_2_O *via* the ORR and WOR offer an eco-friendly alternative, but are challenged by unfavorable WOR thermodynamics and H_2_O_2_ instability. Band structure tuning is thus critical for selectivity, and COFs, with adjustable electronic structures, high surface areas, and functional pores, emerge as ideal photocatalysts. In contrast to selective organic oxidations that often target a single ROS pathway, efficient H_2_O_2_ production frequently requires the synergistic coupling of multiple reaction channels, a task for which the multifunctional and tunable nature of COFs is particularly well-suited. Through rational design, COFs can regulate active sites, charge behavior, and mass transport, advancing ORR and especially WOR pathways for efficient H_2_O_2_ production.

**Fig. 13:**
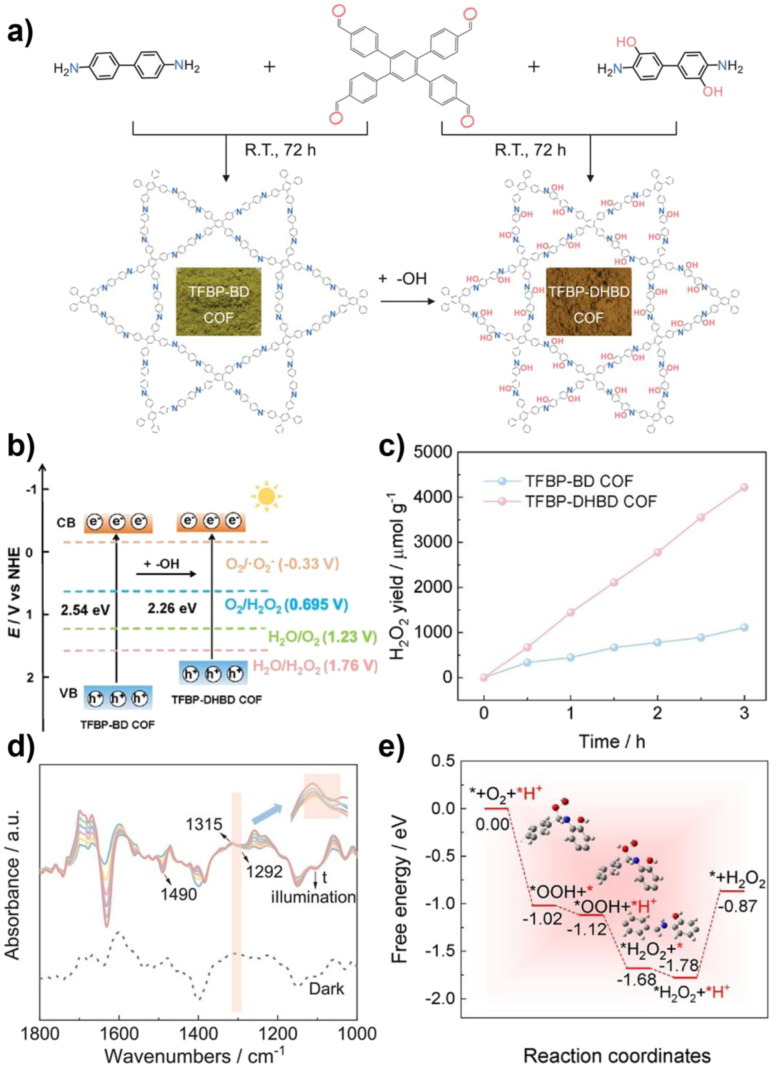
(a) Schematic diagram of the synthesis of TFBP–BD and TFBP–DHBD COFs; (b) band positions of TFBP–BD and TFBP–DHBD COFs. (c) Comparison of photocatalytic H_2_O_2_ production performance of TFBP–BD and TFBP–DHBD COFs; (d) DRIFTS spectra of TFBP–DHBD after adsorption of water and O_2_ saturation; (e) Gibbs free energy diagram for the ORR to H_2_O_2_ conversion on the TFBP–DHBD COF. Reproduced with permission from ref. 103, Copyright 2025, from Wiley-VCH.

A central challenge in photocatalytic H_2_O_2_ synthesis is activating the kinetically sluggish WOR. Research has shown that by precisely engineering active sites within COFs, this bottleneck can be effectively addressed. The photocatalytic synthesis of H_2_O_2_ mainly relies on the ORR, while the slow kinetics of the WOR is the bottleneck for the overall reaction synthesis. Research shows that by precisely regulating the active sites, the efficient WOR process can be activated in COFs. Li and co-workers regulated the number of nitrogen atoms (0–3) in heterocycles of β-ketoenamine COFs to construct biomimetic microenvironments and optimize hole utilization. Among these, the triazine-based N_3_-COF exhibited the highest hole density, with its nitrogen heterocycles functioning as dedicated WOR active centres. This design enabled a cooperative mechanism between direct 2e^−^-WOR and indirect 1e^−^-ORR pathways, ultimately delivering an impressive H_2_O_2_ production rate of 4881 µmol g^−1^ h^−1^.^99^ Similarly, Liao and colleagues synthesized three diazine-functionalized COFs (TpDz, TpMd, and TpPz) to systematically investigate the influence of nitrogen atom positioning on photocatalytic H_2_O_2_ synthesis. The pyrazine unit in TpDz stabilized endoperoxide intermediates *via* adjacent nitrogen atoms, which significantly facilitated the direct 2e^−^-ORR pathway, achieving a high H_2_O_2_ yield of 7327 µmol g^−1^ h^−1^. Importantly, the VB positions of all three COFs were sufficient to drive the WOR through the 4e^−^ pathway, oxidizing water into O_2_ while simultaneously supplying protons and e^−^—without requiring sacrificial agents. TpDz exhibited excellent charge separation efficiency and low transport resistance, further enhancing the synergy between the ORR and WOR.^100^ Liu's group developed two novel nitrogen-rich COFs (COF-JLU51 and COF-JLU52) by incorporating triazole–triazine (TTT) units. These frameworks efficiently produced H_2_O_2_ in pure water and O_2_ environments *via* a dual 2e^−^-ORR and 4e^−^-WOR mechanism. Specifically, COF-JLU51 achieved a yield of 4260.3 µmol g^−1^ h^−1^ in pure water, while COF-JLU52 demonstrated an ultra-high activity of 7624.7 µmol g^−1^ h^−1^ and an AQE of 18.2% in a benzyl alcohol/water biphasic system. *In situ* IR spectroscopy, electrochemical measurements, and DFT calculations confirmed the presence of the 4e^−^-WOR pathway, in which water molecules are oxidized at nitrogen-rich sites to produce O_2_ and protons, thereby facilitating the ORR process. The superior performance was attributed to the high surface area, efficient charge separation and transport, favorable band structures, and strong adsorption of O_2_/H_2_O at nitrogen-rich sites.^101^ Ye and co-workers designed a bipyridine-based COF (COF-TfpBpy) that utilized water as the proton source. Upon protonation at the bipyridine sites to form PyH^+^, two water molecules adsorbed as a (H_2_O)_2_ cluster, enabling a direct 2e^−^-WOR pathway to produce H_2_O_2_. This mechanism overcame the traditionally sluggish 2e^−^-WOR kinetics, achieving a WOR rate of 302 µM h^−1^ and, together with the 2e^−^-ORR pathway, nearly 100% atom utilization efficiency. At 333 K, the solar-to-chemical conversion (SCC) efficiency reached 1.08%. *In situ* IR spectra confirmed the direct 2e^−^-WOR pathway, while DFT calculations further revealed that the adsorption energy of the (H_2_O)_2_ cluster at protonated bipyridine sites was significantly more favorable than single water adsorption, thus reducing the reaction barrier substantially.^102^ Guo's group proposed a “proton reservoir” strategy by designing a hydroxyl-functionalized COF (TFBP–DHBD COF) to overcome the sluggish kinetics of the WOR in photocatalytic H_2_O_2_ synthesis (Fig. 13). Phenolic –OH groups acted as dynamic proton buffers: during the initial illumination stage, they rapidly supplied protons to the ORR to accelerate *OOH intermediate formation, while subsequently capturing delayed protons released from the WOR for cyclic utilization. This bridged the spatiotemporal mismatch between the ORR and WOR. The mechanism was validated by isotope labeling and *in situ* IR spectroscopy. Consequently, the COF achieved a H_2_O_2_ production rate of 1444.0 µmol g^−1^ h^−1^ without sacrificial agents—3.3 times higher than its non-hydroxyl analogue.^103^

Yue and colleagues challenged conventional understanding by revealing that in thiophene-based COFs (TD-COF), phenyl rings—rather than heteroatoms—served as efficient WOR active centres when linked by imine bonds to thiophene units. DFT calculations indicated that phenyl rings exhibited lower free energy barriers for *OH adsorption than conventional heteroatom sites. Coupled with a superhydrophilic design, TD-COF achieved a H_2_O_2_ production rate of 1080 µmol g^−1^ h^−1^ even under an Ar atmosphere. *In situ* DRIFTS directly detected *OOH and *OH intermediates, confirming the operation of a direct 2e^−^-WOR pathway.^104^ Chen and co-workers employed a molecular engineering strategy to design two *s*-heptazine-based COFs (HEP–TAPT-COF and HEP–TAPB-COF), enabling spatially separated dual active centres for the ORR and WOR within a single framework. Both theoretical calculations and experimental validation revealed that phenyl rings acted as efficient WOR sites, catalyzing the four-electron water oxidation to O_2_ with a negative Gibbs free energy change (Δ*G*), indicating a spontaneous process. Isotopic labeling experiments with H_2_^18^O confirmed that the generated O_2_ originated exclusively from water oxidation, which was subsequently *in situ* reduced to H_2_O_2_ by adjacent *s*-heptazine/triazine centres, forming a self-sustained oxygen supply cycle. Benefiting from the dual-ORR-centre design, HEP–TAPT-COF exhibited enhanced charge separation efficiency and accelerated WOR kinetics, achieving a H_2_O_2_ production rate of 87.50 µmol h^−1^ and a SCC efficiency of 0.65%.^105^ For further mechanistic insights, Pan and colleagues proposed a novel “hydration-triggered” WOR mechanism by synthesizing a series of hydrophilic hydrazone-linked COFs (DETH-COF and BBT-COF). In this mechanism, hydrazone linkages served as active sites where hydration directly initiated O–H bond cleavage, forming N-centred radical intermediates. Subsequently, hole-driven oxidative deprotonation and hydroxyl radical coupling selectively yielded H_2_O_2_. Experimental and theoretical studies demonstrated that O_2_ facilitated the rate-determining step by capturing protons/e^−^, reducing the energy barrier from 23 to 15.6 kcal mol^−1^. The intrinsic hydrophilicity was identified as the key factor controlling efficiency. Under visible light in pure water and ambient conditions, the optimized DETH-COF achieved a production rate of 1.0 mmol g^−1^ h^−1^, with H_2_^18^O isotopic labeling confirming that the oxygen in H_2_O_2_ originated entirely from water oxidation rather than the ORR.^106^ Tao and co-workers introduced a “framework-centred radical strategy” by employing carbonyl groups in fluorinated COFs (Kf–F-COF) as photogenerated radical sites. Through a hydrogen atom transfer (HAT) mechanism, both O_2_ and H_2_O were simultaneously activated, enabling efficient dual-channel H_2_O_2_ synthesis under visible light. Kf–F-COF produced H_2_O_2_ directly *via* the 2e^−^-WOR pathway without forming hydroxyl radical intermediates, while synergistic ORR further boosted the yield to 6.42 mmol g^−1^ h^−1^. Fluorination significantly increased the electron affinity of carbonyl groups, promoting dual radical formation and lowering energy barriers, thereby ensuring excellent stability and activity in both natural sunlight and seawater environments.^107^

**Fig. 14:**
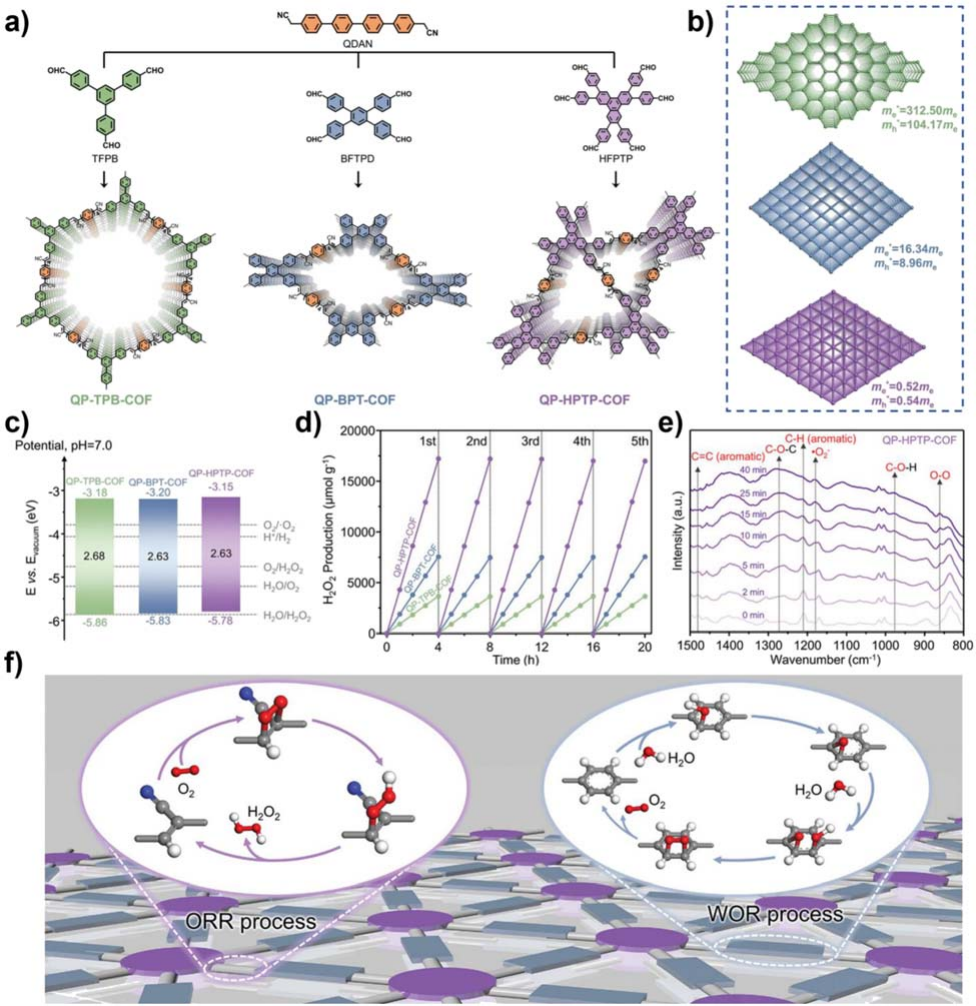
(a) Synthesis schemes of QP–TPB-COF, QP–BPT-COF, and QP–HPTP-COF; (b) schematic diagram of the relationship between the topological structures of the three COFs and their effective masses (
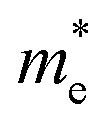
 and 
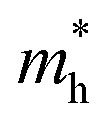
); (c) band structures of the three COFs; (d) photocatalytic H_2_O_2_ production performance (pure water and oxygen-saturated); (e) *in situ* DRIFT spectra of H_2_O_2_ generation by QP–HPTP-COF photocatalysis; (f) mechanism diagram of H_2_O_2_ generation by QP–HPTP-COF photocatalysis. Reproduced with permission from ref. 109, Copyright 2024, from Wiley-VCH.

The topology and linkage chemistry of COFs critically influence their electronic structures, hydrophilicity, and charge separation efficiency, thereby dictating WOR performance. Yang and colleagues designed COFs with distinct topologies—cpt (TBD-COF) and hcb (TBC-COF)—to probe these effects. The cpt topology endowed stronger hydrophilicity, enhancing water adsorption and proton transfer. This lowered the energy barrier for the rate-determining step from 4.09 to 2.97 eV, resulting in a H_2_O_2_ production rate of 380 µmol g^−1^ h^−1^ even under an Ar atmosphere.^108^ Xu and co-workers further advanced this concept by synthesizing three sp^2^-carbon-linked 2D COFs with distinct topologies (hcb, sql, and hxl) to systematically investigate topology-dependent photocatalytic performance (Fig. 14). Despite similar band structures and compositions, the hxl-topology QP–HPTP-COF exhibited the best catalytic activity, achieving an SCC efficiency of 1.41%. Combined spectroscopic and computational analyses revealed that the hxl topology significantly reduced exciton binding energy and carrier effective mass, thereby enhancing charge separation and transport. Notably, the WOR occurred *via* a four-electron pathway at phenyl ring sites, synergistically coupled with a two-electron ORR pathway at cyanoethylene linkage sites, completing the overall H_2_O_2_ synthesis.^109^ Building upon this, Yue and co-workers developed sub-stoichiometric [6 + 6] COFs (HHT-COF) by employing conjugation and linkage-directional strategies. The kgd topology with periodically retained aldehyde groups enhanced hydrophilicity, while mechanistic studies identified phenyl rings—rather than aldehydes—as the true WOR active centres. HHT-COF exhibited the lowest *OH formation barrier, accelerating the WOR. Coupled with the 2e^−^-ORR pathway, this COF achieved a remarkable H_2_O_2_ production rate of 4996 µmol g^−1^ h^−1^ in pure water under air.^110^ Expanding this strategy, two kgd-topology COFs (TPT-COF and TPB-COF) were synthesized. Remarkably, TPB-COF directly oxidized water *via* a one-step 2e^−^-WOR pathway to generate H_2_O_2_, maintaining 509 µmol g^−1^ h^−1^ even under Ar. When combined with efficient 2e^−^-ORR, the total H_2_O_2_ yield reached 7874 µmol g^−1^ h^−1^ in pure water and 5696 µmol g^−1^ h^−1^ in natural seawater. Both experimental and theoretical analyses demonstrated that the kgd topology effectively facilitated charge separation, reduced energy barriers for *OOH and *OH intermediates, and enhanced water adsorption and activation. Importantly, the *in situ* generated H_2_O_2_ was successfully applied to *E. coli* disinfection, highlighting its potential for real-world applications.^111^

Beyond topology engineering, altering the orientation of linkage units can yield compositionally isomeric COFs with distinct optoelectronic properties and catalytic performances. Yang and co-workers synthesized fully conjugated thiophene–pyridine-bridged COFs (TBA-COF/TCA-COF) *via* a one-pot Pictet–Spengler cyclization reaction. This cyclization precisely positioned WOR active centres at pyridine units while localizing ORR centres at triazine/phenyl sites, thereby enhancing charge separation efficiency and extending the fluorescence lifetime to 2.20 ns. The VB potential was positively shifted to 1.51 V (*vs.* NHE), thermodynamically driving the 2e^−^-WOR pathway. Coupled with the 2e^−^-ORR pathway, TBA-COF achieved a remarkable H_2_O_2_ production rate of 8878 µmol g^−1^ h^−1^ in real seawater, of which the WOR pathway independently contributed 487 µmol g^−1^ h^−1^ under Ar.^112^ Wang and colleagues synthesized imine-linked isomeric COFs named PB–PT-COF and PT–PB-COF to systematically investigate pathway-dependent differences in photocatalytic H_2_O_2_ synthesis. PB–PT-COF, with positively charged TAPB nodes, facilitated *OH intermediate formation, enhancing H_2_O adsorption and lowering the energy barrier for the WOR, thereby enabling direct water-to-H_2_O_2_ conversion. In contrast, PT–PB-COF favoured the generation of ·O_2_^−^, producing H_2_O_2_ primarily *via* the ORR pathway. Both experimental and theoretical studies confirmed that isomerization effectively regulates electronic structures and pathway selectivity.^113^ Li and co-workers proposed a hydroxyl-defect strategy, synthesizing a pyrimidine-based COF (COF-BD) with mixed imine and β-ketoenamine linkages. This architecture lowered the VB maximum (VBM = 1.62 V) and redirected the WOR pathway from 2e^−^ to 4e^−^, accelerating hole consumption and dramatically enhancing catalyst stability. COF-BD achieved a high H_2_O_2_ production rate of 5312 µmol g^−1^ h^−1^ in pure water, retaining 70% of its initial activity over 10 h, significantly outperforming β-ketoenamine-linked COF-TP.^114^

**Fig. 15:**
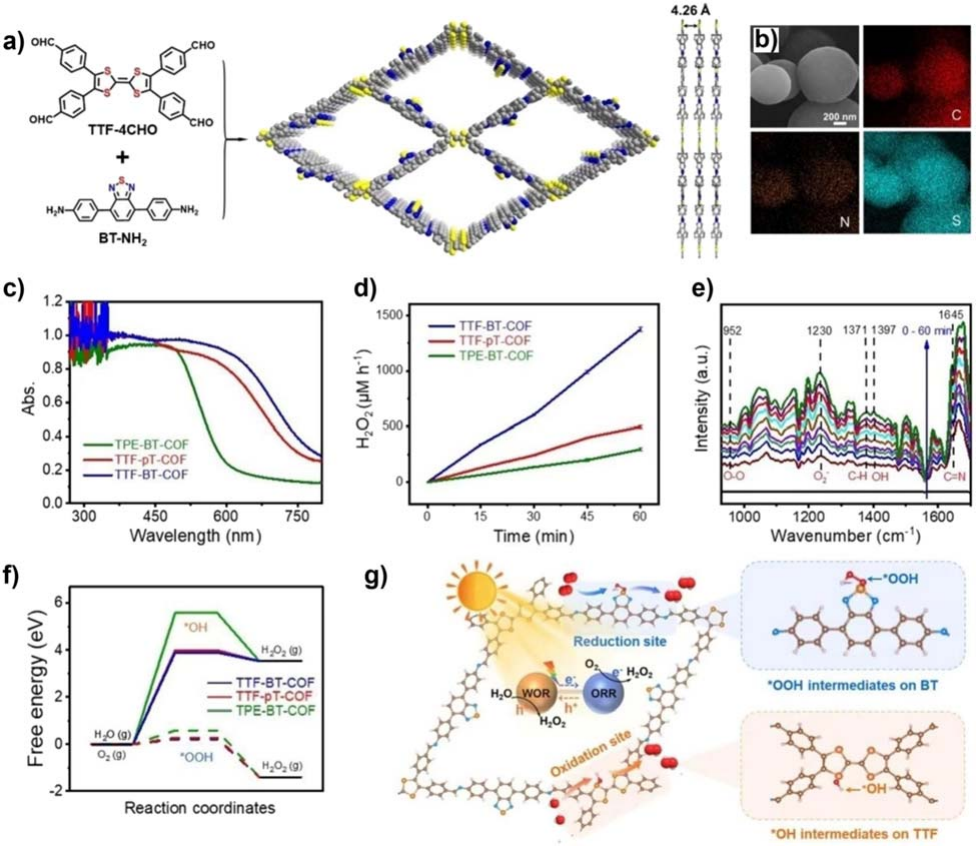
(a) Synthesis diagram of TTF–BT-COF; (b) SEM and elemental mapping images of TTF–BT-COF; (c) UV-Vis DRS spectra of TTF–BT-COF, TTF–pT-COF, and TPE–BT-COF; (d) comparison of photocatalytic H_2_O_2_ production activity of TTF–BT-COF, TTF–pT-COF, and TPE–BT-COF in pure water and O_2_ atmospheres; (e) DRIFTS spectra of TTF–BT-COF during H_2_O_2_ photosynthesis; (f) Gibbs free energy diagrams for photocatalytic H_2_O_2_ production *via* WOR (solid line) and ORR (dashed line) pathways for TTF–BT-COF, TTF–pT-COF, and TPE–BT-COF; (g) adsorption configurations of *OH intermediates on TTF and *OOH intermediates on BT in TTF–BT-COF. Reproduced with permission from ref. 119, Copyright 2022, from Wiley-VCH.

Constructing D–A frameworks is one of the most effective strategies to promote charge separation and has been widely applied to enhance H_2_O_2_ production. Jiang and co-workers designed an acetylene-bridged D–A COF (EBBT-COF), where bithienyl (BTT) donor units catalyzed the 2e^−^-WOR efficiently, as confirmed by *in situ* DRIFTS and RRDE measurements. Triethynylbenzene (TAEB) served as an electron acceptor, boosting hole transport and acting as an “electron reservoir”. The acetylene linkage enhanced π-conjugation and accelerated charge separation, delivering a WOR activity of 1563 µmol g^−1^ h^−1^ and, when coupled with the 2e^−^-ORR, a total H_2_O_2_ yield of 5686 µmol g^−1^ h^−1^ with a quantum efficiency of 15.14%.^115^ Wang and co-workers designed a cyano-functionalized COF (TBTN-COF) by integrating strong electron-accepting TBTN units with hole-rich benzotrithiophene (BTT) donors, enabling efficient sacrificial-agent-free H_2_O_2_ synthesis in pure water. Ultrafast intramolecular electron transfer (<500 fs) and long-lived charge-separated states allowed BTT to efficiently drive the WOR to generate O_2_, which then served as an *in situ* oxygen source for the ORR. *In situ* DRIFTS and DFT confirmed that cyano sites stabilized Yeager-type O_2_ adsorption, suppressing superoxide pathways and directly forming *OOH intermediates, significantly improving H_2_O_2_ selectivity. As a result, TBTN-COF achieved an impressive H_2_O_2_ production rate of 11 013 µmol g^−1^ h^−1^.^116^ Li and co-workers further constructed a triazine-based D–A COF (TPB–TPT-COF) *via* Schiff-base condensation between triazine acceptors and TPB donors, enabling sacrificial-agent-free H_2_O_2_ synthesis from water and oxygen. Under visible light, TPB–TPT-COF delivered a yield of 6740 µmol g^−1^ h^−1^, far surpassing the control without triazine units. Mechanistic studies revealed the coexistence of 2e^−^-ORR and 4e^−^-WOR pathways. Notably, the 4e^−^-WOR route was verified using AgNO_3_ scavenger tests and ^18^O isotopic labeling, confirming O_2_ evolution, while *in situ* IR and DFT identified *O–OH intermediates and active sites near imine linkages.^117^ Ma and colleagues systematically investigated D–π–A COFs with *N*-heteroaryl acceptors of varying electron-withdrawing strength (benzene < pyridine < pyrimidine < triazine). Increasing nitrogen content enhanced electron affinity, thereby promoting electron transfer from donor to acceptor across π-bridges and improving charge separation. Among them, Tf–TAPT-COF (triazine acceptor) exhibited the highest H_2_O_2_ yield (2700 µmol g^−1^ h^−1^) without sacrificial agents. Both experimental and computational analyses indicated that photogenerated h^+^ localized on donor phenyl aldehyde sites served as the main WOR centres for direct 2e^−^-water oxidation, while the π-bridge and acceptor jointly facilitated the ORR.^118^ In another research, Lan and co-workers constructed a redox-active molecular junction COF (TTF–BT-COF) by covalently linking tetrathiafulvalene (TTF, oxidation centre) with benzothiadiazole (Fig. 15). Without sacrificial agents, this framework efficiently generated H_2_O_2_*via* concurrent 2e^−^-WOR and 2e^−^-ORR pathways, achieving an extraordinary rate of 276 000 µM g^−1^ h^−1^. Mechanistically, the WOR proceeded through dehydrogenation of water at TTF sites to form ·OH intermediates, which subsequently coupled to yield H_2_O_2_ with an energy barrier of only 3.90 eV. RRDE testing ruled out competing 4e^−^ OER. Isotopic labeling (^18^O) confirmed near-100% atomic utilization of H_2_O and O_2_, while DRIFTS and EPR verified the presence of ·OH and *OOH intermediates.^119^ Ding and co-workers adopted a one-pot “grafting-to” strategy to incorporate amino-heterocycles such as 2-aminothiazole into COFs, reconstructing local electronic configurations to form intramolecular D–A structures. This design promoted efficient charge separation and optimized band positions, satisfying thermodynamic requirements for the WOR. Notably, COF-Thz synergistically combined 2e^−^-WOR and 1e^−^-ORR pathways, achieving an H_2_O_2_ yield of 3701 µmol g^−1^ h^−1^ and a solar-to-chemical conversion efficiency exceeding that of natural photosynthesis. Moreover, when applied with Fe(ii), COF-Thz exhibited nearly 100% bactericidal efficiency and >90% biofilm removal, underscoring its dual potential in photocatalysis and biomedical applications.^120^

**Fig. 16:**
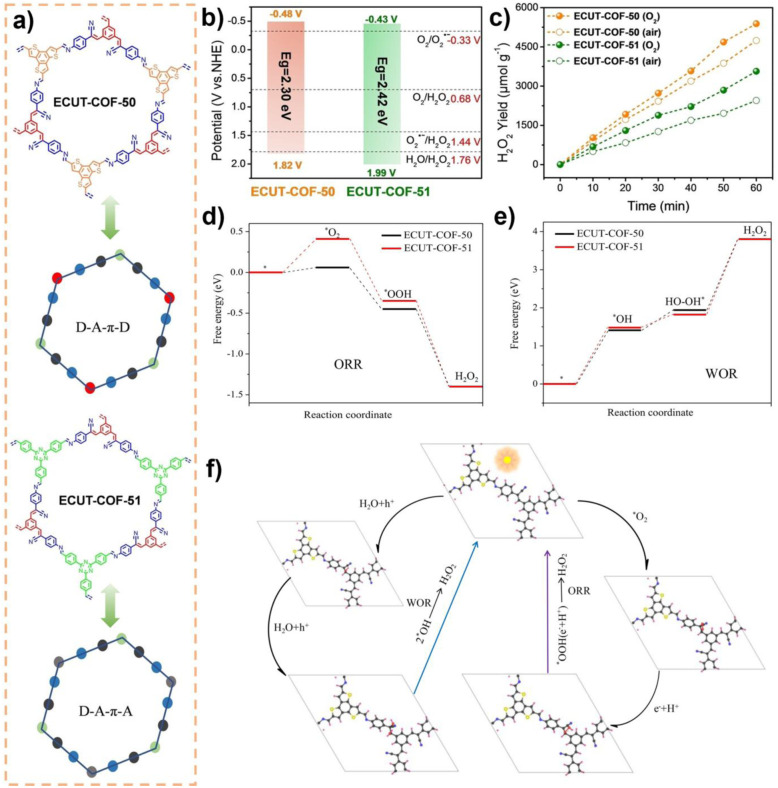
(a) Topological views of D–A systems in ECUT-COF-50 and ECUT-COF-51; (b) band positions of ECUT-COF-50 and ECUT-COF-51; (c) H_2_O_2_ production performance of ECUT-COF-50 and ECUT-COF-51 under O_2_ and air conditions; (d and e) Gibbs free energy diagrams for photocatalytic H_2_O_2_ production under ORR and WOR conditions for ECUT-COF-50 and ECUT-COF-51; (f) mechanism of photocatalytic H_2_O_2_ production from the ORR and WOR using ECUT-COF-50. Reproduced with permission from ref. 121, Copyright 2024, from Wiley-VCH.

Functional modification of COF skeletons is an effective approach to finely tune their physicochemical properties and catalytic performance. As shown in Fig. 16, Luo and co-workers designed a cyano-functionalized D–A–π–D COF (ECUT-COF-50) *via* a two-step synthesis strategy, successfully constructing a photocatalyst with a unique electronic structure. Under sacrificial agent-free conditions, ECUT-COF-50 enabled efficient H_2_O_2_ production directly from air and water, achieving a yield of 4742 µmol g^−1^ h^−1^ with an O_2_ utilization efficiency of 88%. This advance addressed the key challenge of the WOR pathway: the strong electron-withdrawing effect of cyano groups reconstructed the charge distribution of adjacent C=C bonds, turning them into efficient WOR active centres that directly oxidized H_2_O into H_2_O_2_ rather than *via* the conventional O_2_ intermediate. Both experiments and DFT calculations confirmed that the *HO–OH intermediate at the C=C site exhibited a substantially reduced adsorption energy, lowering the energy barrier of the rate-determining step compared to the control COF. Meanwhile, the VB potential satisfied the thermodynamic requirements for the WOR.^121^ Liu and colleagues employed a multicomponent reaction (MCR) to synthesize six quinoline-linked triazine COFs (R–QN–TA-COFs) with substituent-controlled properties. Among them, the methoxy-modified MeO–QN–TA-COF exhibited outstanding performance, delivering 7384 µmol per g per h H_2_O_2_ under sacrificial agent-free conditions in pure water and air. The methoxy groups enhanced the electron density and hydrophobicity of the framework, simultaneously optimizing both the ORR and WOR. Quenching experiments, where EDTA suppressed hole transfer and decreased yields, *in situ* EPR detection of ·OH intermediates, and DFT analysis showing enhanced HOMO–LUMO overlap confirmed the critical contribution of the WOR. Remarkably, MeO–QN–TA-COF still produced 1353 µmol per g per h H_2_O_2_ under a N_2_ atmosphere, verifying its intrinsic water oxidation capability.^122^ Shen and co-workers synthesized a hydroxyl-functionalized imine-linked COF (TT-COF-OH). This COF exploited triazine-ring sites to directly oxidize water into H_2_O_2_ without free radical intermediates, assisted by a strong internal electric field (IEF) that promoted exciton dissociation and optimized hydrophilic interfaces. TT-COF-OH achieved a high H_2_O_2_ yield of 3406 µmol g^−1^ h^−1^. *In situ* DRIFTS confirmed the formation of *OOH intermediates, while fs-TAS revealed that IEF-induced shallow trap states extended carrier lifetimes by sixfold, addressing the fundamental challenge of inefficient exciton dissociation in 2D materials.^123^

Shu and colleagues introduced hydroxyl groups into β-ketoenamine-linked COFs, constructing Tz-THBZ for dual-channel H_2_O_2_ production. Tz-THBZ exhibited an optimal VB position and excellent exciton dissociation capability. This design significantly enhanced WOR performance, enabling 940 µmol per g per h H_2_O_2_ yield under a N_2_ atmosphere, thus confirming its direct water oxidation capability. When coupled with efficient ORR, Tz-THBZ achieved a total yield of 4688 µmol g^−1^ h^−1^ in pure water. DFT calculations further revealed that the ketonic phenyl ring served as the oxidation centre while the triazine acted as the reduction centre, synergistically promoting dual-channel H_2_O_2_ generation.^124^

Through interfacial engineering, Zhang and co-workers synthesized a series of pyrene-based COFs (PyCOFs) with different functional groups. Among them, hydroxyl-functionalized PyCOF-OH exhibited exceptional hydrophilicity and efficient charge separation. Even under a N_2_ atmosphere, it produced 115 µmol per g per h H_2_O_2_, confirming its ability to generate H_2_O_2_*via* the WOR pathway. Mechanistic studies further revealed a ·OH-mediated two-step reaction mechanism. Coupled with efficient ORR, PyCOF-OH achieved 2961 µmol per g per h H_2_O_2_ in 25 mL pure water, while retaining excellent cycling stability.^125^ Hua and colleagues transformed a cyano-functionalized COF (PTTN-CN) into amidoxime-functionalized PTTN-AO *via* post-synthetic modification. Although H_2_O_2_ was primarily generated *via* the ORR pathway (yield 6024 µmol g^−1^ h^−1^, SCC efficiency 0.61%), the WOR still played a pivotal role, oxidizing water to O_2_*via* a 4e^−^ pathway and providing a continuous oxygen supply for the ORR, thus forming a catalytic cycle. *In situ* DRIFTS and RRDE confirmed WOR involvement and highlighted PTTN-AO's advantages in O_2_ supply and proton transfer. The amidoxime groups enhanced hydrophilicity, O_2_ adsorption, and charge separation, while lowering ORR barriers, thereby boosting overall photocatalytic activity.^126^ Shen's group further developed a thioether-functionalized triazine COF (TDB-COF). Thioether groups broadened visible-light absorption, optimized band structure, and activated a two-step 2e^−^-WOR pathway rarely achieved in COFs. EPR directly detected ·OH intermediates, while DFT confirmed that thioether sites significantly lowered the Gibbs free energy barrier for ·OH formation, accelerating WOR kinetics. In O_2_-saturated systems, TDB-COF achieved a H_2_O_2_ yield of 723.5 µmol g^−1^ h^−1^.^127^ Tang and colleagues designed partially fluorinated triazine COFs (TP/TAPT-F COFs). Fluorination not only enabled efficient H_2_O_2_ generation *via* the 2e^−^-ORR but also significantly boosted 4e^−^-WOR activity. The incorporation of fluorine optimized the electronic structure, improved crystallinity and π–π stacking, and tuned the VB to drive water oxidation to O_2_ and H^+^, thereby supplying sustainable reactants for the ORR. Isotope labeling, RRDE, and *in situ* DRIFTS unambiguously confirmed the presence and contribution of the WOR. TP/TAPT-F COFs exhibited a H_2_O_2_ yield of 1655 µmol g^−1^ h^−1^ with excellent stability, and were successfully applied in continuous-flow reactors for efficient degradation of organic pollutants.^128^

### 4.3 Environmental remediation

#### 4.3.1 Degradation of dyes

After examining the synthesis of valuable chemicals and oxidants, we turn to the critical application of COFs in environmental remediation, where their ability to generate non-selective ROS is harnessed for the degradation of persistent pollutants. Industrial wastewater often contains high concentrations of synthetic dyes, which are characterized by high chromaticity, resistance to degradation, and potential toxicity. The tunable structures of COFs make them particularly effective in addressing this challenge, as their photocatalytic activity can be tailored to break down these complex molecules. Typical organic dyes present in industrial wastewater, including rhodamine B (RhB), methylene blue (MB), and methyl orange (MO), are characterized by high chromaticity, resistance to degradation, and significant toxicity.^129^ These pollutants feature stable conjugated molecular structures and generate hazardous intermediates such as aromatic amines and free radicals during photodegradation processes, creating substantial environmental risks.^130^ COFs have emerged as promising materials for photocatalytic dye degradation due to their precisely tunable π-conjugated architectures, exceptional surface area properties, and outstanding photoelectrochemical performance.^131^ Recent advances demonstrate that strategic modifications including single-atom incorporation, D–A system engineering, band structure optimization, and heterojunction fabrication can significantly enhance the light-harvesting capability, charge separation efficiency, and ROS generation capacity of COF-based photocatalysts. These improvements ultimately lead to superior mineralization efficiency for various dye pollutants. This section provides a systematic examination of structure–performance relationships in COF-mediated degradation of representative dyes with particular emphasis on elucidating the fundamental mechanisms governing photocatalytic activity and degradation pathways.

RhB molecules feature highly conjugated eosin-type aromatic rings bridged by vinyl linkages, conferring exceptional chemical and photochemical stability and rendering them difficult to degrade rapidly in aqueous environments *via* conventional approaches.^132^ Recent COF design strategies have therefore centred on precisely tailoring band structures and energy-level alignments to optimize the generation, separation, and utilization of photogenerated charge carriers.^133^ For instance, Xiao *et al.* reported a β-ketoamine-linked, multi-segment D–A backbone that establishes a pronounced built-in electric field at the molecular scale, markedly enhancing spatial charge separation.^134^ The resulting COF possesses a surface area about 700 m^2^ g^−1^, a pore size of 2.3 nm, and visible-light absorption extending to 650 nm. Under simulated solar irradiation, it completely degrades RhB within 3 h, achieving an apparent rate constant of 1.21 h^−1^. Band structure analysis reveals that its CB is suitably positioned to drive O_2_ reduction to ·O_2_^−^, while its VB can oxidize water to ·OH. Furthermore, the wide-pore architecture facilitates rapid RhB diffusion to active sites, enabling the efficient, synergistic generation and utilization of dual-channel ROS. In another study, Wang *et al.* constructed a fully conjugated, coplanar sp^2^-carbon skeleton by condensing three distinct aromatic amine units (TPB, TEB, and TFB) with a triazine aldehyde linker.^135^ Among these, TEB-COF exhibited the highest activity, achieving complete removal of 10 mg L^−1^ RhB within 30 min under visible-light irradiation, with an apparent rate constant 2.4 times higher than that of the non-optimized TPB-COF. The photocatalyst retained its performance over five consecutive cycles without significant loss, and the total organic carbon (TOC) removal reached 78%. Active species trapping and ESR analysis revealed that both photogenerated h^+^ and ·O_2_^−^ acted synergistically to drive oxidative degradation. In a related study, Jiang and co-workers coupled a β-ketoamine COF with graphitic carbon nitride (g-C_3_N_4_) to form a type II heterojunction, establishing a stable channel for electron–hole separation through band alignment.^136^ This composite displayed a band gap of 2.21 eV with an absorption edge extending to 620 nm, enabling 98% RhB removal within 30 min under visible-light irradiation. Mechanistic analysis identified ·O_2_^−^ as the primary ROS, with ·OH contributing synergistically to complete mineralization.

In terms of extending π-conjugation and enhancing charge delocalization, Chen *et al.* developed a COF with extended π-coupling by incorporating large π-conjugated aromatic units into the framework and linking them *via* β-ketoamine bonds to form a fully conjugated skeleton.^137^ This design markedly reduced the exciton binding energy, thereby enhancing electron–hole separation efficiency and facilitating the generation of both ·O_2_^−^ and ·OH under visible-light irradiation. Mechanistic studies revealed that ·O_2_^−^ predominantly initiated chromophore cleavage during the early decolorization stage, whereas ·OH played a crucial role in aromatic ring scission and deep mineralization; the photocatalyst retained its structural integrity and activity over multiple cycles. In another example, Wang *et al.* constructed a three-dimensional cross-linked hetero[6]radial diene COF based on radial D–A units.^138^ The multidirectional electron transport network inherent to this architecture substantially accelerated charge migration and boosted ·O_2_^−^ yields. Under visible-light illumination, the material enabled rapid RhB removal, with a rate constant significantly exceeding that of analogous two-dimensional COFs. Both radical-trapping and ESR measurements confirmed that ·O_2_^−^ was the dominant ROS, with ·OH providing supplementary activity during the mineralization phase. In a related approach, Hou *et al.* enhanced framework polarity, π-conjugation, and structural stability by *in situ* converting reversible imine linkages into rigid thiazole bonds, thereby facilitating O_2_ adsorption–activation and exciton dissociation (Fig. 18).^139^ This thiazole-linked COF achieved 97% RhB removal within 25 min under *λ* > 420 nm irradiation, with ·O_2_^−^ and ^1^O_2_ identified as the dominant ROS. The photocatalyst retained high activity over five consecutive cycles. The catalyst exhibited excellent MO degradation ability under visible light irradiation, almost completely removing 10 mg per L MO within 60 min, and the reaction rate constant was significantly higher than that of traditional imine COFs. Additionally, Zhang *et al.* integrated multiple D–A units into a single COF backbone, optimizing intramolecular charge–transfer pathways and increasing the rate of ·O_2_^−^ generation.^140^ This structural engineering enhanced the RhB degradation rate constant by 1.7-fold compared with a control COF lacking the heterojunction design, with ·O_2_^−^ as the primary species responsible for decolorization and mineralization. The framework retained excellent structural stability throughout repeated use.

**Fig. 17:**
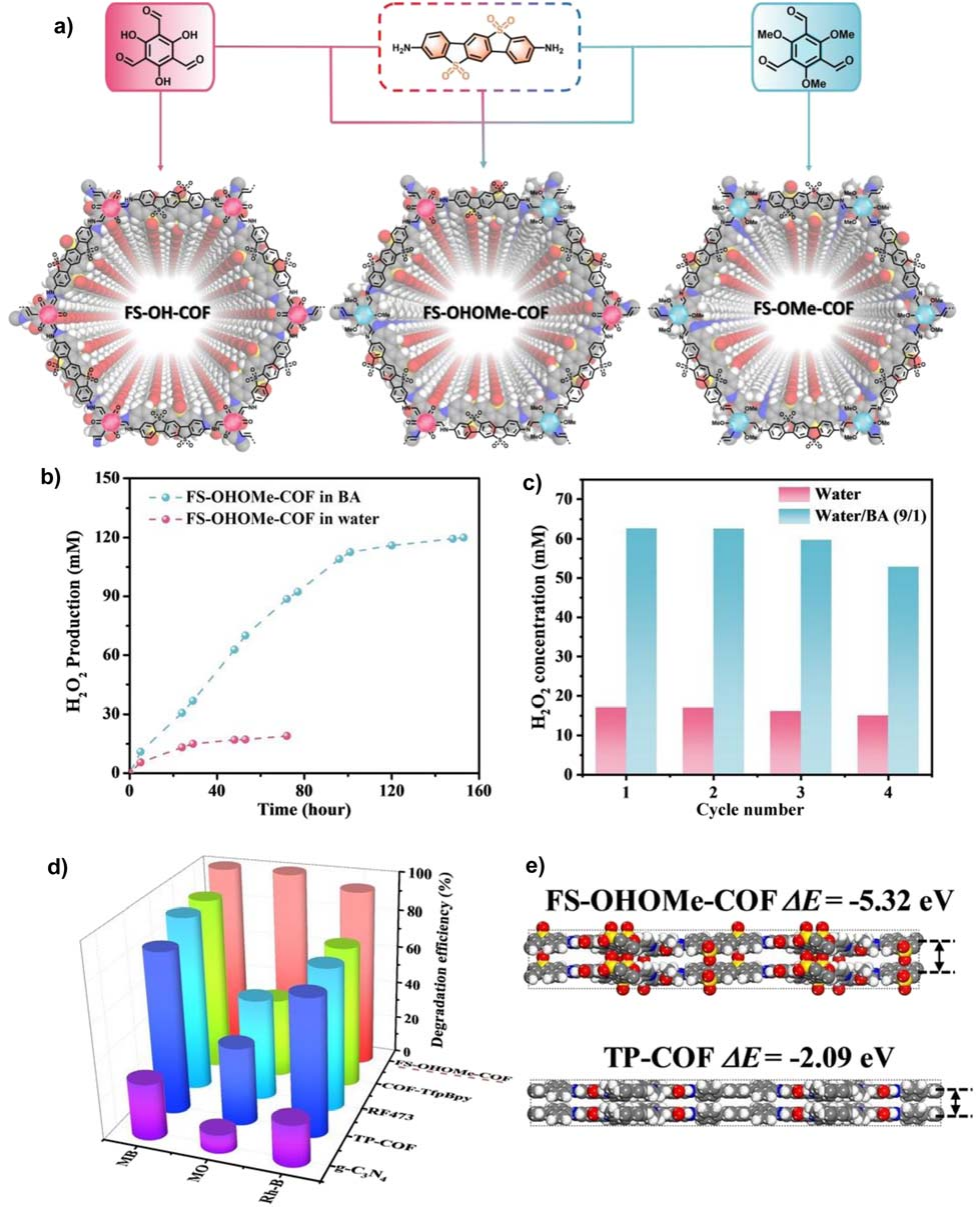
(a) Synthetic routes and chemical structures of sulfone COFs; (b) long-term photocatalytic performance of FS–OHOMe-COF under an O_2_-saturated atmosphere; (c) comparative photocatalytic performance of FS–OHOMe-COF and reference materials in dye degradation; (d) photocatalytic performance of FS–OHOMe-COF over multiple cycles; (e) DFT-calculated interlayer interaction energies of FS–OHOMe-COF and TP-COF. Reproduced with permission from ref. 145, Copyright 2024, from Wiley-VCH.

The incorporation of metal centres and targeted framework functionalization can further enhance the generation and utilization of ROS by modulating local charge-transfer dynamics. For instance, Chen *et al.* employed an ion thermal synthesis to coordinate triazine nitrogen sites with single-atom Co–N_4_ active centres, yielding a COF with a specific surface area of 600 m^2^ g^−1^, an average pore size of 1.5 nm, a band gap of 2.35 eV, and an absorption edge at 530 nm.^141^ Under visible-light irradiation, this catalyst achieved 99% RhB degradation within 40 min, approaching complete mineralization. ESR spectroscopy and radical-quenching experiments revealed that ·O_2_^−^ and ·OH acted synergistically, with ·O_2_^−^ preferentially cleaving the chromophore and ·OH facilitating deep mineralization of the aromatic backbone. In another example, Khojastegi *et al.* synthesized a three-dimensional interlocked metal-COF by *in situ* embedding Cu(i) photosensitizing centres into ordered framework sites, effectively suppressing the flattening and quenching of photosensitizers, extending photocarrier lifetimes, and broadening visible-light absorption.^142^ This material removed >98% RhB within 30 min under *λ* > 420 nm irradiation, retained full activity after five cycles, and operated primarily through ·O_2_^−^ and h^+^, with ·OH playing a minor role. Building on this concept, Xu *et al.* integrated photocatalysis with a Fenton-like process in a copper-porphyrin COF, wherein the Cu centre served both as a photosensitizing site to promote electron–hole pair separation and as an active site for H_2_O_2_ activation to ·OH.^143^ This dual-functionality enabled a synergistic ·O_2_^−^/·OH pathway, achieving >98% RhB removal and 90% TOC reduction within 50 min under visible light with optimal H_2_O_2_ dosage. In addition, the material can efficiently adsorb MB in the dark, and in a photo-Fenton-like system under visible light irradiation and coexistence of H_2_O_2_, the removal rate exceeds 99% within 30 minutes, and the TOC removal rate reaches 85%, showing significant mineralization ability. Mechanistic studies confirmed that ·O_2_^−^ dominated the initial chromophore cleavage, while ·OH and h^+^ facilitated complete aromatic ring mineralization; PXRD and XPS analysis showed no structural degradation over five cycles. In contrast to metal-containing systems, Li *et al.* demonstrated a tunable, metal-free COF functionalized with –OH groups, which enhanced pollutant enrichment *via* electrostatic and hydrogen-bond interactions while promoting electron–hole separation.^144^ This catalyst achieved 94% RhB removal within 60 min under visible light, with ·O_2_^−^ and ^1^O_2_ as the principal ROS, and exhibited excellent structural and catalytic stability across multiple cycles. In a recent study, Shu *et al.* synthesized a highly crystalline β-ketoamine COF with continuous π-conjugated channels by solution-phase polycondensation of D–A monomers followed by thermal treatment (Fig. 17).^145^ The extended conjugation facilitated efficient two-electron reduction of dissolved O_2_ to H_2_O_2_ under visible-light irradiation. Subsequent addition of Fe^2+^ initiated a Fenton-like process, in which Fe^2+^ rapidly reacted with in situ–generated H_2_O_2_ to produce ·OH in solution. This ·OH, together with photocatalytically generated ·O_2_^−^, acted synergistically to accelerate both the decolorization and mineralization of RhB. Notably, this strategy did not require direct photoexcitation at metal centres; instead, it relied on the coupling of metal-free photocatalysis with homogeneous Fenton chemistry. The system exhibited high reaction rates over a broad pH range, underscoring its potential for treating aqueous environments contaminated with diverse organic pollutants.

**Fig. 18:**
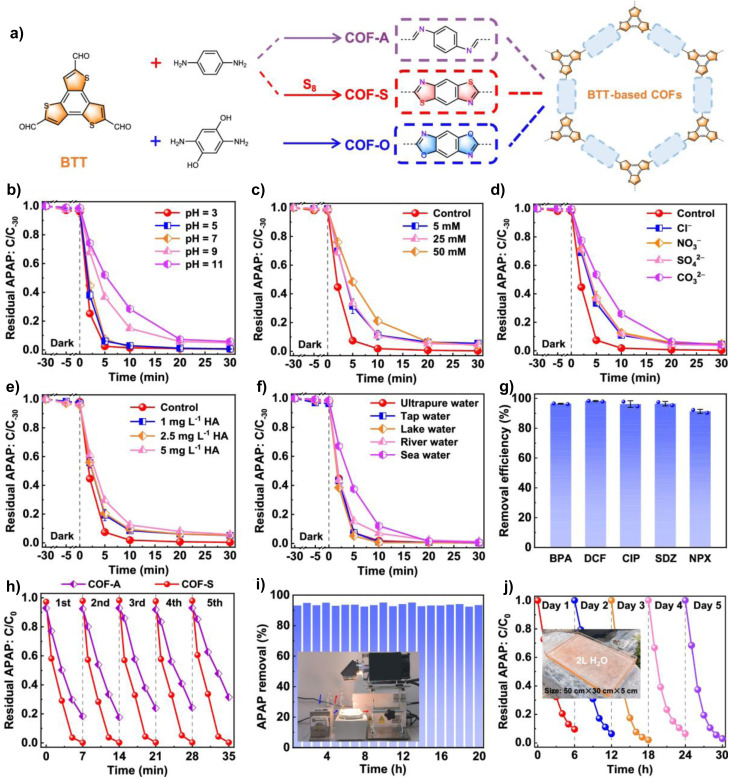
(a) Preparation scheme of COF-A, COF-O, and COF-S; (b) photocatalytic performance of COF-S for paracetamol degradation at different initial solution pH; (c) photocatalytic performance of COF-S under different ionic strengths; (d) photocatalytic performance of COF-S with coexisting anions; (e) photocatalytic performance of COF-S in the presence of humic acid; (f) photocatalytic performance of COF-S in different real water samples; (g) photocatalytic degradation of five additional emerging contaminants by COF-S; (h) reusability of COF-A and COF-S for paracetamol degradation under visible-light irradiation; (i) photocatalytic performance of immobilized COF-S in a continuous-flow reactor under visible-light irradiation for 20 h; (j) photocatalytic performance of COF-S in an enlarged 2 L reactor under natural sunlight irradiation for 5 days (insets: digital images of the degradation experiments in different reactors). Reproduced with permission from ref. 139, Copyright 2024, from Springer.

The optimization of pore architecture and mass-transfer properties is equally critical for enhancing RhB degradation efficiency. Zhang *et al.* fabricated a two-dimensional/two-dimensional BiOBr/TzDa-COF direct Z-scheme heterojunction that maintained high redox potential while markedly improving charge-carrier separation.^146^ This composite degraded 10 mg per L RhB efficiently across a broad pH range, achieving >95% removal and 80% TOC reduction within 60 min, and displayed a pronounced synergistic effect in RhB/Cr(vi) co-contaminant systems. Xu *et al.* reported an ordered meso–microporous single-crystal COF, in which precise band-gap engineering optimized both light-harvesting capability and energy-band alignment, enabling exceptionally high RhB degradation rates.^147^

Under visible-light irradiation, the photocatalyst removed >98% of RhB within 15 min, with an apparent rate constant markedly exceeding that of a non-single-crystalline control. The hierarchical pore network enhanced substrate diffusion and facilitated efficient access to catalytic sites. Reactive species analysis identified ·O_2_^−^ as the dominant ROS, with ·OH and h^+^ synergistically driving deep mineralization. In a related study, Qu *et al.* developed anionic COF aerogels that rapidly concentrated cationic dyes, including RhB, *via* strong electrostatic interactions.^148^ Under visible-light photocatalysis, these aerogels achieved 97% RhB removal within 10 min—significantly faster than their non-aerogel counterparts. Mechanistic investigations attributed this performance to the aerogels' large surface area, interconnected pore channels, and enhanced mass-transfer properties, which together facilitated the adsorption–reaction–product diffusion sequence. The photocatalytic activity was primarily governed by ·O_2_^−^, with minor contributions from ·OH, and both structural integrity and catalytic performance were retained over multiple cycles. In addition to RhB, this catalyst significantly enhances the adsorption and enrichment of MB and the photocatalytic reaction rate. Under conditions with a wavelength of *λ* > 420 nm, the material achieves a MB removal rate of 97% within 15 minutes, with ·O_2_^−^ and h^+^ as the primary active species. The synergistic adsorption-photocatalytic effect is particularly pronounced in the removal of low-concentration pollutants. Furthermore, Xu *et al.* combined a β-ketoamine COF with peroxymonosulfate (PMS) in an oxidant-assisted system, generating ·SO_4_^−^, ·OH, and ·O_2_^−^ simultaneously under visible light.^149^ This system achieved complete RhB degradation within 6 min, representing 12-fold and 8-fold enhancements compared with PMS or the COF alone, respectively. Mechanistically, ·SO_4_^−^ rapidly cleaved the aromatic framework, while ·OH and ·O_2_^−^ drove the deep mineralization of intermediates.

MB is a structurally stable, difficult-to-degrade, and potentially toxic cationic dye that causes light attenuation in aquatic environments, ecological imbalance, and health hazards; conventional remediation methods show limited efficacy. COFs have emerged as promising photocatalysts for achieving complete MB mineralization through tailored structural and electronic designs. Zhao *et al.* constructed a COF featuring an acceptor–donor–acceptor (A–D–A) motif, where a strong electron-acceptor unit was coupled with an electron-rich donor segment to establish a long-range electric dipole within the framework.^150^ This polarity engineering enhanced the built-in electric field, thereby improving charge separation and transport efficiency. Under visible-light irradiation (10 mg per L MB), this COF exhibited markedly higher degradation rate constants and mineralization efficiencies compared to a non-polarity-engineered control. Radical-trapping and ESR analyses identified ·O_2_^−^ and h^+^ as the dominant reactive species, with the dipole field accelerating electron transfer to donor sites for O_2_ reduction, thus facilitating MB structural breakdown and mineralization. Similarly, Sun *et al.* employed a one-step polycondensation of olefin- and imine-containing monomers under mild conditions to form a highly crystalline COF with continuous π-conjugation.^151^ The combined olefin–imine double bonds broadened light absorption and enhanced electron delocalization, reducing electron–hole recombination probability. This photocatalyst achieved >95% MB removal within 30 min under visible light and maintained stable activity over multiple cycles. The photocatalytic degradation of MO by this COF was stable over a wide pH range, with a removal efficiency exceeding 95% at 20 mg per L MO within 50 min and no significant activity decay during five cycles. Mechanistic studies revealed that the conjugated double-bond network enhanced π–π stacking interactions and charge transport, enabling the synergistic generation of ·O_2_^−^ and ·OH, which accelerated oxidative MB decomposition. Zhang *et al.* further advanced MB degradation performance by simultaneously introducing electron-withdrawing and electron-donating groups into a triazine-based COF, thereby forming a multi-donor–acceptor heterojunction with a precisely tuned band structure and charge-transfer dynamics.^140^ The optimized material exhibited high specific surface area, well-ordered mesoporosity, and excellent crystallinity, all of which facilitated active-site exposure and mass transfer. Under visible light, COF-Br@OCH_3_ demonstrated the highest activity, with significantly increased ·O_2_^−^ and ·OH yields while lowering the energy barriers for O_2_ reduction and H_2_O oxidation, resulting in rapid decolorization and deep MB mineralization.

He *et al.* designed a 3D COF incorporating a dual A–D–A building block, in which monomer electronic properties were optimized to enhance visible-light absorption and charge-carrier separation efficiency.^152^ The resulting photocatalyst exhibited excellent redox activity toward multiple organic dyes, including MB and MO. For MO removal, the authors integrated the COF into a photo-Fenton system assisted by H_2_O_2_, further improving oxidative capacity. In particular, a functionalized TAPB–DMTA COF was synthesized in which the pore-size distribution and specific surface area were precisely regulated *via* acetic-acid-assisted synthesis, yielding abundant accessible active sites and facilitating efficient mass transfer. Under visible-light irradiation in the presence of 10 mM H_2_O_2_, 15 mg per L MO was removed by 98% within 30 min, with high activity retained even in complex water matrices containing Cl^−^, SO_4_^2−^, and humic acid. Mechanistic analysis indicated that ·OH and ·O_2_^−^ acted synergistically as the dominant ROS, with the optimized pore structure accelerating pollutant–ROS interactions. Building on this strategy, the same group subsequently prepared a highly crystalline COF containing both olefinic and imine linkages *via* a one-step solvothermal method, thereby achieving simultaneous optimization of π-conjugation and D–A interactions.^153^ This framework exhibited broadened light absorption, enhanced electron–hole separation efficiency, and superior photocatalytic activity toward both MB and MO. Under visible light, the catalyst achieved >95% MB degradation within 30 min and >93% MO removal within 40 min, with negligible activity loss after multiple cycles. Mechanistic studies confirmed that ·O_2_^−^ and ·OH were the principal ROS, while the π-conjugated backbone promoted intermolecular electron delocalization and facilitated ROS generation, accelerating oxidative dye decomposition. Gogoi *et al.* recently developed a metal-free photocatalytic COF *via* a mechanical pulverization strategy, which eliminates the need for high temperatures or organic solvents, rendering the synthesis process green, facile, and scalable.^154^ Under visible-light irradiation, the material exhibited outstanding photocatalytic activity toward both RhB and MB, achieving near-complete RhB removal within a short reaction time and high MB degradation efficiency within similarly brief durations. Active-species quenching experiments combined with EPR analysis confirmed that ·OH and ·O_2_^−^ were the dominant ROS. The synergistic effect of efficient photogenerated charge separation and accelerated mass-transfer kinetics facilitated abundant ROS production, thereby enabling the rapid oxidative decomposition of dye molecules. In a related study, Yang's group reported a functionalized TAPB–DMTA COF synthesized with acetic-acid-mediated pore-size control, yielding higher specific surface area, more optimal pore geometry, and greater active-site accessibility.^155^ Coupled with a photo-Fenton process, the COF delivered exceptional MO degradation efficiency—98% removal of 15 mg L^−1^ within 30 min—while retaining performance in the presence of common ionic and organic interferents. The cooperative action of ·OH and ·O_2_^−^ was again identified as the primary degradation pathway, highlighting the pivotal role of pore engineering in enhancing ROS–pollutant contact kinetics and ensuring high environmental adaptability.

Peng's research group enhanced the mass-transfer efficiency and active-site accessibility of COFs for Fenton-like reactions by tailoring their porosity and specific surface area.^156^ The resulting high-porosity COFs exhibited markedly accelerated degradation kinetics and superior mineralization efficiency toward malachite green (MG) under photo-Fenton conditions. Mechanistic investigations revealed that the optimized pore architecture not only facilitated the rapid diffusion of pollutant molecules to the catalytic centres, but also promoted the activation of H_2_O_2_ to generate ·OH, thereby boosting both reaction rates and resistance to common water-matrix interferences. This work provides a viable structural-engineering strategy for constructing high-performance photocatalytic–Fenton synergistic systems.

#### 4.3.2 Degradation of antibiotics and drugs

The widespread use of antibiotics and pharmaceuticals across critical sectors—particularly in medicine and animal husbandry—has led to their persistent release and accumulation in aquatic environments, constituting a significant global water pollution challenge.^157^ These contaminants are characterized by high biological activity and environmental persistence, and even trace concentrations can promote the emergence of drug-resistant strains, disrupt ecological balance, and pose serious threats to human health.^158^ Conventional water treatment methods, including coagulation–sedimentation, adsorption, and biodegradation, often suffer from low removal efficiencies, poor selectivity, and the risk of secondary pollution. In contrast, photocatalytic oxidation represents a green and efficient advanced oxidation technology capable of completely mineralizing antibiotics and pharmaceuticals under mild conditions by harnessing visible light to generate a spectrum of highly ROS. COFs, with their long-range ordered architectures, tunable functional groups, and outstanding photoelectric properties, have emerged as ideal platforms for developing high-performance photocatalytic systems.^159^ By precisely engineering their electronic structures, pore architectures, and photosensitizing centres, COFs can deliver efficient, stable, and broad-spectrum removal of antibiotics and pharmaceuticals under visible-light irradiation, providing a promising pathway toward sustainable and environmentally benign water treatment technologies.

Zhao *et al.* employed band and energy level engineering to tailor an A–D–A motif COF incorporating quinone and triazine dual acceptor units, thereby strengthening the built-in electric field and enhancing charge carrier separation.^150^ Under visible light irradiation, this material achieved 91% degradation of ciprofloxacin (CIP) within 50 minutes and over 92% removal of erythromycin (ERY), with h^+^ and ·O_2_^−^ identified as the dominant reactive species and ^1^O_2_ also contributing to the process. Sun *et al.* designed a highly crystalline COF featuring both ethylene and imine double bonds, where the extended π-conjugation and enhanced framework polarity facilitated >95% removal of amoxicillin (AMX) within 60 minutes. Reactive species analysis confirmed the synergistic roles of ·O_2_^−^ and h^+^ in the degradation process.^151^ A similar band gap tuning approach was reported by Chen *et al.*, who constructed fully π-conjugated vinylene-linked COFs from diacetylene and triazine building blocks.^160^ The incorporation of diacetylene units markedly extended π-electron delocalization and fine-tuned the band structure, yielding exceptionally high photocatalytic activity and structural stability. This material demonstrated outstanding performance in degrading representative organic pollutants, including phenols and norfloxacin (NOR), achieving >96% NOR removal within 15 minutes, indicative of ultrafast reaction kinetics and broad-spectrum applicability. Shi *et al.* further developed a COF-TzDa/Ag/AgBr heterojunction, where the surface plasmon resonance effect of photogenerated Ag^0^ facilitated efficient Z-scheme charge transfer.^161^ This system removed 97% of tetracycline (TC) within 60 minutes and retained high activity in a TC/Cr(vi) mixed system, with ·O_2_^−^ and h^+^ identified as the primary reactive species.

Zhang *et al.* synthesized a multi-donor–acceptor heterojunction COF that enhanced ·O_2_^−^ generation *via* a spatially zoned ICT pathway, leading to a 1.5–2-fold increase in the degradation rate constants of sulfamethoxazole (SMX), ciprofloxacin (CIP), and amoxicillin (AMX) compared with the unmodified framework.^140^ Tong's group introduced thiazole linkages to increase framework polarity while preserving efficient π–π stacking, thereby facilitating O_2_ adsorption and activation.^139^ This COF achieved >88% removal of ofloxacin (OFX) within 40 minutes and nearly 90% removal of chloramphenicol (CAP) within 50 minutes, with ·O_2_^−^ and ^1^O_2_ identified as the primary reactive species. A metal-free tunable COF further improved the degradation efficiency of tetracycline (TC) and SMX through the incorporation of –OH groups, with ·O_2_^−^ and ^1^O_2_ acting as the dominant ROS. Li *et al.* developed a COF functionalized with carboxyl groups, enabling simultaneous chelation of divalent metal ions and degradation of antibiotics in livestock and poultry wastewater.^144^ Khojastegi *et al.* reported a three-dimensional interlocked metal-COF in which Cu(i) photosensitizing centres were anchored at ordered sites within the framework, significantly prolonging the lifetime of photogenerated charge carriers.^142^ Under visible light, removal efficiencies for TC, oxytetracycline (OTC), norfloxacin (NOR), and SMX all exceeded 90%, with ·O_2_^−^ and h^+^ as the dominant active species. To address pharmaceutical contaminants, a β-ketoamine COF with tunable nitrogen positions was designed to modulate the band structure and dipole moment by varying the number and position of heterocyclic nitrogen atoms.^162^ The *ortho*-nitrogen variant achieved nearly complete degradation of 10 mg per L acetaminophen (APAP) within 60 minutes under visible light (*λ* > 420 nm), with a TOC removal rate exceeding 80%. ROS analysis confirmed a synergistic contribution from ·O_2_^−^ and ^1^O_2_.

#### 4.3.3 Degradation of phenolic pollutants

Phenolic compounds, ubiquitous in industrial effluents, present a distinct challenge due to their high toxicity and stability. The designable active sites and tunable redox properties of COFs are specifically leveraged to generate potent ROS for the efficient breakdown and mineralization of these persistent pollutants. Phenols and their derivatives are widely used in industrial processes such as plastics, resins, dyes, and pesticides. Due to their high toxicity, persistence, and endocrine-disrupting effects, they are considered typical environmental persistent organic pollutants.^163^ Phenol can disrupt cell membrane integrity and inhibit the activity of various enzymes. Bisphenol A (BPA) has been shown to have significant reproductive toxicity and potential carcinogenicity, posing a serious threat to both ecosystems and human health. Traditional adsorption, oxidation, and membrane separation methods often suffer from high energy consumption, poor selectivity, and secondary pollution when treating these pollutants.^164^ Therefore, developing photocatalytic degradation strategies centred around COFs, leveraging their designable band structures, abundant active sites, and excellent chemical stability to efficiently generate active species under visible light irradiation and achieve complete mineralization of phenols and their derivatives, has become a key focus of current environmental remediation research.

**Fig. 19:**
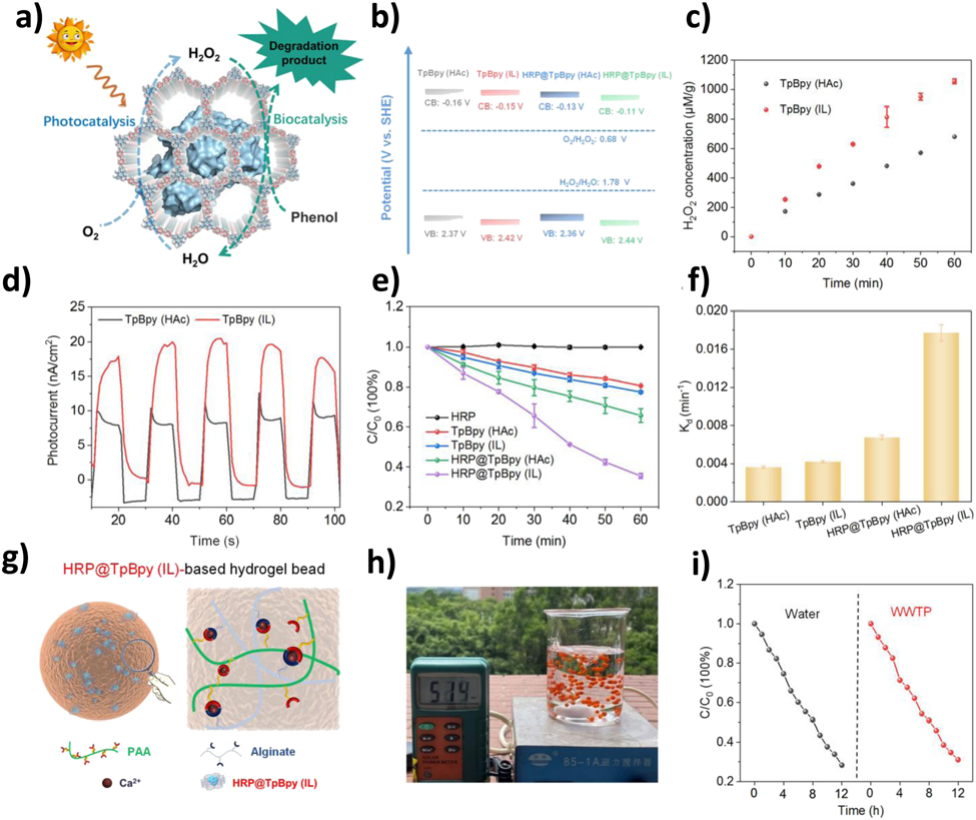
(a) Illustration of phenol degradation by photo-enzyme coupled catalysis using the HRP@TpBpy ensemble; (b) electronic band structures of TpBpy (HAc), TpBpy (IL), HRP@TpBpy (HAc), and HRP@TpBpy (IL); (c) photocatalytic performance of TpBpy (IL) and TpBpy (HAc) for *in situ* H_2_O_2_ production in pure water (*λ* > 400 nm, 298 K, xenon lamp, light intensity at 400–700 nm: 550 W m^−2^; 10 mL water, 1 mg catalyst); (d) photocurrent spectra of TpBpy (HAc) and TpBpy (IL); (e) dynamic curves of phenol degradation with different catalysts; (f) apparent degradation rate constants (*k*_d_) of phenol over different catalysts; (g) structure of HRP@TpBpy (IL)-based hydrogel beads prepared *via* double-crosslinked alginate gelatinization; (h) digital image of phenol degradation in real water using HRP@TpBpy (IL)-based hydrogel beads under sunlight; (i) degradation dynamics of phenol over HRP@TpBpy (IL)-based hydrogel beads under sunlight in pure water and WWTP wastewater. Reproduced with permission from ref. 166, Copyright 2024, from Wiley-VCH.

In the degradation of phenolic pollutants, diverse structural engineering and functionalization strategies have been developed. You *et al.* constructed a D–A heterojunction within the COF skeleton, which effectively promoted photogenerated charge separation and migration.^165^ As a result, rapid phenol removal was achieved within 90 minutes under visible light, while simultaneously driving the hydrogen evolution reaction, highlighting the coupling potential of pollutant control with energy conversion. Gao *et al.* reported an enzyme@COF light-enzyme cascade system prepared by an ionic liquid-mediated dynamic polymerization method (Fig. 19).^166^ This hybrid platform could simultaneously generate H_2_O_2_ and drive enzymatic oxidation under mild aqueous conditions, with a phenol degradation rate 2.63 times higher than that of low-crystalline analogues, underscoring the advantages of light-enzyme synergy in enhancing both degradation efficiency and selectivity. Zadehnazari *et al.* designed a tetranitrogen-linked COF capable of efficiently producing ·O_2_^−^ and ·OH under light irradiation,^88,167^ showing excellent performance in the degradation of phenol and other pollutants and demonstrating the potential of dynamic-bond COFs in complex wastewater treatment. Furthermore, Wang *et al.* introduced Fe species into a COF skeleton to construct Fe@COF composites.^168^ These materials could efficiently activate H_2_O_2_ under weakly acidic to neutral conditions, markedly improving ·OH generation and exhibiting outstanding degradation activity toward phenol and its derivatives, thus overcoming the conventional Fenton reaction's limitation of requiring strongly acidic media.

Progress has also been made in the removal of BPA. Sun *et al. in situ* constructed CdS/COF heterojunctions, which effectively suppressed CdS photocorrosion and enhanced interfacial charge separation.^169^ Under neutral conditions, the composite achieved 85.68% BPA removal within 3 h, with h^+^ and ·O_2_^−^ identified as the dominant reactive species. This strategy ensured both high photostability and degradation efficiency, while avoiding the deactivation issues common to traditional semiconductors, offering important guidance for COF–semiconductor hybrid design. Yang *et al.* further advanced this concept by covalently integrating the MOF@COF into a Z-scheme heterojunction, combining rapid adsorption with efficient photocatalysis.^170^ In this system, MOFs provided highly dispersed adsorption sites, whereas the π-conjugated COFpromoted effective electron–hole separation. The composite completely removed 100 ppm of BPA within 10 minutes and exhibited broad-spectrum activity against pollutants such as phenol, tetracycline, and rhodamine B. These findings demonstrate that structural integration to achieve dual functional synergy is a promising route for addressing the challenge of multi-pollutant wastewater. In terms of mechanistic innovation, Deng *et al.* designed D–A sp^2^-conjugated COFs with finely tuned band structures, achieving high visible-light activity and continuous *in situ* H_2_O_2_ generation at rates up to 1952 µmol g^−1^ h^−1^ without sacrificial agents.^171^ This cascade pathway of “H_2_O_2_ self-generation + ROS-driven degradation” enabled 96% BPA removal within 60 minutes, highlighting a sustainable approach that avoids additional chemical inputs. Building on this principle, Zhou *et al.* constructed a bipyridine-based polyimide COF platform capable of simultaneously producing H_2_O_2_ and driving BPA degradation under visible light.^172^ The material exhibited outstanding stability and recyclability, retaining high efficiency without structural or functional decay over multiple cycles. This work not only illustrates a green strategy for environmental remediation but also exemplifies the dual role of COFs in clean energy conversion and pollutant degradation driven by renewable light energy.

Although numerous studies have demonstrated the excellent photocatalytic degradation performance of COFs against target pollutants under ideal laboratory conditions,^129,140^ their performance in complex, multi-phase real environmental systems are the key to assessing their practical application potential. The ubiquitous presence of natural organic matter (NOM), coexisting ions, suspended particles, and pollutant mixtures in real environments primarily affects COF performance through the following mechanisms: (i) pore blockage and active site masking: the physical adsorption of macromolecular NOM or colloidal particles can block micropore channels, hindering mass transfer. (ii) Competitive adsorption and reaction quenching: background species compete with target pollutants for adsorption sites while also acting as scavengers for ROS.^149^ (iii) Optical shielding effect: the color or turbidity of water attenuates incident light intensity, reducing photon utilization efficiency. (iv) Long-term chemical/structural stability: fluctuating pH, high salinity, or microorganisms in real water bodies may erode the COF, leading to structural collapse and activity loss. Although reports specifically investigating the behaviour of COFs in complex matrices remain relatively limited, preliminary work has begun to focus on this direction, for instance, exploring the performance decay of COFs in degrading antibiotics in simulated seawater or solutions with high background ion concentrations,^161^ or studying the influence of humic acid on their photogenerated charge behaviour.^162^ These studies highlight the urgency of evaluating COF performance under near-realistic conditions. Looking forward, advancing the practical environmental application of COFs requires efforts in the following aspects: first, developing COFs with hierarchical pore structures^65^ or anti-fouling surfaces to alleviate clogging and enhance selective mass transfer. Second, strengthening the specific recognition and adsorption of target pollutants through precise molecular engineering. Third, developing frameworks based on high-stability linkage chemistries to resist environmental erosion. Finally, there is an urgent need to establish standardized performance evaluation protocols that systematically test the long-term activity and stability of COFs in real water bodies containing representative background components or complex simulated matrices, providing a reliable basis for engineering applications.

### 4.4 Biomedical applications

In recent years, the utilization of light energy for efficient and controllable disease treatment and infection control has emerged as a critical focus in biomedical materials research. Conventional PDT, which relies on photosensitizers to generate ROS, is hindered by several intrinsic limitations, including aggregation-induced quenching of photosensitizer molecules, poor photostability, and low ROS yields in the hypoxic or complex microenvironments of tumors and infections.^173^ Similarly, photothermal therapy (PTT), which destroys lesions *via* localized hyperthermia, often suffers from limited selectivity and inconsistent efficacy when used as a monomodal strategy.^174^

COFs provide an elegant solution to these challenges. Their crystalline porous structures offer a stable platform to immobilize and organize photoactive components, while their tunability allows optimization towards specific biological applications. COFs as a new class of crystalline porous materials with ordered π-conjugated skeletons and tunable pore microenvironments, provide a promising platform to overcome these bottlenecks.^175^ The rigid backbone of COFs can immobilize photosensitizer units, thereby suppressing aggregation-induced quenching. Meanwhile, their extended π-conjugated networks enhance light harvesting and ISC, markedly increasing the yield of ^1^O_2_ and other ROS.^176^ Beyond these inherent photophysical advantages, the chemical versatility of COFs allows for the incorporation of oxygen-storage moieties, the construction of parallel type I/II energy-level architectures, and even the integration of heterojunction or composite systems to couple PDT with PTT for synergistic therapeutic effects.

In the context of PTT, COFs not only act as efficient photothermal conversion scaffolds but also frequently serve as dual-function agents in combined PDT–PTT modalities.^177^ The synergy between localized hyperthermia and ROS-mediated oxidative damage can promote apoptosis and necrosis, while also stimulating immune responses, thereby enhancing tumor suppression. In antibacterial applications, COF-based photocatalysts show unique advantages under visible or near-infrared irradiation.^178^ Photosensitizing motifs such as porphyrins or BODIPY embedded in the framework can rapidly produce ROS, which disrupt bacterial membranes and metabolic pathways. When further coupled with photothermal heating or the release of gaseous therapeutic factors, these systems exhibit potent bactericidal activity, even against drug-resistant strains and biofilms. Importantly, this antimicrobial phototherapy is non-invasive, associated with a low risk of resistance development, and benefits from the inherent biocompatibility and biodegradability of COFs, underscoring its translational potential.^179^ The following subsections detail how COFs are engineered for photodynamic/photothermal therapy and antimicrobial applications, highlighting the translation of photocatalytic ROS generation from chemical concepts to biological efficacy.

#### 4.4.1 Photothermal therapy of COFs based on photocatalytic oxidation

COFs are redefining the material paradigm for PDT and PTT by exploiting their extended π-conjugation pathways, programmable backbone chemistry, and tunable pore microenvironments. A pioneering study by Zhang *et al.* demonstrated that directly embedding photosensitive moieties such as porphyrins into ordered COF skeletons can enhance charge delocalization and energy transfer while suppressing aggregation-caused quenching (ACQ), thereby boosting ^1^O_2_ yields and providing a stable, post-functionalizable photosensitizing platform.^180^ Building on this concept, Luan *et al.* employed extended π-conjugation and “bond engineering” strategies to improve photogeneration efficiency.^181^ Using the Debus–Radziszewski multicomponent condensation method, they developed an imidazole-linked photosensitive COF that exhibited both high structural stability and efficient ^1^O_2_ release, while maintaining robust photosensitivity under cellular and physiological conditions. Chen *et al.* further advanced the nanoscale formulation of COFs by synthesizing carbon dot-based nCOFs *via* Schiff-base condensation of aldehyde functionalized carbon dots with diamines/BODIPY, followed by PEGylation to enhance water dispersibility and cellular uptake.^182^ These nCOFs demonstrated significant tumor suppression and cell proliferation inhibition in both *in vitro* and *in vivo* models, accompanied by stable ^1^O_2_ generation and excellent biocompatibility. In the direction of “theranostic integration,” Gao *et al.* reported COF-survivin, a porphyrin-based COF coupled with antisense oligonucleotides (ASOs) to achieve FRET-mediated imaging, PDT activity, and prognosis monitoring in a single platform. Both live/dead staining and immunoblotting confirmed pronounced apoptosis phenotypes, while H&E histology revealed substantial tumor necrosis and prolonged survival *in vivo*.^183^ Collectively, these studies highlight that skeleton-level photosensitizer integration and bond-type optimization, combined with nanoscale formulation strategies, are critical for enhancing both the therapeutic efficacy and clinical usability of COF-based PDT/PTT systems.

Two effective strategies have recently been proposed to overcome the bottleneck posed by the hypoxic tumor microenvironment in solid cancers: (i) oxygen storage/release and “afterglow” PDT and (ii) type I/II parallel reaction pathways with reduced oxygen dependence. Wang *et al.* post-modified TpDa-COF with a pyridone (Py) side chain to reversibly capture photogenerated ^1^O_2_ as an addict, which could then be released on demand under physiological temperature or 808 nm thermal stimulation.^184^ This design enabled “afterglow” PDT with extended ROS lifetime, achieving an IC_50_ of 0.54 in HeLa cells and continuous ROS release that led to pronounced tumor regression in mouse models, thereby verifying the advantages of segmented generation and prolonged activity of ^1^O_2_. Similarly, Dutta *et al.* introduced anthracene moieties into COFs as endoperoxide (EPO) oxygen-storage units, combining them with a photothermal component to enable heat-triggered ^1^O_2_ release.^185^ This strategy maintained strong ROS levels under hypoxic conditions and demonstrated clear *in vivo* tumor inhibition and histological evidence of therapeutic efficacy, highlighting a materials science route toward “oxygen-independent PDT”.

**Fig. 20:**
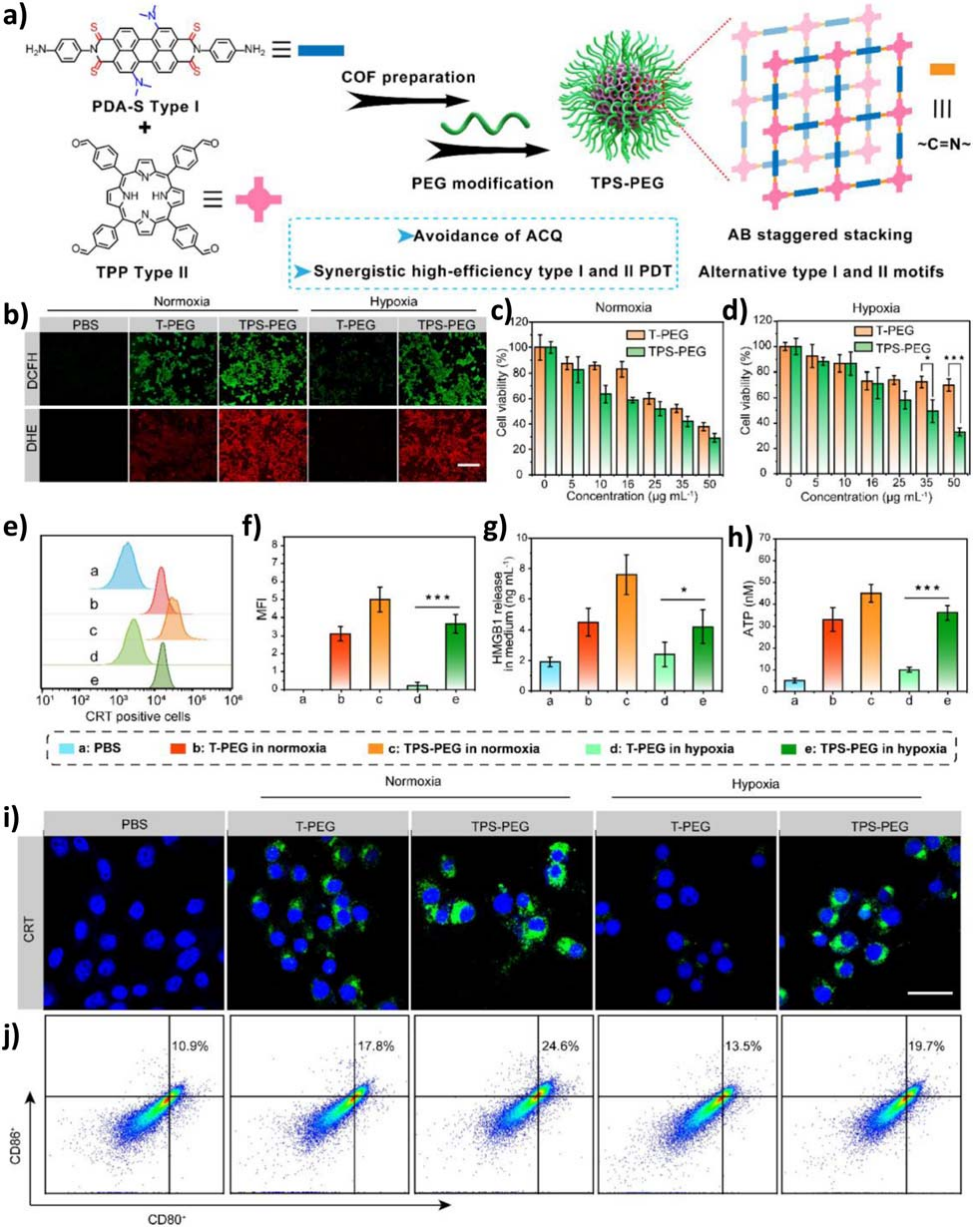
(a) Schematic illustration of the preparation of COF nanophotosensitizers with staggered type I and II photosensitizer motifs; (b) fluorescence microscopy images of 4T1 cells under normoxia or hypoxia after treatment with different groups in the presence of DCFH or DHE upon light irradiation; (c and d) cell viability of 4T1 cells after treatment with TPS-PEG or T-PEG under normoxia and hypoxia at different concentrations upon light irradiation; (e) flow cytometric analysis of CRT expression on the surface of 4T1 tumor cells after treatment with different groups under normoxia or hypoxia; (f) CLSM images and (g) quantitative analysis of CRT expression on 4T1 tumor cells under normoxia or hypoxia; (h) quantitative analysis of HMGB1 release from 4T1 tumor cells using an HMGB1 ELISA kit; (i) quantitative analysis of ATP secretion from 4T1 cells after various treatments using an ATP ELISA kit; (j) quantitative analysis of matured BMDCs after treatment with different groups. Reproduced with permission from ref. 186, Copyright 2024, from American Chemical Society.

In parallel, type I/II hybrid approaches have also emerged. Zhou *et al.* designed a PEGylated COF photosensitizer by alternating type I and type II photosensitive units within the framework layers and stacking them interlayer (Fig. 20).^186^ This architecture enabled the simultaneous generation of ^1^O_2_ and O_2_˙^−^ under both normoxic and hypoxic conditions, effectively inducing immunogenic cell death (ICD). When combined with immune checkpoint blockade (ICB), this system reversed the immunosuppressive tumor microenvironment, ablated large hypoxic *in situ* tumors, and suppressed distant metastases. Likewise, Wei *et al.* synthesized Por-DETH-COF, which features dual type I/II channels under 660 nm red light.^187^ This system not only selectively induced apoptosis in glioma cells but also directionally oxidized thiol groups into disulfides, reflecting the multifunctional and hypoxia-adaptive potential of a single COF platform within complex biological and chemical environments.

Relying on the unique pore environment and designable skeleton of the COF, researchers have been continuously promoting its application in photodynamic and photothermal synergistic therapy in recent years. In terms of coupling monomeric photosensitive units, Zhao *et al.* covalently integrated porphyrin with BODIPY fragments to construct Tph–BDP-COF.^188^ By reducing the excited state energy level difference Δ*E*(S_1_–T_1_), ISC was promoted, while non-radiative attenuation was enhanced, so that the material has both strong ROS production capacity and 29.9% photothermal conversion efficiency. This COF achieved 97% killing of *Escherichia coli* under the synergy of PDT/PTT, which is far better than the 54% achieved by traditional COF-366. EPR and transient absorption experiments further revealed the parallel generation of ^1^O_2_ and O_2_˙^−^ and the improvement of charge separation. In response to this, Sun *et al.* introduced the NO donor BNN6 into TP-Por covalent organic nanosheets to construct a degradable heterojunction that integrates PDT, PTT and gas therapy. Under 635 nm light irradiation, the system can heat the solution to 55 °C (PTCE ≈ 18.4%) within 6 minutes and exhibit significant antibacterial activity and wound healing ability, while alsoshowing good biodegradability and compatibility.^189^

Further multimodal expansion is reflected in the combination with other therapeutic methods. Tai *et al.* constructed Zr-MOF@thin COF shell nanocapsules, which not only enhanced ROS generation through the COF shell, but also induced immunogenic cell death, thereby enhancing radiotherapy sensitivity.^190^ In a soft tissue sarcoma model, the material significantly delayed tumor progression and reduced the risk of recurrence, demonstrating the high complementarity of phototherapy and radiotherapy. Liang *et al.* embedded the pillar aromatic unit into the COF skeleton and loaded the thioacetal prodrug to construct a self-amplified antibacterial PDT platform.^191^ After 20 minutes of illumination, the signal decayed by about 90%, and the *in situ* release of cinnamaldehyde brought about concentration-dependent bacterial inhibition. Simultaneous imaging and micro-CT confirmed its therapeutic benefit *in vivo*. In the heterogeneous composite design, Pang 's group used the solvothermal method to synthesize COF-LZU-1 and *in situ* grew a CuSe shell on its surface to construct a stable core–shell structure, combining the stable ^1^O_2_ production capacity of the COF with the strong photothermal performance of CuSe.^192^ In a mouse model, the material achieved significant tumor inhibition with low toxicity and side effects, showing excellent biosafety. In addition, He *et al.* reported that AQ4N@THPP/TK-PEG uses a ^1^O_2_ cleavable disulfide bond to bind the hypoxia-activated prodrug banoxantrone (AQ4N), designing a cascade pathway from PDT to chemotherapy.^193^ The drug release rate jumped from 8.9% to 78.8% (4 h) under light irradiation and demonstrated significant tumor inhibition and life extension effects in animal experiments, providing a paradigm for precision treatment in hypoxic environments. The work of Tang *et al.* further expanded the therapeutic model of the COF. They constructed an iron porphyrin-based COF (FeTPD) through Schiff base condensation and loaded glucose oxidase (GOx) in the pores to form an FeTPD@GOx composite system.^194^ This design uses the regular pores of the COF to achieve substrate enrichment and interfacial catalysis. GOx can convert glucose in tumors into H_2_O_2_, and the Fe^3+^/Fe^2+^ cycle in the skeleton further converts H_2_O_2_ into O_2_ and ·OH through a Fenton-like reaction; under ultrasonic excitation, the porphyrin unit can efficiently produce active ^1^O_2_. As a result, FeTPD@GOx simultaneously triggers the synergistic effect of SDT (^1^O_2_) + CDT (·OH), showing a stronger ROS amplification effect than a single sonosensitizer. In a 4T1 bilateral tumour model, FeTPD@GOx combined with ultrasound achieved proximal tumour clearance within 6 days, inhibited distal tumour growth within 4 days, and reduced tumour weight to only 5–8% of the control group after 15 days, with no toxicity observed in major organs. This result demonstrates that COF pores not only serve as a structurally stable Sono sensing platform but also amplify ROS production through sequential catalysis, opening up new avenues for the application of COF-based materials in SDT/CDT synergistic tumor therapy.

#### 4.4.2 Antibacterial activity and disinfection

Parallel to their use in cancer therapy, the potent ROS-generating capability of COFs under light irradiation is being effectively harnessed to combat bacterial infections, offering a promising alternative to conventional antibiotics in the face of rising antimicrobial resistance. With the increasing prevalence of antibiotic resistance in recent years, the development of efficient, safe, and sustainable antimicrobial strategies has become a major challenge in the environmental and biomedical fields. COFs with their highly ordered backbone structure, designable energy band modulation capabilities, and excellent chemical stability, provide a unique platform for photocatalytic antimicrobial activities.^195^ By rationally manipulating molecular energy levels, constructing heterojunctions and active sites, and integrating COFs into multifunctional systems, researchers have achieved precise control over the migration of photogenerated e^−^ and h^+^ and the generation of ROS, significantly improving the efficiency of photocatalytic antimicrobial activities.

In the design of molecular energy bands, researchers have enhanced the separation of photogenerated carriers and increased ROS production through intermolecular interactions, D–A architectures, and conjugation extension strategies. He *et al.* constructed a localized electron-regulated micro-environment by introducing C–F⋯C=O intermolecular interactions into the COF skeleton.^196^ These non-covalent weak interactions not only modulated the electron cloud distribution within the framework but also promoted charge enrichment at C=O sites, effectively suppressing electron–hole recombination. As a result, the COF generated H_2_O_2_ through a dual pathway under visible light, significantly increasing ROS levels. In antibacterial tests, the material almost completely inactivated *E. coli* within 30 minutes and maintained continuous disinfection activity, highlighting molecular interaction engineering as an effective strategy to enhance photocatalytic antibacterial performance. Building on this principle, Zhu *et al.* designed a D–A type π-conjugated COF by incorporating benzotrithiophene (BTT) into the framework.^197^ This modification broadened the light absorption range, improving photon utilization under visible light. More importantly, BTT, as a strong electron donor, enhanced electron–hole separation, thereby boosting ROS production. The material not only decomposed the mustard gas simulant CEES within minutes but also achieved >99% inhibition against both Gram-positive *S. aureus* and Gram-negative *E. coli*, demonstrating the dual functionality of detoxification and antibacterial applications. Natural molecule incorporation has also proven effective. Wang *et al.* employed a quercetin-sensitization strategy, coupling natural polyphenol molecules with the COF skeleton to form a stable D–π–A structure.^198^ Benefiting from quercetin's strong light-harvesting and energy-level regulation ability, the system exhibited longer excited-state lifetimes and faster electron transfer than the control group. Under illumination, the material rapidly generated H_2_O_2_ and effectively inhibited the growth of *E. coli* and *S. aureus*, showcasing the unique value of natural molecules in COF photocatalytic antibacterial design.

**Fig. 21:**
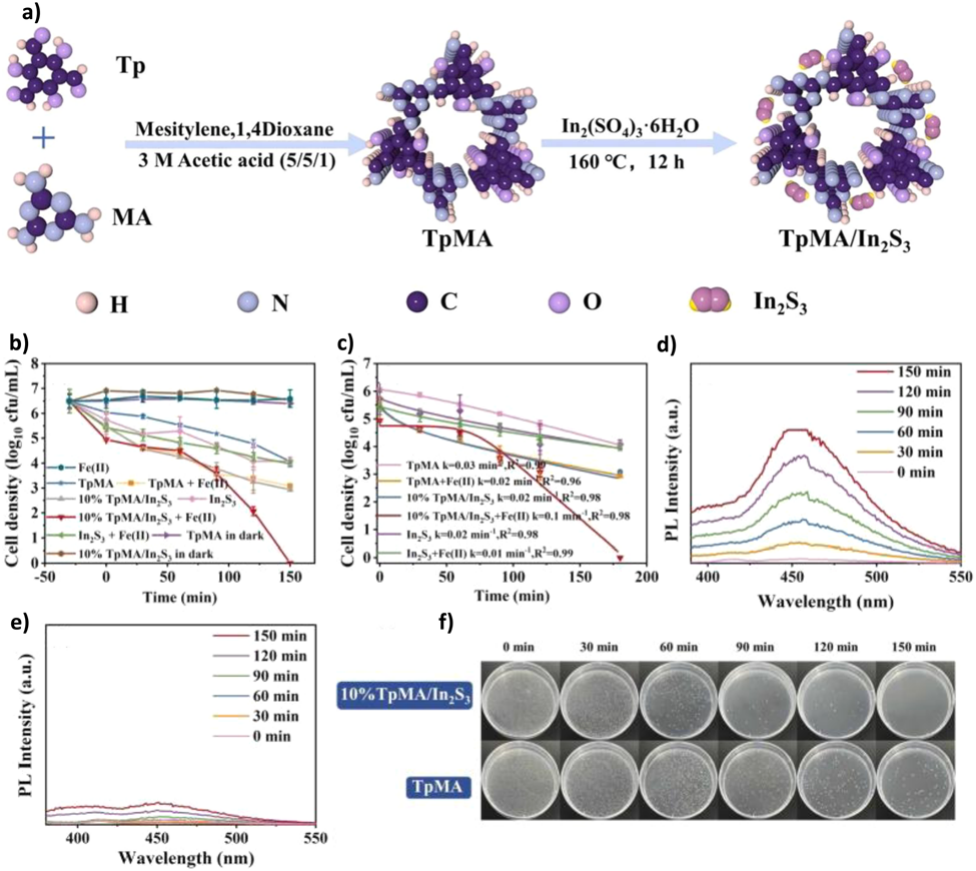
(a) Schematic illustration of the synthesis of TpMA/In_2_S_3_; (b) photocatalytic bacterial inactivation efficiency with and without Fe^2+^ addition; (c) photocatalytic inactivation kinetics of *E. coli* in the presence of 10% TpMA/In_2_S_3_ and Fe^2+^ under xenon lamp irradiation; (d and e) fluorescence spectra of coumarin solution with (d) and without (e) Fe^2+^ in 10% TpMA/In_2_S_3_ under visible light irradiation; (f) photographic images of coated plates with bacterial solutions sampled at different time intervals. Reproduced with permission from ref. 202, Copyright 2024, from Elsevier.

Ding *et al.* advanced this concept with multi-component electronic configuration reprogramming, introducing multiple electron-acceptor units into the COF backbone to spatially control charge distribution.^199^ This design minimized local electron–hole recombination, markedly improving H_2_O_2_ photosynthesis. Antibacterial experiments confirmed rapid inactivation of multiple bacterial strains, demonstrating excellent bactericidal efficiency. Further extending absorption capability, Zhang *et al.* developed an acridine-based COF photosensitizer, leveraging acridine's planar rigidity and strong electron affinity to expand light absorption into the visible and near-infrared regions.^200^ This design significantly improved ROS generation and enabled the COF to efficiently eliminate drug-resistant bacteria such as MRSA, underscoring its potential for clinical translation. A related approach was reported by Wang *et al.* who introduced polyfluorinated groups into the COF skeleton to act as electron storage units.^201^ This design allowed temporary electron storage and controlled release, effectively prolonging the carrier lifetime and further enhancing antibacterial performance. In addition to molecular and band-level optimization, amplifying charge utilization efficiency through interfaces and active sites represents another critical design strategy. A representative example is the construction of an S-scheme heterojunction (Fig. 21).^202^ By coupling COFs with In_2_S_3_, a built-in electric field is formed at the interface, which not only preserves strong redox potentials at both ends but also directionally separates e^−^ and h^+^. This enables a two-step reduction of O_2_ into H_2_O_2_ by photogenerated e^−^, while h^+^ oxidize H_2_O or intermediates, thereby significantly enhancing H_2_O_2_ production and achieving rapid sterilization. This highlights the direct value of “interface-driven” photocatalytic antibacterial activity.^203^ In parallel, ionic COFs achieve a similar acceleration of charge separation without the need for external semiconductors. Symmetrical polarization units within the framework establish a localized built-in electric field, while the positively charged skeleton segments selectively adsorb Gram-positive bacteria. This dual function allows simultaneous imaging and sterilization, achieving >99% clearance efficiency under low-dose, short-term irradiation, demonstrating the potential for precision antimicrobial interventions in complex microbial communities.

If heterojunctions and built-in electric fields address the challenge of “separating e^−^ and h^+^,” then metal centres—particularly single-atom sites—focus on utilizing the separated charges. In a copper–porphyrin-doped polyphenolic aldehyde COF, the porphyrin moiety acts as an efficient photosensitizer, while Cu centres provide quasi-enzyme-like catalytic sites.^204^ Together, they synergistically amplify the generation of ·O_2_^−^, ^1^O_2_, and ·OH. Under visible light, this material suppressed the survival of *S. aureus* and *E. coli* to extremely low levels and remained effective against drug-resistant strains, underscoring the effectiveness of the dual-channel amplification of photosensitization and metal-site catalysis. Pushing site engineering further, single-atom sites embedded in an sp^2^-carbon COF were shown to catalyze both exogenous and *in situ* H_2_O_2_ into ·OH under mild conditions, while simultaneously disrupting bacterial iron homeostasis to induce “iron poisoning” and lipid peroxidation.^205^ This strategy achieved effective bacterial elimination and accelerated wound healing in *in vivo* infection models, opening a new avenue for integrating metabolic intervention mechanisms into COF antibacterial systems. Similarly, a copper phthalocyanine-based COF incorporated multi-scale active centres consisting of Cu–N_4_ single-atom sites and Ag nanoparticles.^206^ The single-atom sites enabled efficient electron capture, while the Ag nanoparticles provided plasmonic and ionic synergy. As a result, this system achieved >99.9% broad-spectrum sterilization and could also be adapted into an electrochemical biosensor platform, enabling the integration of sterilization and monitoring. Finally, COF nanosheets function as light-responsive nanoenzyme enhancers, leveraging their two-dimensional short-range charge transport and large specific surface area.^207^ By enhancing oxidase-like activity, they simultaneously promote sensing, antibiotic degradation, and antibacterial effects, thereby forming a closed electron cycle spanning “generation–transfer–utilization”.

To advance toward clinical translation, antibacterial materials must not only “kill quickly and steadily” but also demonstrate compatibility with biological environments. This requires degradability, low toxicity, facile application/formability, and reliable performance under realistic conditions such as hypoxia, exudate, and mechanical disturbance. For example, degradable porphyrin-based COFs integrate dynamic bonds or “chain-breaking units” into the skeleton, which are selectively cleaved by ROS or hypoxic stimuli, enabling adaptive release and redistribution of photosensitizer fragments in the infection microenvironment.^208^ Through the synergistic action of PDT and CDT/Fenton-like pathways, these systems effectively overcome hypoxia-induced limitations. In wound infection models, they markedly reduce bacterial colonies, suppress inflammation, and accelerate tissue repair, achieving a balance between “high efficiency and biosafety”. Alternatively, boron-based COF-1, featuring a simple B–O backbone and mild surface chemistry, offers excellent blood and cellular compatibility while maintaining visible-light-driven ^1^O_2_/·O_2_^−^ generation capacity. It exhibits broad-spectrum antibacterial activity and low toxicity in both *in vitro* and small-animal experiments, demonstrating that the “simple structure–reliable function” design strategy is more readily applicable in quasi-clinical scenarios.^209^ Meanwhile, the TCPP–Cu D–A COF hydrogel, which embeds porphyrin photosensitizers and natural curcumin into a three-dimensional network, provides wound sites with adhesion, moisture retention, and mechanical buffering. This hydrogel enables continuous *in situ* ROS release, leading to near-complete bacterial clearance in infected wounds while simultaneously promoting re-epithelialization. Such integration of “material morphology–photocatalytic effect–tissue repair” exemplifies a holistic design strategy for therapeutic biomaterials.^210^

The promising performance of COFs in photodynamic therapy and antibacterial applications, primarily observed in simplified *in vitro* models,^180,182^ must be critically evaluated against the challenges of the complex biological microenvironment for *in vivo* translation. Key hurdles include: (i) protein corona formation: surface coating by biomolecules alters colloidal properties, cellular uptake, and biodistribution, potentially triggering immune responses.^183^ (ii) ROS quenching: endogenous antioxidants, enzymes, and hypoxic conditions in tumors can severely deplete therapeutic ROS, especially ^1^O_2_.^185,186^ (iii) Biodegradation and long-term safety: the metabolic fate, degradation products, and long-term biocompatibility of COFs remain largely unknown, with linkage stability being paramount. (iv) Barrier penetration: effective delivery requires overcoming dense biological barriers, such as biofilms for antibacterial action^191^ or vascular and tissue barriers for tumor therapy. Emerging studies are beginning to address these complexities, for example, by evaluating COF behavior in serum-containing media^182^ or biofilm models.^191^ To advance toward clinical use, future design strategies should focus on: first, intelligent surface engineering to control protein corona formation and enhance targeting. Second, designing microenvironment-responsive or self-oxygenating COFs.^186,193^ Third, integrating imaging capabilities for theranostic applications. Fourth, establishing robust preclinical evaluation frameworks to systematically assess pharmacokinetics, biodistribution, and long-term toxicity *in vivo*.

## 5 Conclusions

Over the past decade, COFs have evolved from an emerging curiosity into a versatile class of crystalline porous materials with far-reaching implications for photocatalytic oxidation chemistry. Distinguished by their long-range ordered yet modular architectures, high surface areas, and tunable electronic structures, COFs have proven to be fertile grounds for harnessing solar energy and mediating the generation of ROS. These reactive intermediates—^1^O_2_, ·O_2_^−^, ·OH, and other high-valent oxygen species—play decisive roles in driving a wide array of oxidative transformations relevant to environmental remediation, chemical synthesis, and biomedicine.

A central theme that emerges from recent research is the capacity of COFs to act as “designer photocatalysts.” Their modular construction from organic linkers and nodes allows precise engineering of frontier orbital energies, charge separation pathways, and the density and distribution of active sites. Through judicious design—such as extending π-conjugation to improve visible-light absorption or incorporating electron-donating and -withdrawing motifs to tune donor–acceptor interactions—researchers can tailor the excited-state properties that govern ROS generation. The introduction of heteroatoms, such as nitrogen or sulfur, or coordination with single-atom metal sites, provides additional levers to control charge localization, spin density distribution, and reaction selectivity. Equally important is the rational manipulation of porosity and topology. COFs offer an unparalleled opportunity to construct ordered porous frameworks that enhance molecular diffusion, facilitate reactant access to catalytic sites, and provide spatial confinement that stabilizes reactive intermediates. Such structural control is central to modulating the relative contributions of type I and type II ROS pathways, thereby enabling a level of selectivity rarely achievable with conventional inorganic semiconductors.

The structural tunability of COFs has been harnessed to realize diverse photocatalytic oxidation processes under mild and environmentally benign conditions. A growing body of studies has demonstrated that COFs can achieve high turnover frequencies and excellent selectivities in the oxidation of organic sulfides to sulfoxides or sulfones, the transformation of amines into imines, and the selective oxidation of alcohols to aldehydes and ketones. These reactions are cornerstones of fine-chemical and pharmaceutical synthesis, highlighting the practical importance of COFs as sustainable alternatives to stoichiometric oxidants or precious-metal catalysts.

Beyond traditional organic synthesis, COF-mediated ROS generation has been explored for environmental purification, particularly in the degradation of persistent organic pollutants such as dyes, pesticides, and antibiotic residues. The ordered pore channels and hydrophilic functionalization of certain frameworks allow efficient mass transfer of water-soluble contaminants to active sites, while the photo-induced ROS enable non-selective degradation of complex pollutants into less harmful by-products. In biomedical research, COFs have shown promise as multifunctional platforms for photodynamic and photothermal therapies. By rationally incorporating photoactive chromophores and catalytic motifs, COFs can generate cytotoxic ROS *in situ* to eradicate pathogens or induce cancer cell apoptosis, while their tunable pores can encapsulate therapeutic agents for synergistic treatment.

Collectively, the progress summarized above converges on and validates the “structure–ROS–substrate” paradigm as more than a descriptive observation; it is the foundational framework that encapsulates the field's core advancement. The unique contribution of this paradigm lies in its integrative and predictive power. It transcends traditional analytical tools by explicitly linking the initial photophysical event to the final chemical outcome determined by the ROS–substrate encounter within the structural confines. This perspective allows the “structure–ROS–substrate” paradigm to serve a dual function: as an interpretative tool to decode complex selectivity patterns and reconcile disparate catalytic data, and as a forward-looking design blueprint. It shifts the design objective from optimizing isolated properties to purposefully engineering the dynamic interplay within the entire “structure–ROS–substrate” triad to achieve a target photocatalytic function.

Despite this remarkable progress, several challenges remain before COF-based photocatalysts can be deployed widely in industrial or biomedical contexts. A persistent hurdle is the scalable, cost-effective synthesis of highly crystalline and chemically robust frameworks. Many state-of-the-art COFs rely on elaborate organic monomers or demanding reaction conditions, which can limit reproducibility and hinder large-scale implementation. Developing more sustainable synthetic routes—such as low-temperature, aqueous-phase methods or solvent-free mechanochemical strategies—will be vital for broadening accessibility.

Another critical challenge lies in the limited understanding of the mechanistic intricacies that underlie ROS formation and utilization. While techniques such as ESR, *operando* spectroscopy, and computational modeling have provided invaluable insights into the generation of ·O_2_^−^, ^1^O_2_, and ·OH, a complete picture of the dynamic interplay between different ROS pathways is still emerging. In particular, the relative contributions of type I *versus* type II processes, and the role of framework defects or guest–host interactions in directing selectivity, require deeper exploration. Stability is also a recurring concern. Although certain COFs exhibit remarkable tolerance to light irradiation, reactive oxygen environments, and acidic or basic conditions, many others suffer from gradual degradation, loss of crystallinity, or deactivation of catalytic sites. Long-term operational stability, especially under continuous flow or in complex matrices such as industrial effluents and biological fluids, remains a decisive factor for real-world adoption.

More critically, mechanistic understanding of COF-driven photocatalysis is still constrained by the lack of effective *in situ* ROS detection tools. Current approaches, including radical scavenging, ESR spin trapping, and theoretical simulations, provide valuable but often indirect or static insights. Developing advanced characterization techniques with high spatial and temporal resolution—such as *in situ* spectroscopies, single-molecule imaging, isotope labeling, or electrochemical probing—will be essential to dynamically monitor ROS generation, evolution, and interplay with substrates. Such breakthroughs would allow a deeper elucidation of the structure–ROS–substrate relationships and guide the precise design of next-generation COFs.

A paramount challenge that curtails the transition from empirical discovery to rational design in COF photocatalysis is the dynamic complexity of the “structure–ROS–substrate” interface, which remains obscured by conventional *ex situ* characterization. While techniques such as EPR spin trapping and quenching experiments provide essential proof of ROS presence, they offer limited temporal and spatial resolution into the sequence of events: How exactly does charge separation at a donor–acceptor junction dictate the branching ratio between singlet oxygen and superoxide? What is the lifetime and diffusion length of key intermediates within the confined pore before reacting? To decode this black box, the field must pivot towards multimodal *in situ* and *operando* diagnostics. For example, time-resolved surface-enhanced Raman spectroscopy could capture vibrational fingerprints of transient surface-bound peroxo or radical species during illumination. Ultrafast transient absorption microscopy would spatially map exciton diffusion and charge separation efficiency across different crystalline domains or defects. *In situ* liquid-phase transmission electron microscopy could, in principle, visualize the ingress of substrate molecules into pores and their transformation at active sites. Furthermore, coupling isotope-labeled mass spectrometry with photoelectrochemical measurements can quantitatively deconvolute the contributions of water oxidation *versus* oxygen reduction pathways to H_2_O_2_ production. By orchestrating such a suite of techniques, researchers can move beyond correlative “activity–structure” relationships and establish causal mechanistic maps, ultimately enabling the predictive design of COFs with programmed ROS output and substrate selectivity.

Therefore, the path forward must be explicitly guided by the “structure–ROS–substrate” paradigm. Looking ahead, several avenues hold promise for advancing COF-based photocatalytic oxidation. First, synthetic innovation must continue to lower the barriers to access highly crystalline, functionalized frameworks. This includes the development of scalable, green synthetic approaches that leverage renewable feedstocks and avoid harsh reagents. Advances in reticular chemistry, such as topological design, linker engineering, and post-synthetic modification, will be central to constructing COFs with tailored light-absorbing motifs, optimized charge-transport pathways, and controlled porosity.

Second, the rational design of COFs for selective ROS pathways represents a particularly exciting frontier. By controlling the electronic structure through donor–acceptor engineering, heteroatom doping, or the incorporation of catalytic sites such as single-atom metals, researchers can favor the generation of specific ROS. Such control could significantly enhance the energy efficiency and selectivity of photocatalytic oxidation reactions, opening the door to more sustainable processes for chemical synthesis and environmental remediation.

Third, advancing mechanistic understanding remains essential. Integrating transient absorption spectroscopy, *in situ* or *operando* spectroscopies, and theoretical modeling will be crucial to unravel the fundamental principles that dictate exciton dissociation, charge transport, and ROS reactivity within COFs. This knowledge will not only guide the rational design of next-generation frameworks but also help establish universal design rules for tailoring photocatalysts at the molecular level.

Fourth, future studies should increasingly consider realistic and complex environments. Many current demonstrations employ model organic dyes or simplified aqueous systems, but practical applications demand robustness under diverse conditions, including the presence of salts, natural organic matter, and fluctuating pH. Translating laboratory findings into industrial water treatment, environmental remediation, or biomedical therapy requires comprehensive evaluation of stability, reusability, and scalability.

Truly advancing COF photocatalysis from a laboratory novelty to a technology with net environmental benefit demands a paradigm shift: performance must be evaluated in tandem with sustainability across the entire life cycle. This necessitates the formal adoption of Green Chemistry principles and rigorous Life Cycle Assessment (LCA) as non-negotiable pillars of future research. First, synthetic elegance must be weighed against environmental cost. The field should celebrate and prioritize COFs derived from biomass-based monomers or synthesized *via* atom-economical, solvent-free mechanochemistry, even if their initial activity metrics are modest, as their scalable and low-footprint production could offer greater overall sustainability. Second, the circularity of COFs must be proactively engineered. This includes designing frameworks with embedded recyclability, such as COFs that can be depolymerized into pure monomers for re-synthesis, or frameworks with triggered biodegradability *via* pH- or enzyme-cleavable linkers for safe disposal. Concurrently, pioneering studies must begin to assess the long-term environmental fate of COF nanoparticles, including their ecotoxicity, potential for bioaccumulation, and transformation products in aquatic and soil systems. Funding agencies and journals can accelerate this transition by incentivizing research that reports not only high conversion yields but also Environmental Factor (E-factor) of synthesis and end-of-life impact data. By embedding these holistic metrics into the core R&D ethos, the COF community can ensure its innovations contribute authentically to a circular and green chemical economy, rather than solving one environmental problem while inadvertently creating another.

Fifth, integration with other functional materials provides a fertile path forward. Constructing heterojunctions with semiconductors, coupling COFs with metal–organic frameworks (MOFs), or embedding single-atom catalysts can endow COFs with new functionalities and further enhance ROS yields. Similarly, combining COFs with biomolecules, enzymes, or responsive polymers may enable smart platforms for targeted therapy or controlled release applications, bridging the gap between materials science and biomedicine.

Finally, the field would benefit from embracing computational and data-driven approaches. Machine learning, high-throughput screening, and multiscale modeling could accelerate the discovery of optimal building blocks and linkages, providing predictive insights into how structural variations influence ROS generation and stability. Such approaches, coupled with rigorous life-cycle assessments, will be indispensable for guiding COFs from academic laboratories to real-world deployment.

In summary, COF-based photocatalysts embody a convergence of molecular design precision, structural order, and functional versatility. Their ability to regulate the formation and utilization of ROS under mild, sustainable conditions sets them apart from conventional photocatalysts and positions them as promising candidates for applications ranging from selective organic synthesis to pollutant degradation and biomedical therapy. Most importantly, the research journey has crystallized the “structure–ROS–substrate” paradigm as the central, unifying framework that both explains past successes and illuminates the path ahead. By deepening our mechanistic understanding, innovating synthetic strategies, and fostering interdisciplinary integration, the community is well placed to transform COFs from an emerging concept into a cornerstone of next-generation oxidation technologies. The continuing dialogue between fundamental science and applied engineering will be pivotal in unlocking the full potential of COFs to address pressing challenges in energy, sustainability, and health.

## Author contributions

Q. Fang and C. Liu conceived the idea for this review. T. Sun and R. Wang drafted the manuscript, R. Guan and L. Wang conducted data search and verification. X. Cheng and T. Zhong polished the language of the manuscript. Finally, all authors revised the manuscript.

## Conflicts of interest

There are no conflicts to declare.

## Data Availability

No primary research results, software or code have been included and no new data were generated or analysed as part of this review.
